# Supplementary material for: Two Aldehyde Clearance Systems Are Essential to Prevent Lethal Formaldehyde Accumulation in Mice and Humans
Source: Mol Cell. 2020 Dec 17;80(6):996–1012.e9. doi: 10.1016/j.molcel.2020.10.012 (PMC7758861; doi:10.1016/j.molcel.2020.10.012)
Supplement: Document S1. Figures S1–S7 and Tables S1–S7 [file mmc1.pdf]

Molecular Cell, Volume *80*

Supplemental Information

Two Aldehyde Clearance Systems Are Essential to Prevent Lethal Formaldehyde Accumulation in Mice and Humans

Felix A. Dingler, Meng Wang, Anfeng Mu, Christopher L. Millington, Nina Oberbeck, Sam Watcham, Lucas B. Pontel, Ashley N. Kamimae-Lanning, Frederic Langevin, Camille Nadler, Rebecca L. Cordell, Paul S. Monks, Rui Yu, Nicola K. Wilson, Asuka Hira, Kenichi Yoshida, Minako Mori, Yusuke Okamoto, Yusuke Okuno, Hideki Muramatsu, Yuichi Shiraishi, Masayuki Kobayashi, Toshinori Moriguchi, Tomoo Osumi, Motohiro Kato, Satoru Miyano, Etsuro Ito, Seiji Kojima, Hiromasa Yabe, Miharu Yabe, Keitaro Matsuo, Seishi Ogawa, Berthold Göttgens, Michael R.G. Hodskinson, Minoru Takata, and Ketan J. Patel

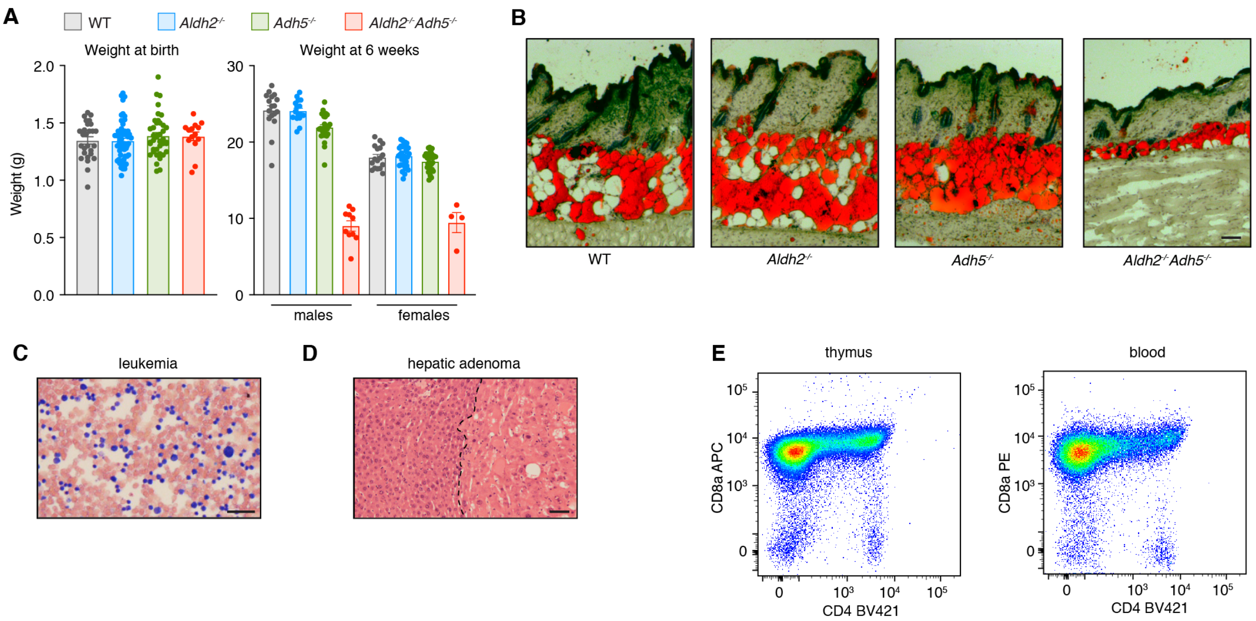

Figure S1, relating to Figure 1. Postnatal lethality of *Aldh2*$^{-/-}$*Adh5*$^{-/-}$ mice

(A) Mouse weight at birth (*n* = 27, 57, 40, 14; left to right) and at 6 weeks (*n* = 17, 16, 26, 10, 17, 33, 28, 4; left to right). **(B)** Oil red O staining of subcutaneous fat in dorsal skin. Scale bar: 100 μm. **(C)** Blood film of *Aldh2*$^{-/-}$*Adh5*$^{-/-}$ mouse which died at 38 weeks showing leukemia. Scale bar: 50 μm. **(D)** Hepatic adenoma in same mouse. Scale bar: 100 μm. **(E)** CD3^{-} CD8^{+} T cell leukemia seen in a 42-week-old mouse.

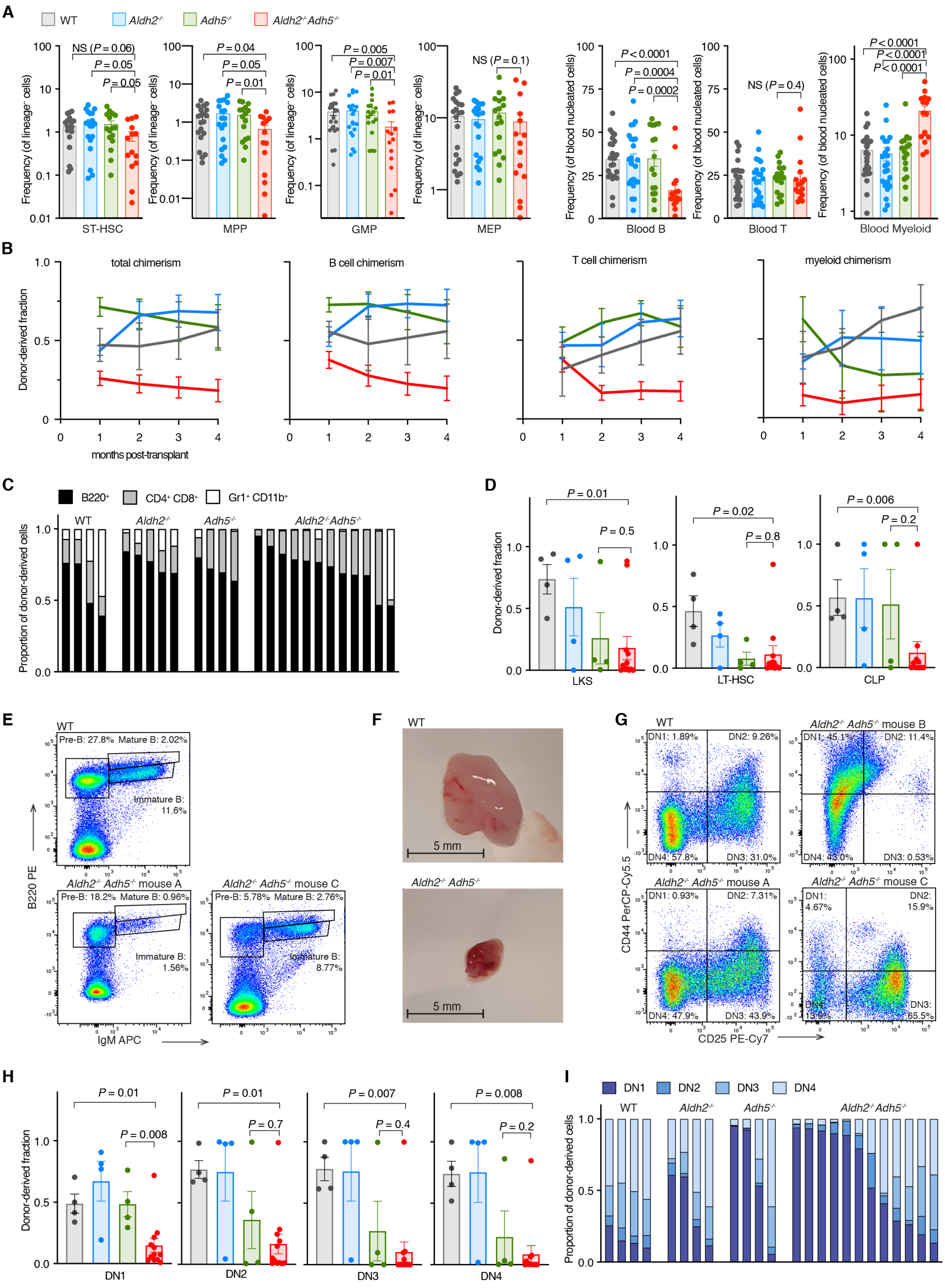

Figure S2, relating to Figure 2 and 3. Disrupted hematopoiesis and lymphoid development in *Aldh2*$^{-/-}$*Adh5*$^{-/-}$ mice.

(A) Quantification of bone marrow ST-HSC, MPP, GMP and MEP populations, and blood B, T lymphocyte, and myeloid (CD11b$^+$ Gr-1$^+$) populations in *Aldh2*$^{-/-}$*Adh5*$^{-/-}$ mice with age matched controls (data shown as mean ± SEM, n = 24, 20, 17 and 17 mice,

left to right). **(B)** Long-term competitive reconstitution experiment showing serial blood samples of transplanted mice analyzed for contribution to B220+ (B cell), CD4+/CD8+ (T cell) and Gr1+/Mac-1+ (myeloid) compartment. Fraction of donor-derived cells, donor/(donor+competitor), shown as mean ± SEM, n = 4, 5, 4, 12 recipients for *WT, Aldh2*$^{-/-}$*, Adh5*$^{-/-}$*, Aldh2*$^{-/-}$ *Adh5*$^{-/-}$ donors respectively. **(C)** Output bias of donor bone marrow assessed at 4 months. **(D)** Contribution to LKS, long-term HSC, and common lymphoid progenitor population at 4 months (n = 4, 4, 4, 12). **(E)** Flow cytometry plots of bone marrow B cell populations showing the variation observed in *Aldh2*$^{-/-}$*Adh5*$^{-/-}$ mice. Mouse A shows preferential loss of immature and mature B cells, compared to loss of pre-B cells in mouse C. **(F)** Macroscopic picture of thymus in a WT and an *Aldh2*$^{-/-}$*Adh5*$^{-/-}$ mouse. **(G)** Flow cytometry plots of DN populations defined by CD44 and CD25 expression from three *Aldh2*$^{-/-}$*Adh5*$^{-/-}$ mice aged 2-4 weeks. Mouse A shows DN population pattern comparable to WT thymus, mouse B thymus shows significant depletion of DN2 and 3 populations, whereas mouse C thymus shows expansion of DN2 and 3 populations. **(H)** Fraction of donor-derived cells amongst donor- or competitor-derived cells in the double-negative fraction of the thymus. Data shown as mean ± SEM, n = 4, 4, 4 and 12 recipients. **(I)** Distribution of donor-derived cells amongst DN fractions in thymus of competitively repopulated recipients (n = 4, 4, 4, 12).

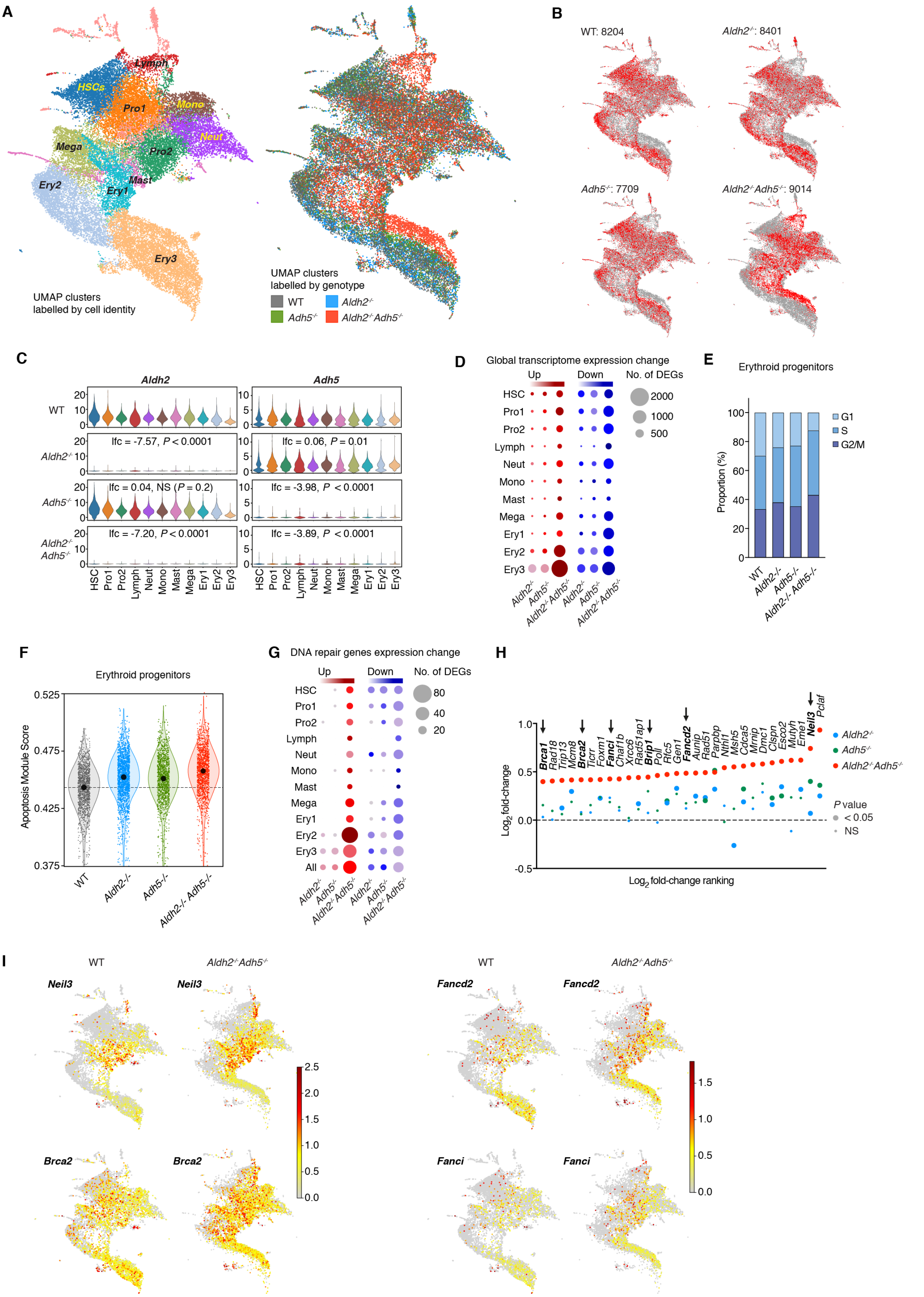

Figure S3, relating to Figures 2, 3 and 4. Single cell RNAseq analysis of hematopoietic stem and progenitor cells in *Aldh2$^{-/-}$Adh5$^{-/-}$* mice.

(A) Two-dimensional visualization by UMAP of bone marrow (Lin^{-} c-Kit^{+} and Lin^{-} Sca-1^{+} c-Kit^{lo}) transcriptomes combined from all 4 genotypes. Each dot represents a

transcriptome from a single cell. On the left the clusters are colored by HSC or lineage-primed progenitor identity assigned by expression of lineage-specific marker genes. On the right transcriptomes are colored by genotype to highlight variation in distribution between the *Aldh2*$^{-/-}$*Adh5*$^{-/-}$ and controls across the UMAP clusters. **(B)** Transcriptomes of individual genotypes highlighted in red over the background of all genotypes in grey. **(C)** Ensemble of violin plots showing normalized gene expression score for *Aldh2* and *Adh5* (number of UMIs per 10000) for each cluster and genotype. **(D)** Analysis of differentially expressed genes, circle size denoting number of genes passing filter and color intensity reflecting median fold change. **(E)** Cell cycle analysis of erythroid progenitors based on transcriptome profile. **(F)** Apoptosis module score for erythroid progenitors. **(G)** Analysis of differentially expressed genes annotated as DNA repair gene in gene ontology, circle size denoting number of genes passing filter and color intensity reflecting median fold change. **(H)** Top differentially expressed DNA repair genes by genotype. Genes implicated in crosslink repair are highlighted with arrows. **(I)** Select examples of gene expression plotted on the UMAP landscape of WT and *Aldh2*$^{-/-}$ *Adh5*$^{-/-}$ hematopoietic stem and progenitor cells. See also Tables S3, S4, and S6.

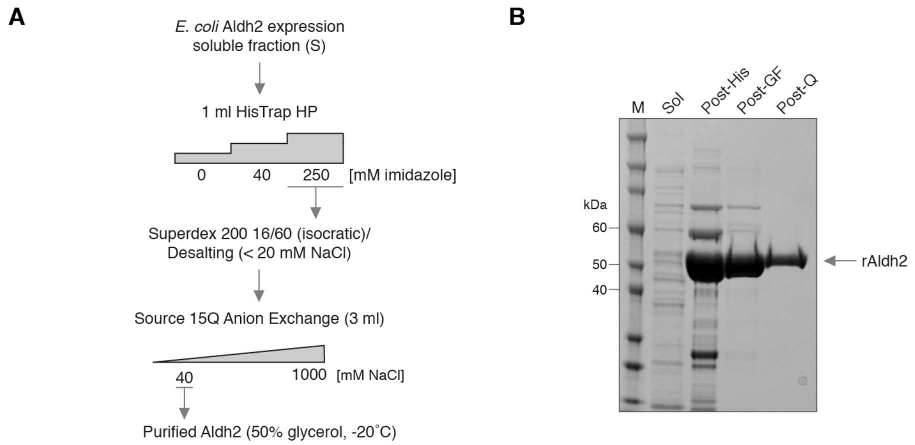

Figure S4, relating to Figure 4. Purification of recombinant mouse ALDH2

(A) Scheme of purification strategy. **(B)** Coomassie stained SDS-PAGE of fractions: M, size marker; Sol, soluble fraction; Post-His, pooled chelate eluate; Post-GF, pooled gel filtration eluate; post-Q, Q anion exchange eluate.

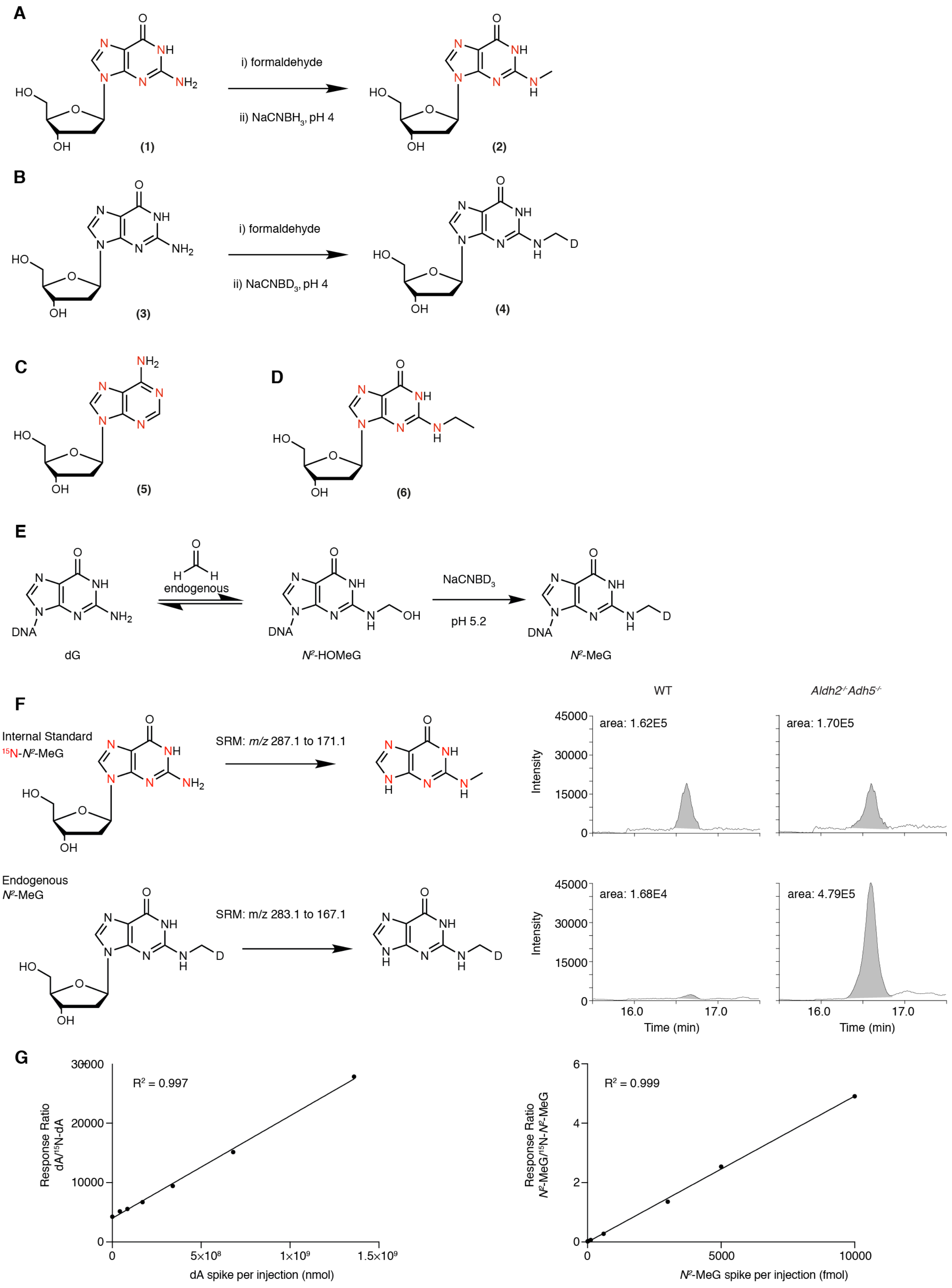

Figure S5, relating to Figure 5. Chemical synthesis of formaldehyde-DNA adduct standards and quantification of *N*^{2}-MeG via mass spectrometry.

(A) Reaction to make ^{15}N-*N*^{2}-MeG (red letter = ^{15}N): i) formaldehyde, ii) NaCNBH$_3$, pH4. **(B)** Reaction to make *N*^{2}-MeG, i) formaldehyde, ii) NaCNBD$_3$, pH4. **(C)** Structure of the

^{15}N-dA standard used for *in situ* quantification of total injected digested DNA per MS run (red letter = ^{15}N). **(D)** Structure of the ^{15}N-N^2-EtG standard used in this study (red letter = ^{15}N). **(E)** Reaction scheme for the stabilization of formaldehyde mono-adducts on genomic DNA. Endogenous formaldehyde can react at the N^2-position of guanine to form the hydroxymethyl mono-adduct, however this reaction is reversable. We used $NaCNBD_3$ to reduce the N^2-HOMeG adduct to the stable N^2-MeG (D omitted for clarity). **(F)** Left: MS/MS transitions for the internal standard and endogenous N^2-MeG used in the SRM method. Right: representative and corresponding MS traces for WT and *Aldh2*$^{-/-}$*Adh5*$^{-/-}$ mouse tissue (red letter = ^{15}N). **(G)** Standard curve examples for the MS/MS data presented in this paper. Fresh standard curves were prepared with every batch of digested genomic DNA samples.

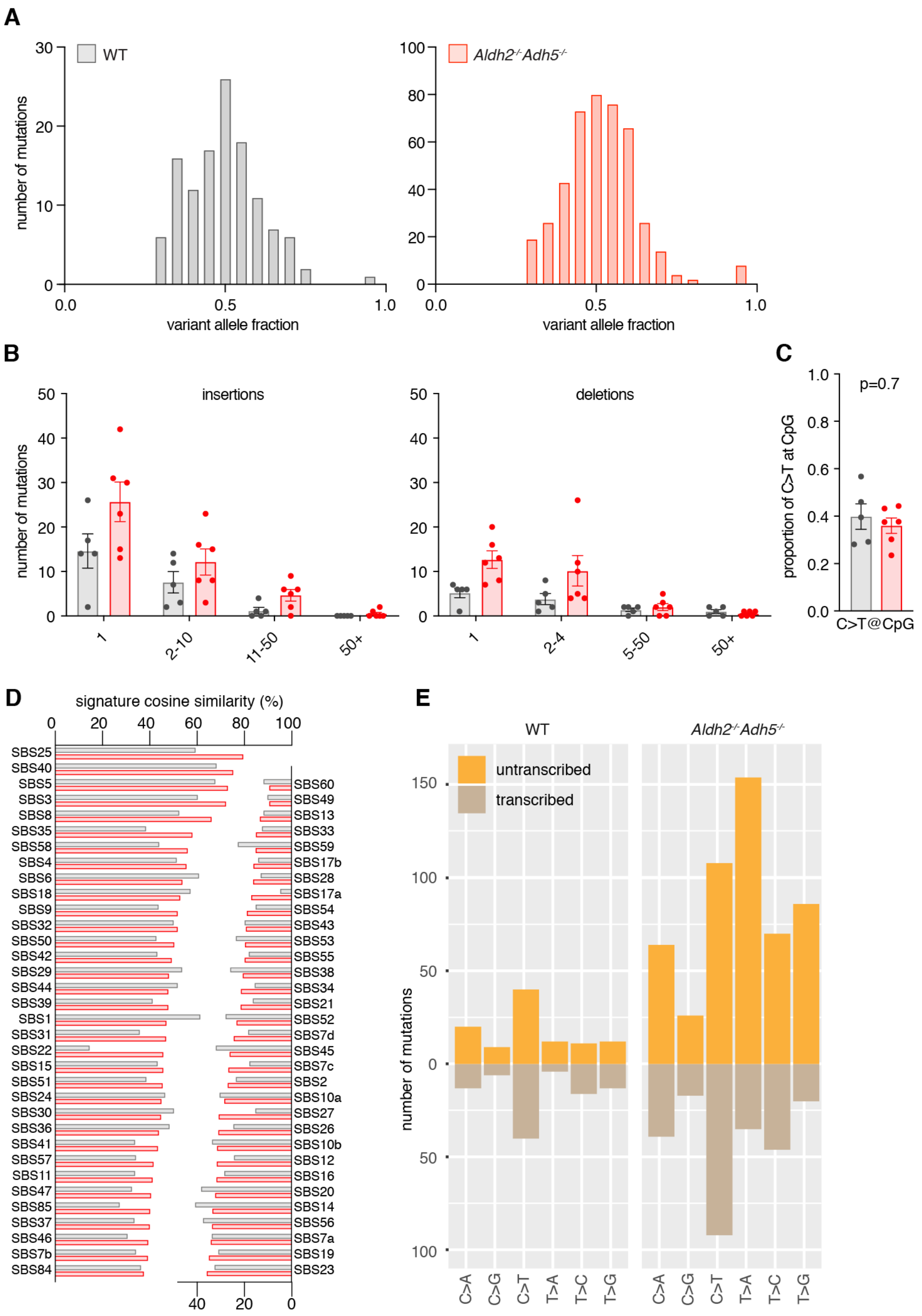

Figure S6, relating to Figure 6. Mutational signature of HSPC clones

(A) Representative histograms showing variant allele frequencies for final filtered (analyzed) variants of WT and *Aldh2$^{-/-}$Adh5$^{-/-}$* HSPC clones. **(B)** Size distribution of insertions (left) and deletions (right) in analyzed HSPC clones. **(C)** Proportion of C to T mutations occurring in CpG sequence context. **(D)** Cosine similarity between mutational profile of WT and *Aldh2$^{-/-}$Adh5$^{-/-}$*HSPCs and published COSMIC mutation signatures. **(E)** Histograms of single base substitutions falling into genes indicating whether the pyrimidine base of the pair is on the coding or noncoding strand.

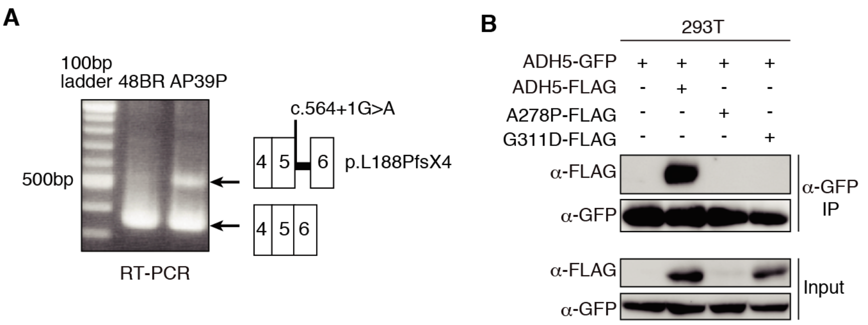

Figure S7, relating to Figure 7. Characterization of *ADH5* patient mutations.
(A) RT-PCR analysis of *ADH5* transcripts in fibroblasts from patient P1. cDNA from patient or control 48BR cells was amplified using primers that cover exon 4 to 6 of *ADH5*. The c.564+1G>A mutation resulted in retention of intron 5 and p.L188PfsX4. **(B)** Effects of *ADH5* missense variants on expression and dimer formation. 293T cells transfected with indicated plasmids were lysed, subjected to coimmunoprecipitation by anti-GFP antibody, and precipitated proteins were detected by western blotting with anti-FLAG antibody. Note that the FLAG-tagged A278P ADH5 variant was barely detected in the lysate.

Table S1, related to Figure 1B.
Incidence of cancer-related deaths in *Aldh2*$^{-/-}$*Adh5*$^{-/-}$ mice.

Number	Age (weeks)	Cause of death
1	29	$CD4^{+}$ T cell leukemia
2	36	Liver tumor
3	38	Liver tumor and leukemia
4	42	$CD8^{+}$ leukemia and thymoma
5	43	Thymoma

Table S2, related to Figure 2.
Enriched GO terms of top 100 differentially expressed genes in the *Aldh2*$^{-/-}$*Adh5*$^{-/-}$ erythroid progenitors (Ery3)

GO term_name	term_id	adjusted_p_value
intrinsic apoptotic signaling pathway in response to DNA damage	GO:0008630	0.0156173
organonitrogen compound biosynthetic process	GO:1901566	0.02766473
intrinsic apoptotic signaling pathway by p53 class mediator	GO:0072332	0.03454527
signal transduction by p53 class mediator	GO:0072331	0.04324361
sequestering of actin monomers	GO:0042989	0.04836535

Table S3, related to Figure 2.

Enriched GO terms of top 100 differentially expressed genes in the *Aldh2*$^{-/-}$*Adh5*$^{-/-}$ HSC cluster

GO term_name	term_id	adjusted_p_value
response to oxygen-containing compound	GO:1901700	1.05E-08
cellular response to oxygen-containing compound	GO:1901701	1.36E-07
response to organic substance	GO:0010033	1.51E-07
cellular response to chemical stimulus	GO:0070887	2.31E-07
cellular response to organic substance	GO:0071310	1.01992E-06
regulation of immune system process	GO:0002682	1.99803E-06
regulation of multicellular organismal process	GO:0051239	4.44607E-06
immune system process	GO:0002376	6.12521E-06
response to endogenous stimulus	GO:0009719	6.60435E-06
myeloid cell differentiation	GO:0030099	1.28529E-05
positive regulation of multicellular organismal process	GO:0051240	3.40627E-05
regulation of myeloid cell differentiation	GO:0045637	4.49602E-05
regulation of localization	GO:0032879	6.35689E-05
response to nitrogen compound	GO:1901698	8.19104E-05
response to organonitrogen compound	GO:0010243	0.000100321
cell differentiation	GO:0030154	0.000161578
regulation of hemopoiesis	GO:1903706	0.000183388
cellular developmental process	GO:0048869	0.000273058
regulation of developmental process	GO:0050793	0.000371603
regulation of multicellular organismal development	GO:2000026	0.000416546
cellular response to endogenous stimulus	GO:0071495	0.000615769
hemopoiesis	GO:0030097	0.00080195
positive regulation of cellular process	GO:0048522	0.000967844
negative regulation of cellular process	GO:0048523	0.001286093
regulation of cell migration	GO:0030334	0.001366403
regulation of biological quality	GO:0065008	0.001558247
hematopoietic or lymphoid organ development	GO:0048534	0.001599819
cell migration	GO:0016477	0.001861214
response to chemical	GO:0042221	0.002013742
localization of cell	GO:0051674	0.002034977
cell motility	GO:0048870	0.002034977
regulation of cell population proliferation	GO:0042127	0.002081353
negative regulation of multicellular organismal process	GO:0051241	0.002183892
heterophilic cell-cell adhesion via plasma membrane cell adhesion molecules	GO:0007157	0.002214536
cell population proliferation	GO:0008283	0.002418803
cellular response to calcium ion	GO:0071277	0.002628424
response to stress	GO:0006950	0.002668339
regulation of cell motility	GO:2000145	0.002785447
locomotion	GO:0040011	0.002986195
immune system development	GO:0002520	0.003542013
positive regulation of gene expression	GO:0010628	0.003877516
response to lipid	GO:0033993	0.004091222
positive regulation of developmental process	GO:0051094	0.004684961
positive regulation of macromolecule metabolic process	GO:0010604	0.00485466
regulation of locomotion	GO:0040012	0.005157507
response to lipopolysaccharide	GO:0032496	0.005387423
positive regulation of nitrogen compound metabolic process	GO:0051173	0.005442722
myeloid leukocyte differentiation	GO:0002573	0.005552054
positive regulation of cellular metabolic process	GO:0031325	0.005951117
cell surface receptor signaling pathway	GO:0007166	0.005964847
negative regulation of biological process	GO:0048519	0.006029472

GO term_name	term_id	adjusted_p_value
movement of cell or subcellular component	GO:0006928	0.006827294
regulation of cell activation	GO:0050865	0.007000214
response to molecule of bacterial origin	GO:0002237	0.007649681
regulation of cellular component movement	GO:0051270	0.00944446
positive regulation of metabolic process	GO:0009893	0.009520947
cellular response to glucose stimulus	GO:0071333	0.009643389
cellular response to hexose stimulus	GO:0071331	0.010038369
positive regulation of biological process	GO:0048518	0.010537982
cellular response to monosaccharide stimulus	GO:0071326	0.010868856
cell activation	GO:0001775	0.011690641
inflammatory response	GO:0006954	0.013768332
cellular response to carbohydrate stimulus	GO:0071322	0.015929948
positive regulation of immune system process	GO:0002684	0.016756211
positive regulation of cell-cell adhesion	GO:0022409	0.017292424
cell death	GO:0008219	0.017759915
response to external stimulus	GO:0009605	0.019372058
regulation of leukocyte activation	GO:0002694	0.019972363
cellular glucose homeostasis	GO:0001678	0.022029729
amyloid-beta clearance	GO:0097242	0.022835694
enzyme linked receptor protein signaling pathway	GO:0007167	0.024718074
leukocyte differentiation	GO:0002521	0.026421044
response to abiotic stimulus	GO:0009628	0.027722184
cellular response to metal ion	GO:0071248	0.030962982
MAPK cascade	GO:0000165	0.032933037
homeostatic process	GO:0042592	0.037793755
regulation of cell death	GO:0010941	0.038337117
regulation of transcription by RNA polymerase II	GO:0006357	0.039925822
signal transduction by protein phosphorylation	GO:0023014	0.042851358
defense response	GO:0006952	0.044757433
response to hormone	GO:0009725	0.047719264

Table S4, related to Figure 2.

Top 100 upregulated genes in HSC subclusters (relative to union of other subclusters). Genes used to assign lineage bias highlighted in bold.

	**cluster 0**	**cluster 1**	**cluster 2**	**cluster 3**	**cluster 4**	**cluster 5**	**cluster 6**
1	Ifitm1	Wfdc17	Cdk6	Fut8	Hist1h2bc	Ifit1	C1qa
2	Pdzk1ip1	**Ighm**	Plac8	Cdk6	Vim	Isg15	C1qb
3	Gng11	**Dntt**	**Mpo**	Nkg7	Ccnb2	Oasl2	C1qc
4	Mllt3	H2afy	Sell	Sdsl	Rps2	Rsad2	Apoe
5	Hacd4	Flt3	Ccl9	Muc13	Ptms	Ifi44	Ctsb
6	Ifitm3	Satb1	Nkg7	Eif5a	Hist1h1c	Ifitm3	Fcer1g
7	Malat1	Emb	Cd48	Nme1	Lgals1	Rtp4	Tmsb4x
8	**Procr**	Mef2c	Serpinb1a	Dctpp1	Cenpe	Trim30a	Psap
9	**Mpl**	Cd52	Tespa1	Ldha	H2afv	Iigp1	Tyrobp
10	Rbp1	Il12a	H2afy	Atp5g1	Txn1	Usp18	Marcks
11	Hlf	Ramp1	Prtn3	Cdca7	Dap	Ifit3	Selenop
12	Ltb	Ddx4	Adgrg3	Hmgb3	Hist1h4i	Slfn5	Axl
13	**Mecom**	Tmsb10	Bex6	Apoe	Rps17	Gm4951	Ctss
14	Socs2	Sdc1	Gm20342	Mif	Srgn	Irf7	Lgmn
15	Apoe	Wfdc18	BC035044	Gclm	Knstrn	Zbp1	Hpgd
16	Cdkn1c	Gpr171	Calr	Fabp5	Rpl41	Xaf1	Vcam1
17	Ly6a	Smad7	Cmtm7	Txn1	Cenpf	Bst2	Mrc1
18	Tbxas1	Ncf1	Cd34	Srsf3	Rpl14	Igtp	Fth1
19	Cd63	Rps24	Ctsg	Cd48	Cox6b2	Mndal	Sirpa
20	Gimap1	H2-Ob	Slco3a1	Srgn	Tmsb4x	Parp9	Aif1
21	Itsn1	Cd34	Tyrobp	Atpif1	Plac8	Gbp7	Fcna
22	Cavin3	Rasa4	Lat2	Vamp5	Cd9	Ifi203	Sdc3
23	Bex1	Notch1	Fut8	Tgfb1	Cdca8	Ly6a	Mafb
24	Tcf15	Cd37	Ndufa4	Sell	Mki67	Parp14	Csf1r
25	Esam	Sema3d	Bin1	Hnrnpd	Ddx39	Panx2	Trf
26	Car2	St8sia4	Phf14	Rps17	Rpl7a	Ly6e	Mpeg1
27	Mycn	Mn1	Zeb2	Ncl	S100a10	Samd9l	Cfp
28	Wfdc2	Ccl3	Atp8b4	Cd63	Atp5b	Eif2ak2	Ms4a7
29	Selenom	Il1r1	Fam117a	Srm	Hmgb1	Stat1	Cyba
30	Upp1	Cd69	Slc16a11	Nop58	Cenpa	Oas3	Cd68
31	Trim47	Cd33	Vim	Npm1	Rpl3	Ms4a6b	Hmox1
32	Grb10	Slc35d3	Itga4	Rpl41	Hsp90ab1	Serpina3g	Igf1
33	Krt18	Egfl7	S100a10	Ranbp1	Ube2c	Herc6	Gngt2
34	Nkx2-3	Cd53	Plek	Hsp90ab1	Myl12b	Isg20	Grn
35	Rpl21	Sox4	Spns3	Rps2	**Mpo**	Ifit3b	Maf
36	Angpt1	BC035044	Myb	Clec4e	Hmgb2	Ifih1	Cybb
37	Gimap5	Myl10	Tgfb1	Slc25a5	Cd48	Trim12c	Adgre4
38	Shisa5	Arpp21	Cd52	Ppp1r14b	Lockd	Irgm1	Cd5l
39	Samd12	Gm32554	Muc13	Snrpd1	Hist1h2ae	Ifit1bl1	Sat1
40	H2-K1	Camk1d	Ramp1	Ran	Lgals9	Sp100	Ccr3
41	Serpina3g	Gm5111	Ffar2	Ctla2a	Selenoh	Ifi47	Itm2b
42	Ptgs1	Btg2	Adgrl4	Vim	Spi1	Shisa5	Clec12a
43	Pf4	Hoxa9	Rab44	Runx3	Psrc1	Mx1	Cd300c2
44	Ndn	Samsn1	Ptma	Mcm3	H1f0	Ifi204	Clec4b1
45	Vamp5	Pou2f2	Bcl2	Srsf7	Rpl28	Dhx58	Lrp1
46	Plxdc2	Fbxw4	Mdga1	Calr	H1fx	Rnf213	Ctsc
47	Txnip	Dhrs3	Pdgfrb	Igfbp4	Csrp2	Phf11b	Lpl
48	Arhgef12	Tespa1	Myc	Anp32b	Ifi27l2a	H2-T22	Fyb
49	Nceh1	Lck	Tm6sf1	4930519L02Rik	Hmgb3	Slfn8	Ctsz
50	Gimap6	Lsp1	Irf2bp2	Hspd1	Gpx1	Ms4a4b	Clec4a1
51	Col4a2	Sdc4	Rps2	Rpl14	Hist3h2a	Trim30d	Cst3
52	Csgalnact1	Malat1	Slc4a8	Dach1	Stmn1	Trafd1	Hexa
53	Cish	Pgr	Sh2d5	Ptma	Gapdh	Ifi206	Pilra
54	Gm4951	Sstr2	Ndrg1	Itga2b	Tpx2	Ddx60	Adgre1
55	Uba7	AA467197	Igfbp4	Dut	Dtnbp1	Ms4a6c	Ctsh
56	Ccnd2	Maml3	Dock10	Ybx3	Cdca3	Tor3a	Clec4a3
57	Jam3	Plac8	F630028O10Rik	Ppia	Hist1h2ac	Gbp3	Pld3
58	Tie1	Shisa8	Taok3	Slc18a2	Prtn3	Ube2l6	Ly86
59	Clec1a	Tcf4	Elf1	C1qbp	H2afz	Zufsp	Cd163
60	Myl10	Cd27	Arl11	Bin1	Sell	Ddx58	Creg1

	cluster 0	cluster 1	cluster 2	cluster 3	cluster 4	cluster 5	cluster 6
61	Npdc1	Jakmip1	Gm15657	Set	Cmtm7	Parp12	Plbd1
62	Sult1a1	Nav1	Atf7ip	Hsp90aa1	Sec61b	Epsti1	Mertk
63	Plxnc1	Evl	Tap2	Fgf3	Gmfg	Dtx3l	Ckb
64	Rras	Rabgap1l	Sox4	Cmtm7	S100a6	Ms4a4c	Cd74
65	Nt5c3	Il17re	Cd244	Slc22a3	Polr2a	Samhd1	Frmd4b
66	Col4a1	Lax1	Mcm7	Hspe1	Coro1a	Gbp2	Fcgr3
67	Gucy1a1	Tbxa2r	Erp29	Nop10	Rpl24	Cxcl10	Pilrb2
68	Gata2	Mgat1	Asap1	Siva1	Ssr2	Trim25	Unc93b1
69	Slfn2	Ctss	Tnfaip8l2	Rpl28	Ccdc34	Oas2	Fgr
70	Gstm1	Tnip3	Tspo	Ssr4	Gpr146	Stat2	Timp2
71	Cers4	Ccl4	Dapp1	Rps27l	Calm1	Tspo	Pid1
72	Sgms1	Lztfl1	Cebpa	Myc	Rplp0	Trim12a	Fcgr1
73	Obscn	Ikzf1	Gmfg	Actb	Fth1	Parp11	Clec4a2
74	Bex4	Prr5	Dnmt1	Tacstd2	Rnf130	Gbp2b	Zeb2
75	Kcnk5	Phf14	Git2	mt-Nd1	Nucks1	Slfn9	Filip1l
76	Nrk	Basp1	Gpx1	Ybx1	Tspo	BC147527	Actb
77	Ankrd33b	Emp1	Hsp90ab1	Plac8	Tnfaip8l2	Mb21d1	Hebp1
78	Myct1	Scn1b	Adgrg1	Fkbp4	Cox5b	Ifi208	Itgad
79	Pbx1	Cdk19	Clec4e	Dtymk	Capg	Irgm2	Ccl6
80	Bdh2	Gem	Gm2a	Nolc1	Gm20342	Tuba1b	Fabp4
81	Gm973	Cmah	Rgs10	Ydjc	Cks2	Psme2b	Lgals3
82	Slc18a2	Stxbp4	Kcnq1ot1	Olfr417	Ccl9	Serpina3f	Cd300a
83	Ccdc112	Bmyc	Snx14	Lgals9	Hscb	Tgtp2	Lyz2
84	Ifitm2	Shisa5	Fam133b	Tkt	Hist1h1d	H2-T23	Serpinb6a
85	Fgd5	Foxp1	Gm26917	Gapdh	Calr	Ifitm1	Plxnb2
86	Hoxb2	Thbs1	Plppr3	Gata2	Nasp	Gbp6	Fcgrt
87	Unc45b	Pan3	Slc22a3	Rpl4	Tmem14c	Helz2	Lcp2
88	Cavin1	Lims1	Chdh	Pa2g4	Ccnd3	Fam241a	AB124611
89	Prex2	Bcl11a	Kit	Cox6b2	Mgea5	Ifi27l2a	Pld4
90	Serpinf1	Xist	Sgk3	Cpa3	Rnaseh2c	H2-Q4	Laptm5
91	Slc24a5	Adgrg3	Npm1	Zfpm1	Bex6	Letm2	Ear2
92	Aplp2	Clec2i	Sh3bgrl3	Cct8	Acsl5	Trim30c	Irf8
93	Lst1	Rcsd1	Ncl	Rfc2	Hsp90b1	Socs1	Aplp2
94	Hdgfl3	Sh3bgrl3	Tkt	Tipin	Rpl4	Sp110	Blvrb
95	Ctnnal1	Rhob	Parp8	Ifitm2	Tgfb1	Tnfsf10	Col14a1
96	Scarf1	Rgs2	Dach1	Gnl3	1500009L16Rik	Tgtp1	Cd81
97	Fam110c	Arhgdib	Macf1	S100a10	Rps27l	Camk2d	Slc11a1
98	Vwf	Rhoh	Wsb1	Serpina3g	Slc9a3r1	Ifi207	Pilrb1
99	Slamf1	Slc18a1	Fabp5	Rack1	Prdx4	Phf11d	Itm2c
100	Ecscr	Tmem108	Xist	H1fx	Rpl6	Uba7	Vsir

Table S5, related to Figure 4. Overexpressed DNA repair genes in *Aldh2$^{-/-}$Adh5$^{-/-}$* progenitors following compensation for cell cycle phase.

Cell cycle phase was determined (as per Figure S3E) for each single cell transcriptome. Comparison of cells at equivalent cell cycle phase between WT and *Aldh2$^{-/-}$Adh5$^{-/-}$* was performed to generate significantly overexpressed genes in GO term GO:0006281 (DNA repair) (fold change > 1.2 and adjusted *p* value *p*<0.1).

Gene	adjusted p value	log2 fold change
Pclaf	1.7921E-105	0.765312
Dntt	3.37977E-67	1.801023
Rfc2	1.33044E-41	0.286725
Xrn2	1.2133E-32	0.269335
Ier3	5.55277E-27	0.748824
Xpc	6.33631E-25	0.481973
Pold4	7.64881E-24	0.360293
Mcm3	8.23846E-23	0.300839
Rfc5	1.44011E-15	0.346084
4930447C04Rik	4.64894E-14	1.266671
Clspn	8.56797E-14	0.450605
Chaf1b	9.3004E-14	0.397027
Neil3	2.52424E-12	0.633323
Nsd2	6.02353E-10	0.327927
Hmga2	1.15329E-09	0.513935
Xrcc6	6.55307E-08	0.519498
Paxx	3.31423E-07	0.358693
Xpa	1.36276E-06	0.355939
Msh5	4.61548E-06	0.821602
Gins2	6.94867E-06	0.263547
Poll	9.72739E-06	0.558407
Rmi2	1.89807E-05	0.400336
Eya1	0.000121298	0.445072
Nudt1	0.00034174	0.274727
Parpbp	0.00074051	0.283223
Fancd2	0.001577611	0.339103
Spo11	0.001733193	1.360291
Pnkp	0.001894512	0.319521
Nthl1	0.002547337	0.576525
Esco2	0.002569652	0.284777
Rad51	0.002666466	0.34912
Rbbp8	0.003405985	0.292151
Mrnip	0.006031151	0.504284
Rnf169	0.01175469	0.391834
Polk	0.01650161	0.322304
Parp3	0.02550269	0.386784
Brca2	0.02641574	0.270288
Rad18	0.05740908	0.291865
Zfp365	0.06057999	1.358286
Mcm8	0.07663427	0.349946
Neil1	0.07741061	0.404398
Eme1	0.09406375	0.485893
Rad51c	0.09528128	0.555988

Table S6, related to Table 1. Allele frequency of ADH5 variants detected in seven cases with FA-like BMF syndrome

Genomic location (hg19)	Reference allele /Alternative allele	cDNA	Protein	Allele frequency (allele count/allele number) in HERPACC-2	Allele frequency (allele count/allele number) in gnomAD
chr4: 99997854	C/T	c.564+1G>A	p.L188PfsX4	not detected	not detected
chr4: 99996194	C/G	c.832G>C	p.A278P	0.0008 (7/8412)	0.0002 (4/17696 in East Asia)
chr4: 99996094	C/T	c.932G>A	p.G311D	not tested	not detected
chr4: 99993857	TC/T	c.966delG	p.W322X	0.0005 (4/8412)	0.0005 (9/17604 in East Asia)

*gnomAD database is available at http://gnomad.broadinstitute.org.
HERPACC-2, Hospital-based Epidemiologic Research Program at Aichi Cancer Center; gnomAD, Genome Aggregation Database

Table S7, related to STAR methods. Primer sequences in this study.

Cloning Primers for *mmAldh2*

cDNA	mmAldh2_cl_fwd	TTATATGCTAGCTCAGCCGCCGCCACCAGCGCGGTG
	mmAldh2_cl_rev	GATGGCGGATCCAAGCTTGCATGATTCTTACGAGTTCTTCTG TGGCACTT

Sequencing Primers for *ADH5* gene

Exon2 (containing initiating codon)	KD16-219	ATTAATCTGTATCTAAATTGGCTATTAAGT
	KD15-304	TCTACTCATCTATCCAGAGACCTCATTGTG
Exon3	KD16-220	CTTGTATCTGTACCTCTGAATTGCATGCAC
	KD16-221	TTAGATGATACCTATTCATAAATAGTGGGT
Exon4	KD16-222	ATCCGTTGACAGGAATTTGCAAGGGCTGAT
	KD16-223	GGTTAATGACCTAAATTATTAAATAATTC
Exon5 (c.564+1G>A)	KD15-315	AGTTGTGGCTGATATCTCTGTTGCTAAAAT
	KD15-316	ACTCATTCTACCAGAGTCAAGAGAATCACT
Exon6	KD16-224	AATATTTACTGGTCATTATTTTTAAAACAT
	KD16-225	TTTAATCTAAAACTGCACTTC
Exon7 (c.G832>C; c.G932>A)	KD15-357	AACTATGCCATCTCTAGAAGTTTCGCCAGC
	KD16-226	TAGGTGGCTGGGATTAAACATCTGCCAATG
Exon8 (c.966delG)	KD15-319	ATTTACTTCTTCACTGAAAGGTGTTGGTCA
	KD15-320	TTTGGACATATATTCAGACACCAACTTTGG
Exon9	KD15-323	AGAATGTAATAATGATGTTGAGTTTGAGGG
	KD15-324	AAGCTCTACGAGGCTGTGAGGTTGGAGGCG

Sequencing Primers for *ALDH2*2*

c.G1510>A	KD18-12	CAGGGATCCTGGCACATACTTGTTATCTTA
	KD18-13	ACGGATCCTGGTGAAAATCTGAAAAGATTC

Primers for RT-PCR

ADH5 (full-length)	KD16-155	CACCATGGCGAACGAGGTTATCAAGTGCA
	KD16-156	TTAAATCTTTACAACAGTTCGAATG
ADH5 (Exon4-Exon6)	KD16-199	CACTGTCATCCCACTTTACATCCCA
	KD16-200	TGCCAATCCGACTCCTCCCAGACA
GAPDH	KD12-215	GTCTCCTCTGACTTCAACAGCG
	KD12-216	ACCACCCTGTTGCTGTAGCCAA

***ADH5* site-directed mutagenesis**

c.G932>A	KD16-574	TGGTAACAGATCGCACATGGAAAGGCACTG
	KD16-565	CCATGTGCGATCTGTTACCAGCTGGAATGG
c.G832>C	KD16-239	GAGAGCACCACTTGAGGCATGTCACAAGGGCTGGGGCGTC
	KD16-240	TCAAGTGGTGCTCTCATGACCTTCACATTACCAATACATT
