# Supplementary material for: Two Aldehyde Clearance Systems Are Essential to Prevent Lethal Formaldehyde Accumulation in Mice and Humans
Source: Mol Cell. 2020 Dec 17;80(6):996–1012.e9. doi: 10.1016/j.molcel.2020.10.012 (PMC7758861; doi:10.1016/j.molcel.2020.10.012)
Supplement: Document S2. Article plus Supplemental Information [file mmc2.pdf]

Molecular Cell

Article

Two Aldehyde Clearance Systems Are Essential to Prevent Lethal Formaldehyde Accumulation in Mice and Humans

Graphical Abstract

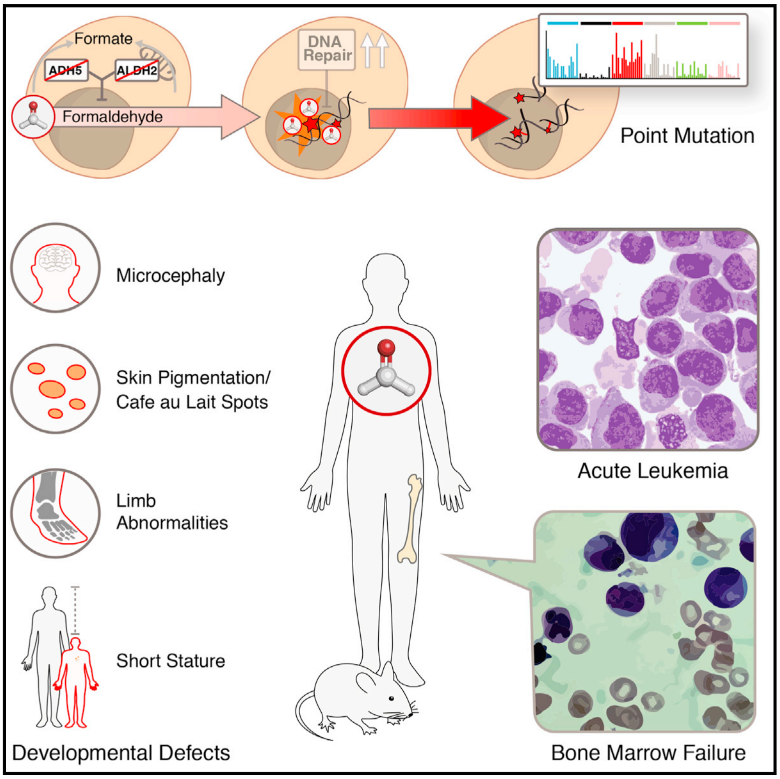

Highlights

- Toxic levels of genotoxic formaldehyde are produced endogenously in mammals
- Two enzymes, ADH5 and ALDH2, are critical for clearance of endogenous formaldehyde
- Their loss in mice and humans causes defective hematopoiesis and increased cancer
- Elevated formaldehyde causes DNA damage and mutation signature found in many cancers

Authors

Felix A. Dingler, Meng Wang, Anfeng Mu, ..., Michael R.G. Hodskinson, Minoru Takata, Ketan J. Patel

Correspondence

takata.minoru.8s@kyoto-u.ac.jp (M.T.), kjp@mrc-lmb.cam.ac.uk (K.J.P.)

In Brief

Dingler et al. show that formaldehyde is produced endogenously at sufficient levels to induce and overwhelm DNA repair. Two enzymes, ADH5 and ALDH2, are critical in clearance of formaldehyde, whose loss results in a bone marrow failure and leukemia syndrome of purely metabolic origin.

Dingler et al., 2020, Molecular Cell *80*, 996–1012
December 17, 2020 © 2020 MRC Laboratory of Molecular Biology.
Published by Elsevier Inc.
https://doi.org/10.1016/j.molcel.2020.10.012

CellPress

CellPress
OPEN ACCESS

Molecular Cell

Article

Two Aldehyde Clearance Systems Are Essential to Prevent Lethal Formaldehyde Accumulation in Mice and Humans

Felix A. Dingler,[1,25] Meng Wang,[1,2,25] Anfeng Mu,[3,4,5,25] Christopher L. Millington,[1] Nina Oberbeck,[1] Sam Watcham,[2,6] Lucas B. Pontel,[1,7] Ashley N. Kamimae-Lanning,[1] Frederic Langevin,[1] Camille Nadler,[1] Rebecca L. Cordell,[8] Paul S. Monks,[8] Rui Yu,[9] Nicola K. Wilson,[2,6] Asuka Hira,[3,4] Kenichi Yoshida,[10] Minako Mori,[3,4,11] Yusuke Okamoto,[3,4,11] Yusuke Okuno,[12] Hideki Muramatsu,[12] Yuichi Shiraishi,[13] Masayuki Kobayashi,[11,14] Toshinori Moriguchi,[14] Tomoo Osumi,[15] Motohiro Kato,[15] Satoru Miyano,[16] Etsuro Ito,[17] Seiji Kojima,[12] Hiromasa Yabe,[18] Miharu Yabe,[18] Keitaro Matsuo,[19,20] Seishi Ogawa,[10,21,22] Berthold Göttgens,[2,6] Michael R.G. Hodskinson,[1] Minoru Takata,[3,4,*] and Ketan J. Patel[1,23,24,26,*]
[1]MRC Laboratory of Molecular Biology, Francis Crick Avenue, Cambridge CB2 0QH, UK
[2]Department of Haematology, University of Cambridge, Cambridge, UK
[3]Laboratory of DNA Damage Signaling, Department of Late Effects Studies, Radiation Biology Center, Kyoto University, Kyoto, Japan
[4]Department of Genome Biology, Graduate School of Biostudies, Kyoto University, Kyoto, Japan
[5]Department of Clinical Application, Center for iPS Cell Research and Application, Kyoto University, Kyoto, Japan
[6]Wellcome-MRC Cambridge Stem Cell Institute, Jeffrey Cheah Biomedical Centre, University of Cambridge, Cambridge, UK
[7]Instituto de Investigación en Biomedicina de Buenos Aires (IBioBA)-CONICET, Polo Científico Tecnológico, Godoy Cruz 2390, C1425FQD Buenos Aires, Argentina
[8]Department of Chemistry, University of Leicester, Leicester LE1 7RH, UK
[9]Department of Environmental Sciences and Engineering, Gillings School of Global Public Health, University of North Carolina, Chapel Hill, NC 27599, USA
[10]Department of Pathology and Tumor Biology, Graduate School of Medicine, Kyoto University, Kyoto, Japan
[11]Department of Hematology and Oncology, Graduate School of Medicine, Kyoto University, Kyoto, Japan
[12]Department of Pediatrics, Nagoya University Graduate School of Medicine, Nagoya, Japan
[13]Section of Genome Analysis Platform, Center for Cancer Genomic and Advanced Therapeutics, National Cancer Center, Tokyo, Japan
[14]Department of Hematology, Kyoto Katsura Hospital, Kyoto, Japan
[15]Children's Cancer Center, National Center for Child Health and Development, Tokyo, Japan
[16]Laboratory of DNA Information Analysis, Human Genome Center, The Institute of Medical Science, University of Tokyo, Tokyo Japan
[17]Department of Pediatrics, Hirosaki University Graduate School of Medicine, Hirosaki, Japan
[18]Department of Innovative Medical Science, Tokai University School of Medicine, Isehara, Japan
[19]Division of Cancer Epidemiology and Prevention, Aichi Cancer Center Research Institute, Nagoya, Japan
[20]Division of Analytical Cancer Epidemiology, Nagoya University Graduate School of Medicine, Nagoya, Japan
[21]Department of Medicine, Center for Hematology and Regenerative Medicine, Karolinska Institute, Sweden
[22]Institute for the Advanced Study of Human Biology (WPI-ASHBi), Kyoto University, Kyoto, Japan
[23]Department of Medicine, University of Cambridge, Addenbrooke's Hospital, Cambridge CB2 2QQ, UK
[24]MRC Weatherall Institute of Molecular Medicine, University of Oxford, John Radcliffe Hospital, Oxford OX3 9DS, UK
[25]These authors contributed equally
[26]Lead Contact
*Correspondence: takata.minoru.8s@kyoto-u.ac.jp (M.T.), kjp@mrc-lmb.cam.ac.uk (K.J.P.)
https://doi.org/10.1016/j.molcel.2020.10.012

SUMMARY

Reactive aldehydes arise as by-products of metabolism and are normally cleared by multiple families of enzymes. We find that mice lacking two aldehyde detoxifying enzymes, mitochondrial ALDH2 and cytoplasmic ADH5, have greatly shortened lifespans and develop leukemia. Hematopoiesis is disrupted profoundly, with a reduction of hematopoietic stem cells and common lymphoid progenitors causing a severely depleted acquired immune system. We show that formaldehyde is a common substrate of ALDH2 and ADH5 and establish methods to quantify elevated blood formaldehyde and formaldehyde-DNA adducts in tissues. Bone-marrow-derived progenitors actively engage DNA repair but also imprint a formaldehyde-driven mutation signature similar to aging-associated human cancer mutation signatures. Furthermore, we identify analogous genetic defects in children causing a previously uncharacterized inherited bone marrow failure and pre-leukemic syndrome. Endogenous formaldehyde clearance alone is therefore critical for hematopoiesis and in limiting mutagenesis in somatic tissues.

 © 2020 MRC Laboratory of Molecular Biology. Published by Elsevier Inc.
This is an open access article under the CC BY license (http://creativecommons.org/licenses/by/4.0/).

INTRODUCTION

Reactive chemistry drives many fundamental metabolic processes of life. However, the reactive metabolites involved are often toxic because they can inappropriately attack cellular constituents, ultimately driving degenerative changes associated with aging and carcinogenesis. The best-studied group of such molecules are reactive oxygen species (ROS), which have been implicated in a wide range of (patho)physiological processes. A new and emerging group of reactive metabolites are endogenous aldehydes, and the threat they pose, combined with their molecular diversity, could explain why we possess so many enzymes to detoxify them. There are at least 19 distinct aldehyde dehydrogenases (ALDHs) as well as a number of enzymes that process aldehyde-glutathione conjugates (glutathione S-transferases [GSTs] and ADH5) (Jackson et al., 2011). However, we understand very little about the physiological importance of different aldehydes, which enzymes metabolize them, and whether these detoxifying enzymes are functionally linked to one another.

ALDH2 is a mitochondrial ALDH that utilizes NAD^+ as a cofactor to oxidize acetaldehyde to acetate, which is then utilized in the Krebs cycle (Jacobson and Bernofsky, 1974). ALDH2 is important in ethanol metabolism, and deficiency of this enzyme is very common in humans, caused by a dominant-negative mutation in the *ALDH2* gene (*ALDH2*2)* that destabilizes the tetrameric enzyme, resulting in a red flushing reaction upon alcohol consumption because of buildup of acetaldehyde (Harada et al., 1981). In contrast to ALDH2, ADH5 is a cytosolic enzyme that does not act on free aldehydes but oxidizes the spontaneously formed glutathione (GSH) conjugate of formaldehyde to formate, which can be used in one-carbon metabolism. Formaldehyde likely originates from a variety of cellular sources, such as histone demethylation and folic acid decomposition (Burgos-Barragan et al., 2017; Uotila and Koivusalo, 1974). These two examples illustrate how aldehyde-processing enzymes convert the two simplest aldehydes into molecules useful for essential metabolism.

Recent research has established that aldehyde clearance constitutes just the first tier of protection against these molecules. If this is genetically ablated, as in *Aldh2*$^{-/-}$ or *Adh5*$^{-/-}$ mice, then DNA crosslink repair by Fanconi anemia (FA) genes provides an essential backup. Thus, the aldehydes detoxified by these respective enzymes are genotoxic and, in the case of acetaldehyde, cause DNA interstrand crosslinks (Hodskinson et al., 2020). This is why, when ALDH2 or ADH5 deficiency is combined with loss of the crosslink repair gene *Fancd2*, mice rapidly develop hematopoietic failure and acute leukemia (Garaycoechea et al., 2012, 2018; Langevin et al., 2011; Pontel et al., 2015). These findings lead to the proposal that aldehydes may be metabolic drivers for the human genetic disease FA, where patients lack the DNA repair pathway that provides the second tier of protection. An additional intriguing observation is that exposing cells to formaldehyde destabilizes the BRCA2 protein, the genotoxin itself causing DNA repair deficiency and genomic instability (Tan et al., 2017). This is postulated to explain why women haploinsufficient for BRCA2 might be predisposed to breast cancer without loss of their functional BRCA2 allele. What is absent in these studies is evidence to directly define which aldehyde(s) are driving these effects because of the lack of reliable methods to identify and quantify aldehydes in organisms. In this study, we discover that endogenous formaldehyde is the main physiological substrate for ALDH2 and ADH5. We define the severe hematopoietic consequences and explain what happens when this clearance fails in mice and humans.

RESULTS

Genetic Redundancy between *Aldh2* and *Adh5* in Mice

We first set out to determine the expression profile of the many aldehyde-detoxifying enzymes across hematopoietic lineages. Single-cell RNA sequencing (scRNA-seq) of primitive murine bone marrow cells shows that expression of two genes, *Aldh2* and *Adh5*, stands out as being widespread across hematopoietic progenitors (Figure 1A). To find out whether these two enzymes functionally interact with each other, we crossed *Aldh2*$^{-/-}$ with *Adh5*$^{-/-}$ mice to obtain *Aldh2*$^{-/-}$*Adh5*$^{-/-}$ mice. Although indistinguishable at birth (Figure S1A), their growth is severely compromised, and most die in the perinatal window without an obvious cause of death. Importantly, a small proportion of *Aldh2*$^{-/-}$*Adh5*$^{-/-}$ mice survive into adulthood; these animals are significantly growth retarded, small, and lean (Figures 1B–1D, S1A, and S1B). Aged *Aldh2*$^{-/-}$*Adh5*$^{-/-}$ mice continued to remain considerably smaller than wild-type littermate controls, and none lived longer than 47 weeks. This is due to a general decline in condition and predisposition to cancer, including thymic T cell leukemia (Figure 1B and S1C–S1E; Table S1). Furthermore, *Aldh2*$^{-/-}$*Adh5*$^{-/-}$ mice are mildly anemic with macrocytosis (increased red cell mean corpuscular volume) and have depressed white blood cell counts, predominantly in the lymphocyte fraction (Figure 1E). In summary, combined inactivation of the aldehyde-clearing enzymes ALDH2 and ADH5 leads to perinatal lethality, growth failure, lymphopenia, and lymphoid malignancies.

Deficiency in *Aldh2* and *Adh5* Disrupts Hemato-lymphoid Development

The reduced blood counts in *Aldh2*$^{-/-}$*Adh5*$^{-/-}$ mice prompted us to carry out a detailed analysis of blood production. Flow cytometry analysis of the bone marrow indicates a reduced proportion of LKS (Lineage$^-$ [Lin$^-$] c-Kit$^+$ Sca-1$^+$) cells, representing hematopoietic stem cells (HSCs) and progenitors in which we observe reduced long-term HSCs (LT-HSCs; Lin$^-$ c-Kit$^+$ Sca-1$^+$ Flt3$^-$ CD34$^-$ cells; Figure 2A) and multipotent progenitor cells (MPPs; Lin$^-$ c-Kit$^+$ Sca-1$^+$ Flt3$^+$ CD34$^+$ cells; Figure S2A). Among more differentiated progenitors, we find reduced common lymphoid progenitors (CLPs, Lin$^-$ c-Kit^{lo} Sca-1$^+$ Flt3$^+$ interleukin-7Rα [IL-7Rα]$^+$ cells). Although the common myeloid progenitor (CMP) population (Lin$^-$ c-Kit$^+$ Sca-1$^-$ CD34$^+$ CD16/32^{lo} cells) is mildly reduced, it is proportionately less affected so that the relative myeloid contribution in bone marrow and blood is increased significantly (Figure 2B; Figure S2A). To functionally validate the hematopoietic defect in *Aldh2*$^{-/-}$*Adh5*$^{-/-}$, we transplanted its bone marrow cells (CD45.2$^+$) with wild-type competitor-derived cells (CD45.1$^+$) into lethally irradiated recipients (CD45.1$^+$ CD45.2$^+$). Over a period of 4 months, *Aldh2*$^{-/-}$

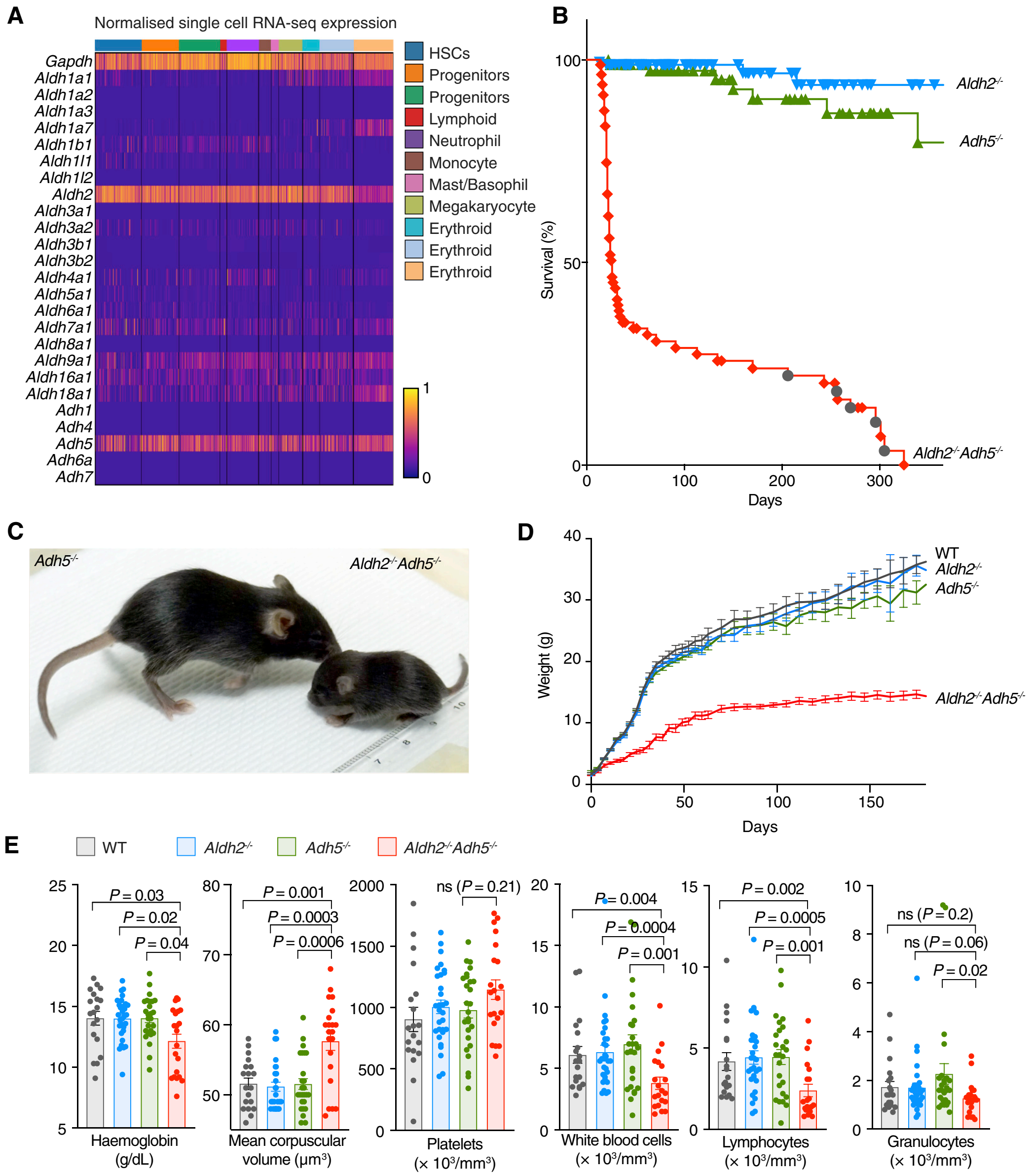

Figure 1. Postnatal Lethality, Stunted Growth, and Cancer Predisposition in *Aldh2*$^{-/-}$*Adh5*$^{-/-}$ Mice

(A) Gene expression analysis of *Aldh* and *Adh* gene families by scRNA-seq in WT bone marrow progenitor cells (Lin$^-$ c-Kit$^+$ Sca-1$^+$). The colored bar at the top represents the assigned lineage of cell transcriptomes.

(B) Kaplan-Meier survival curve of *Aldh2*$^{-/-}$, *Adh5*$^{-/-}$, and *Aldh2*$^{-/-}$*Adh5*$^{-/-}$ mice (n = 166, 89, 67). Dark gray circles indicate cancer deaths.

(C) Photograph of *Aldh2*$^{-/-}$*Adh5*$^{-/-}$ mouse (right) and its littermate *Adh5*$^{-/-}$ control (left).

(D) Total body mass as mean ± SEM of WT, *Aldh2*$^{-/-}$, *Adh5*$^{-/-}$, and *Aldh2*$^{-/-}$*Adh5*$^{-/-}$ mice (initial n = 35, 58, 60, 16).

(E) Blood parameters in *Aldh2*$^{-/-}$*Adh5*$^{-/-}$ mice with controls (mean ± SEM; n = 21, 30, 26, 19, left to right).

The p values were determined by two-tailed Mann-Whitney *U* test. See also Figure S1 and Table S1.

Adh5$^{-/-}$-transplanted bone marrow gave lower reconstitution across B220$^+$ (B cells), CD4$^+$/CD8$^+$ (T cells), and Gr-1$^+$/Mac-1$^+$ (myeloid cells) in the blood, with the contribution to lymphoid lineages decreasing over time. Correspondingly, we also observed a reduced contribution to LT-HSC, LKS, and CLP compartments at 4 months (Figures S2B–S2D).

To further characterize hematopoiesis in *Aldh2*$^{-/-}$*Adh5*$^{-/-}$ mice, we applied droplet-based scRNA-seq to LK (Lin$^-$ c-Kit$^+$) and Lin$^-$ c-Kit^{lo} Sca-1$^+$ cells of *Aldh2*$^{-/-}$*Adh5*$^{-/-}$ mice to profile the heterogeneous stem and progenitor populations in an unbiased fashion (Figures 2C–2F), sampling approximately 8,000 transcriptomes per genotype that were clustered using the Louvain algorithm. Each cluster was assigned a cell identity based on expression of marker genes (Figure S3A; see STAR Methods for details). Strikingly, hematopoietic stem and progenitor cells (HSPCs) and erythroid progenitors in *Aldh2*$^{-/-}$*Adh5*$^{-/-}$ mice

CellPress
OPEN ACCESS

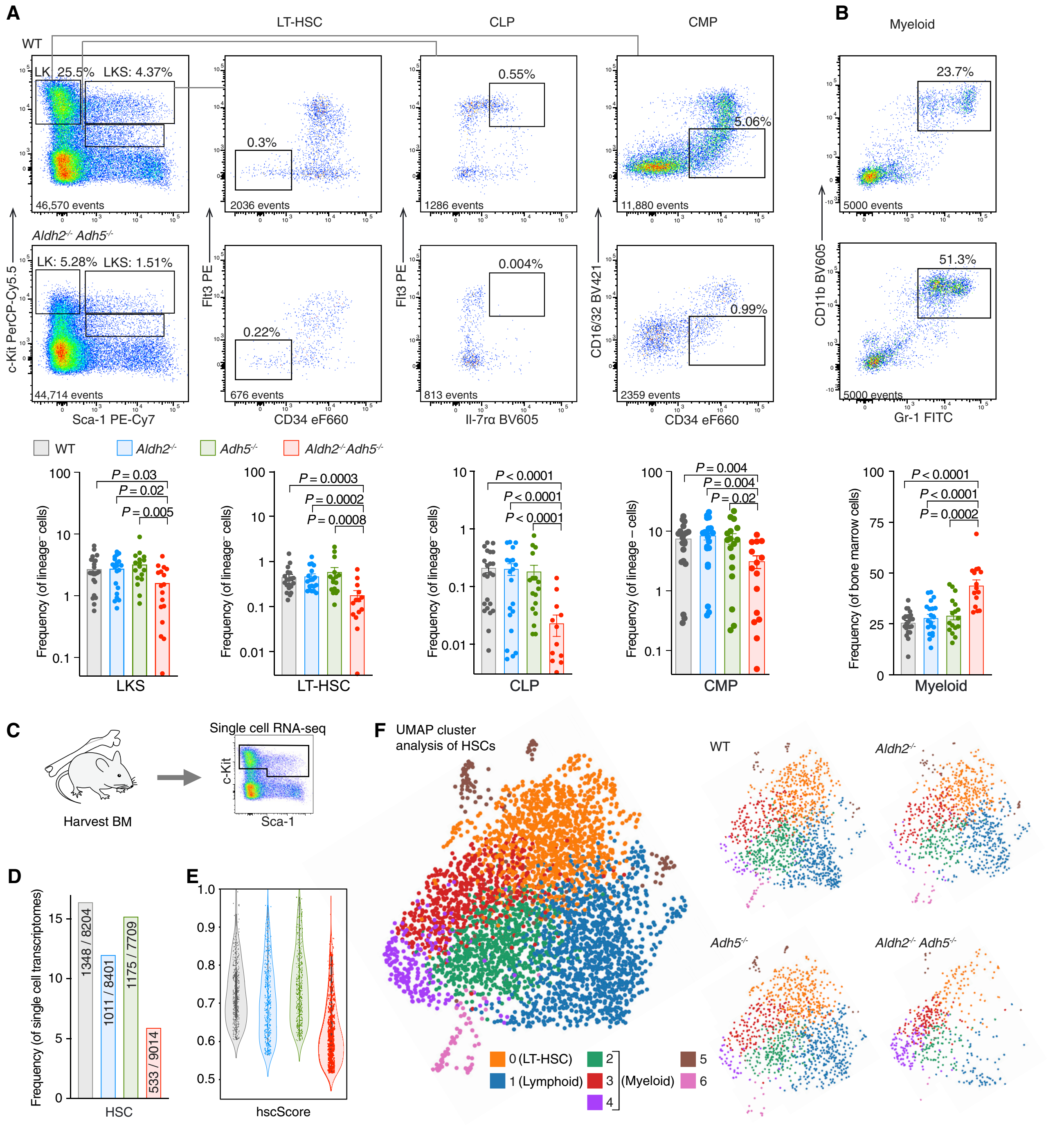

Figure 2. Disrupted Aldehyde Catabolism Compromises Hematopoiesis

(A and B) Representative flow cytometry plots from *Aldh2*$^{-/-}$*Adh5*$^{-/-}$ and WT mice showing bone marrow LK, LKS, LT-HSC, CLP, and CMP (A) and myeloid populations (B). Bottom: quantification of the respective populations assessed by flow cytometry in 2- to 30-week-old *Aldh2*$^{-/-}$*Adh5*$^{-/-}$ mice with age-matched controls (mean ± SEM; n = 24, 20, 17, 17, left to right).

(C) scRNA-seq analysis of HSPCs from a 6-week-old female *Aldh2*$^{-/-}$*Adh5*$^{-/-}$ mouse with age- and sex-matched controls.

(D) Fraction of single-cell transcriptomes assigned to the HSC cell identity (numerator) from total transcriptomes analyzed (denominator).

(E) hscScore analysis of single-cell transcriptomes identified as HSCs.

(F) UMAP visualization of HSC transcriptomes colored by cluster. On the left, all 4 genotypes are superimposed; on the right, individual genotypes are shown separately to highlight variation in distribution between the clusters.

The p values were determined by two-tailed Mann-Whitney *U* test. See also Figures S2 and S3 and Tables S2 and S5.

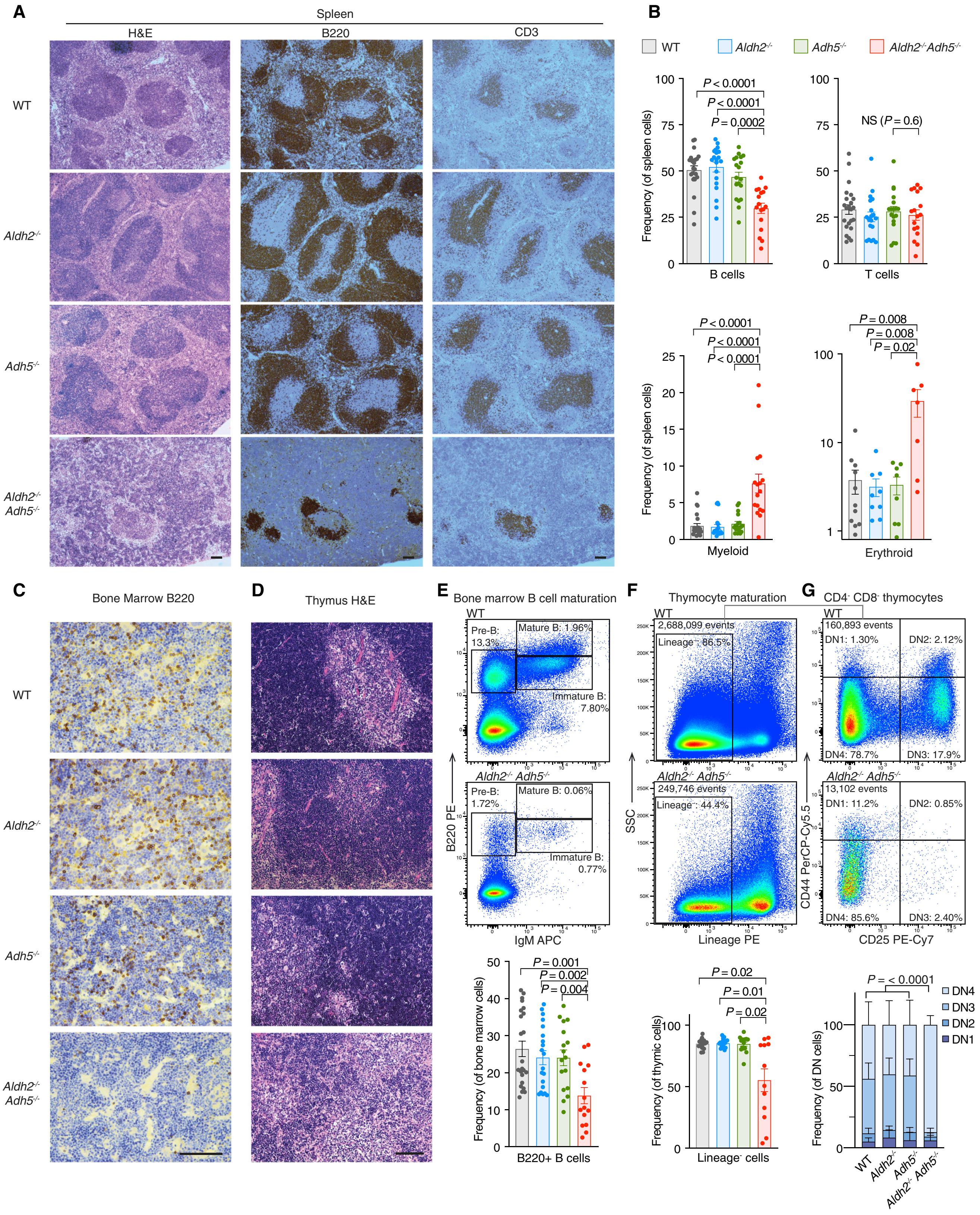

(legend on next page)

showed the greatest transcriptional change from controls (Figures S3B and S3D). Analysis of differentially expressed genes in *Aldh2*$^{-/-}$*Adh5*$^{-/-}$ erythroid progenitors indicates more cells in the S and G2/M cell cycle phase as well as enrichment of genes regulating apoptosis (Figures S3E and S3F; Table S2). Focusing on the HSPCs, these are significantly under-represented in *Aldh2*$^{-/-}$*Adh5*$^{-/-}$ bone marrow (Figure 2D) and rank lower in their hscScore (Hamey and Göttgens, 2019), a metric quantifying transcriptional similarity to reconstituting LT-HSCs (Figure 2E). To assess how the HSC population in *Aldh2*$^{-/-}$ *Adh5*$^{-/-}$ differed from controls, we submitted the differentially expressed genes to Gene Ontology (GO) term enrichment analysis to find multiple gene sets involved in lineage differentiation (Table S3). We proceeded to explore whether HSCs were lineage biased by fine clustering of this population. This generated 7 clusters (0–6), with cluster 0 containing the transcriptional signature associated with LT-HSCs (such as high *Procr* and *Mecom* expression), cluster 1 harboring lymphoid signature genes (such as *Dntt* [*Tdt*]), whereas clusters 2, 3, and 4 contain myeloid signature genes (such as *Mpo*) (for a full list of genes, see Table S4). Intriguingly, HSCs from *Aldh2*$^{-/-}$*Adh5*$^{-/-}$ mice are mostly represented in clusters 3 and 4 (myeloid) and under-represented in clusters 0 (LT-HSCs) and 1 (lymphoid) (Figure 2F). In summary, scRNA-seq analysis of *Aldh2*$^{-/-}$*Adh5*$^{-/-}$ bone marrow reveals a decreased frequency and qualitative score of HSCs with preferential loss of cells with LT-HSC and lymphoid profiles, in agreement with the characterization by cell-surface markers and transplantation experiments.

Although a CLP defect could account for reduced circulating lymphocytes, we wanted to assess for defects in T and B cell maturation in surviving *Aldh2*$^{-/-}$*Adh5*$^{-/-}$ mice. Immunohistological analysis of the *Aldh2*$^{-/-}$*Adh5*$^{-/-}$ spleens showed a gross disruption of lymphoid follicle architecture (Figure 3A), which is less numerous and depleted in B cells (Figure 3B). Indeed, we find a profound defect in B cell development with a reduction in total B220^{+} B cells in the bone marrow (Figures 3C and 3E). Attempts to narrow down the loss of B cells to a specific developmental stage revealed heterogeneity, with some animals exhibiting the strongest defect in the early pre-B cell (B220^{+} immunoglobulin M [IgM]$^{-}$) population, whereas others had a near-normal proportions of pre-B cells in the bone marrow but were profoundly deficient in immature (B220^{+} IgM^{+}) and mature (B220^{+} IgM^{hi}) B cells (Figure S2E). The spleen also showed increased myeloid (CD11b^{+} Gr-1^{+}) and erythroid (Ter-119^{+}) cells (Figure 3B), likely representing stress-induced extramedullary hematopoiesis in response to insufficient blood production.

Despite normal T cell numbers in the peripheral blood and spleen (Figures 3B and S2A), thymi of *Aldh2*$^{-/-}$*Adh5*$^{-/-}$ mice revealed marked atrophy and loss of cellularity (Figures 3D, 3F, and S2F). Thymocyte maturation was also perturbed, with specific loss of cells at the double-negative (DN, Lin^{-} CD4^{-} CD8^{-}) DN2 (CD44^{+} CD25^{+}) and DN3 (CD44^{-} CD25^{+}) stages of thymic development (Figure 3G). These thymic pathologies were most consistently observed in older (>30 weeks old) animals, whereas younger animals (<10 weeks old) exhibited considerable heterogeneity; some animals were enriched for earlier stages of thymic development (DN1–DN3), whereas others lacked early DN cells and were predominantly enriched for DN4 like the older mice (Figure S2G). Analysis of the competitive repopulation experiment showed a particularly low contribution of the *Aldh2*$^{-/-}$ *Adh5*$^{-/-}$ donor to the DN compartment (Figure S2H); recapitulating the heterogeneity in DN stages, about half of the recipients showed a strong DN1 bias (Figure S2I). In conclusion, accumulation of aldehyde(s) in *Aldh2*$^{-/-}$*Adh5*$^{-/-}$ mice impairs hematopoiesis in several respects: early blood progenitors such as LT-HSCs and CLPs are depleted, but more striking defects are seen in the more committed cell populations of T and B cells, for which impaired maturation ultimately manifests in disordered secondary lymphoid structures.

Induction of DNA Repair in *Aldh2*$^{-/-}$*Adh5*$^{-/-}$ Mice and Consequences of Formaldehyde Challenge in *Adh5*$^{-/-}$ Mice

Aldehyde(s) detoxified by ALDH2 or ADH5 are genotoxic, and the DNA damage they cause necessitates crosslink repair (Langevin et al., 2011; Pontel et al., 2015). However, in *Aldh2*$^{-/-}$ *Adh5*$^{-/-}$ mice, DNA repair is genetically intact, so it is important to address whether these animals show any evidence of DNA damage and engagement of DNA repair. In the first instance, we investigated genome instability in the hematopoietic compartment of *Aldh2*$^{-/-}$*Adh5*$^{-/-}$ mice. During the course of maturation, red blood cells (RBCs) extrude and lose their nucleus; however, broken chromosomes can partition into micronuclei that can persist in enucleated RBCs (Figure 4A; Bryce et al., 2007). Peripheral blood from single-mutant *Aldh2*$^{-/-}$ and *Adh5*$^{-/-}$ mice shows no strong increase in micronuclei over their wild-type controls. However, *Aldh2*$^{-/-}$*Adh5*$^{-/-}$ RBCs contained increased numbers of micronucleated cells, indicating genome instability and chromosome breakage in these mice (Figure 4B). Next, we set out to assess the DNA repair response by quantifying sister chromatid exchanges (SCEs) in bone marrow using a protocol that assesses these events *in vivo* (Giri and Chatterjee,

Figure 3. Aldehyde Catabolism Is Essential for Lymphoid Development

(A) Spleen histology (hematoxylin and eosin [H&E stain]) and immunohistochemistry for B220 or CD3.

(B) Quantification of splenic B, T, myeloid, and erythroid precursors assessed by flow cytometry (n = 23, 20, 19, 17, left to right).

(C) Bone marrow immunohistochemistry for B220.

(D) Thymus histology (H&E stain).

(E) Representative flow cytometry plots showing bone marrow B cell development and quantification of total B220^{+} cells (mean ± SEM; n = 23, 20, 19, 15, left to right).

(F and G) Representative flow cytometry plots and quantification of the thymic Lin^{-} population (F) and Lin^{-} CD4^{-} CD8^{-} (DN) populations defined by CD44 and CD25 expression (G). Mice analyzed for thymic Lin^{-} populations were 2–30 weeks old (n = 23, 20, 19, 15, left to right). Mice analyzed for thymic DN populations were older than 30 weeks (n = 7, 7, 5, 5 mice, left to right).

All bar graphs are shown with mean ± SEM. The p values were determined by two-tailed Mann-Whitney *U* test except for (G), where pairwise χ^2 tests of average distributions were performed. Scale bars indicate 100 μm. See also Figures S2 and S3.

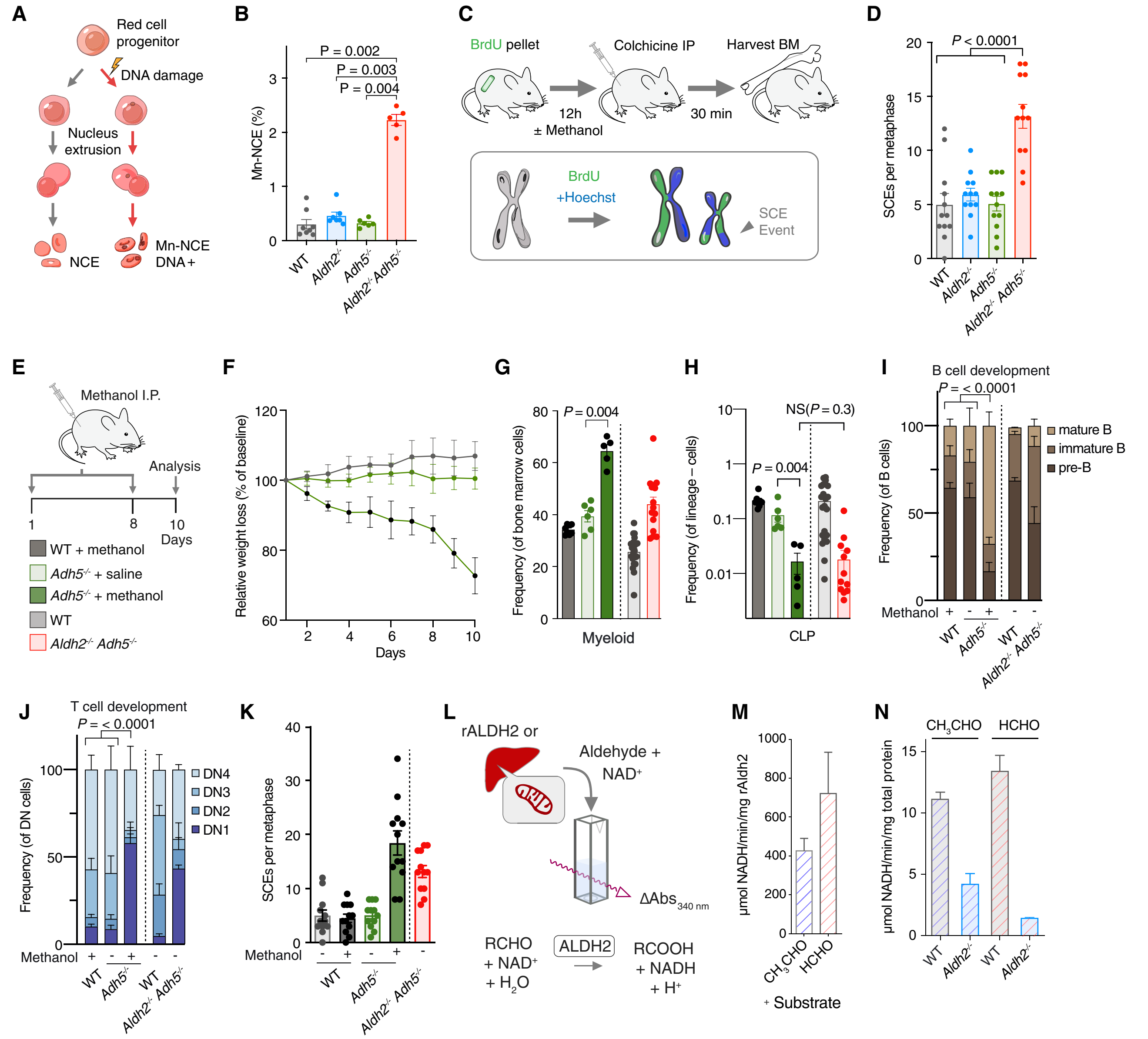

Figure 4. DNA Damage in *Aldh2*$^{-/-}$*Adh5*$^{-/-}$ Mice and Methanol Challenge of *Adh5*$^{-/-}$ Mice Phenocopies the Double Mutant

(A) Scheme of the micronucleus assay.

(B) Micronuclei in *Aldh2*$^{-/-}$*Adh5*$^{-/-}$ mice and controls. (mean ± SEM, n = 8, 7, 6, 5, left to right).

(C) SCE analysis in bone marrow cells.

(D) Quantification of SCE in *Aldh2*$^{-/-}$*Adh5*$^{-/-}$ mice and controls. (mean ± SEM, n = 12 metaphases per group).

(E) Treatment of mice with intraperitoneal methanol injection.

(F) Percentage of weight loss relative to baseline weight on day 0 (mean ± SD, n = 10; WT + methanol, 6; *Adh5*$^{-/-}$ + saline and 6; *Adh5*$^{-/-}$ + methanol).

(G and H) Frequency of bone marrow myeloid (CD11b^{+} Gr-1^{+}) and CLP cells (mean ± SEM, n = 8, 6, 5, left to right).

(I and J), Frequency of bone marrow B cell (pre-B, immature and mature) and thymus DN populations (DN1–DN4) (mean and SEM; n = 8, 6, 5 mice, left to right).

(K) Quantification of SCEs of methanol-treated mice and controls. n = 12 metaphases per group.

(L) ALDH activity assays on recombinant ALDH2 (rALDH2) or mitochondrial extracts from WT or *Aldh2*$^{-/-}$ liver.

(M and N) ALDH activity performed with acetaldehyde (CH_3CHO) and formaldehyde (HCHO) substrates using rALDH2 (M); WT and *Aldh2*$^{-/-}$ liver mitochondrial extract (N). Activity is expressed as micromolar NADH per minute per milligram of total protein (mean and SD; n = 2).

The p values were determined by two-tailed Mann-Whitney *U* test, except for (I) and (J), where pairwise χ^2 tests of average distributions were performed. See also Figures S3 and S4.

1998; Orsburn et al., 2010). An SCE event requires crossover mediated by homologous recombination (HR) and is indicative of active DNA repair (Figure 4C). The mean number of SCE events per metaphase is 5 in the wild type (WT), 6 in *Aldh2*$^{-/-}$, and 5 in *Adh5*$^{-/-}$. In contrast, *Aldh2*$^{-/-}$*Adh5*$^{-/-}$ mice show a more than 2-fold increase to an average of 13 SCE events (Figure 4D). Finally, by interrogating the single-cell transcriptomes of HSPCs, we observe increased expression of DNA repair genes in *Aldh2*$^{-/-}$*Adh5*$^{-/-}$ mice (Figure S3G). Inspection of the most overexpressed DNA repair genes revealed a number of recognized DNA crosslink repair genes, including *Brca1*, *Brca2*, *Fanci*, *Fancd2*, *Brip1*, and *Neil3* (Figures S3H and S3I). Importantly, DNA repair genes remained overrepresented after accounting for cell cycle phase (Table S5), which has been associated with expression of DNA repair genes (Walter et al., 2015). Overall, accumulation of endogenous aldehyde(s) in *Aldh2*$^{-/-}$*Adh5*$^{-/-}$ mice elicits vigorous induction of HR-mediated DNA repair in hematopoietic cells.

The fact that combined inactivation of *Aldh2* and *Adh5* causes such a severe phenotype suggests that they might have redundant aldehyde detoxification functions; many ALDH enzymes have overlapping substrate specificities. ADH5 is the main enzyme that detoxifies formaldehyde; nevertheless, *Adh5*$^{-/-}$ mice are largely normal. This could be because formaldehyde accumulation in these mice is restrained by ALDH2. A simple prediction is that challenging *Adh5*$^{-/-}$ mice with methanol would cause formaldehyde to accumulate (Pontel et al., 2015), which might elicit aspects of the phenotype seen in *Aldh2*$^{-/-}$*Adh5*$^{-/-}$ mice. We therefore challenged WT and *Adh5*$^{-/-}$ mice with intraperitoneal methanol injections (Figure 4E), which results in significant weight loss (Figure 4F) and a marked reduction in the CLP fraction and increased myeloid representation in the bone marrow, as seen in *Aldh2*$^{-/-}$*Adh5*$^{-/-}$ mice (Figures 4G and 4H). In addition, methanol challenge in *Adh5*$^{-/-}$ mice also leads to abnormal B cell development with loss of pre-B cells and defective thymic maturation with loss of DN2 and DN3 thymocytes, the same pattern of thymic defect observed in young *Aldh2*$^{-/-}$*Adh5*$^{-/-}$ mice (Figures 4I and 4J). We next wanted to examine the engagement of DNA repair and found that methanol-challenged *Adh5*$^{-/-}$ mice, but not WT controls, showed a 2-fold induction of SCEs (Figure 4K).

Formaldehyde differs from acetaldehyde, the canonical substrate of ALDH2, by a single methyl group, so we assessed biochemically whether it can be detoxified by ALDH2 (Figure 4L). In the first instance, we expressed and purified recombinant murine ALDH2 (rALDH2) in *E. coli* (Figure S4) and confirmed that it can indeed metabolize acetaldehyde. We then tested whether rALDH2 could also metabolize formaldehyde, and it is clear that formaldehyde is an equally good substrate (Figure 4M). Wondering whether compensatory overexpression of one enzyme occurs in the absence of the other, we found no such compensation of *Aldh2* or *Adh5* expression in *Adh5*$^{-/-}$ or *Aldh2*$^{-/-}$ HSPCs by scRNA-seq analysis (Figure S3C). Next, we asked whether the formaldehyde-detoxifying activity of ALDH2 could be confirmed in tissues from WT or *Aldh2*$^{-/-}$ mice. To test this, we prepared mitochondrial extracts from livers of WT and *Aldh2*$^{-/-}$ mice. WT extracts have acetaldehyde- and formaldehyde-metabolizing activity, and both are greatly reduced in *Aldh2*$^{-/-}$ mice (Figure 4N). In conclusion, challenge of *Adh5*$^{-/-}$ mice with a formaldehyde precursor recapitulates DNA damage and hematological phenotypes of *Aldh2*$^{-/-}$*Adh5*$^{-/-}$ mice, and the biochemical activity supports the notion of ALDH2 being responsible for formaldehyde detoxification in *Adh5*$^{-/-}$ mice.

Formaldehyde Accumulation in Blood and DNA Imprints a Mutation Signature in Hematopoietic Precursors

ALDH2 and ADH5 metabolize endogenous formaldehyde. Therefore, the drastic phenotype of *Aldh2*$^{-/-}$*Adh5*$^{-/-}$ could be due to accumulation of reactive formaldehyde. To test this, we set out to directly quantify the formaldehyde concentration in mouse blood. There are many published studies using a range of methods that report blood formaldehyde levels of up to 100 μM in mammals (Heck et al., 1985; Luo et al., 2001; Martos and Pawliszyn, 1998), but, to date, we lack a reliably consistent value. This could be because measuring formaldehyde poses certain challenges; it is a volatile and reactive molecule. We therefore established a method to measure blood formaldehyde that is reliable and reproducible (Figure 5A). A blood sample is drawn and immediately processed to serum, spiked with an internal standard, and derivatized (Cancho et al., 2002). Samples are snap frozen and collected alongside a series of standards. Samples are then subjected to gas chromatography-mass spectrometry (GC-MS), and formaldehyde is detected and quantified. Using this method, we found that the mean blood formaldehyde level increased 11-fold in *Aldh2*$^{-/-}$*Adh5*$^{-/-}$ compared with the WT (4 μM in the WT, 9 μM in *Aldh2*$^{-/-}$, 11 μM in *Adh5*$^{-/-}$, and 44 μM *Aldh2*$^{-/-}$*Adh5*$^{-/-}$) (Figure 5B).

We next wanted to assess whether the greatly increased levels of blood formaldehyde correlated with formaldehyde damage on DNA. A major acetaldehyde adduct is N^2-ethylidene-deoxyguanosine (Garcia et al., 2011), and a major formaldehyde adduct is N^2-hydroxymethyl-deoxyguanosine (HOMeG) (Moeller et al., 2011). However, both of these products are unstable and need to be reduced chemically (to N^2-ethyl-deoxyguanosine, N^2-EtG and N^2-methyl-deoxyguanosine, N^2-MeG) to be quantified. We then used ultra-sensitive liquid chromatography-tandem MS (LC-MS/MS) with synthetic chemical standards (Figure S5) to detect and quantify both adducts on DNA obtained from several tissues (Figures 5A and S5). Although the levels of the acetaldehyde adduct show little differences across genotypes (Figure 5C), there was a marked increase in the levels of N^2-MeG in DNA extracted from livers, kidneys, and brains of *Aldh2*$^{-/-}$*Adh5*$^{-/-}$ mice, to approximately 20-fold of WT levels (Figure 5D).

Although HOMeG may not be a mutagenic base adduct per se, it is noteworthy that *Aldh2*$^{-/-}$*Adh5*$^{-/-}$ cells induce a very vigorous DNA repair response, part of which might be error prone. This could leave a distinct mutational imprint in their genome. We thus set out to determine the mutational landscape in bone marrow cells from 40-week-old *Aldh2*$^{-/-}$*Adh5*$^{-/-}$ mice by whole-genome sequencing (Figure 6A). We found that *Aldh2*$^{-/-}$*Adh5*$^{-/-}$ HSPCs contained a 3-fold increase in the number of single-nucleotide substitutions from approximately 100 to approximately 300 per genome, an increase in double-base substitutions from less than 1 to 4 per genome, and a 2-fold increase in insertions and deletions compared with the

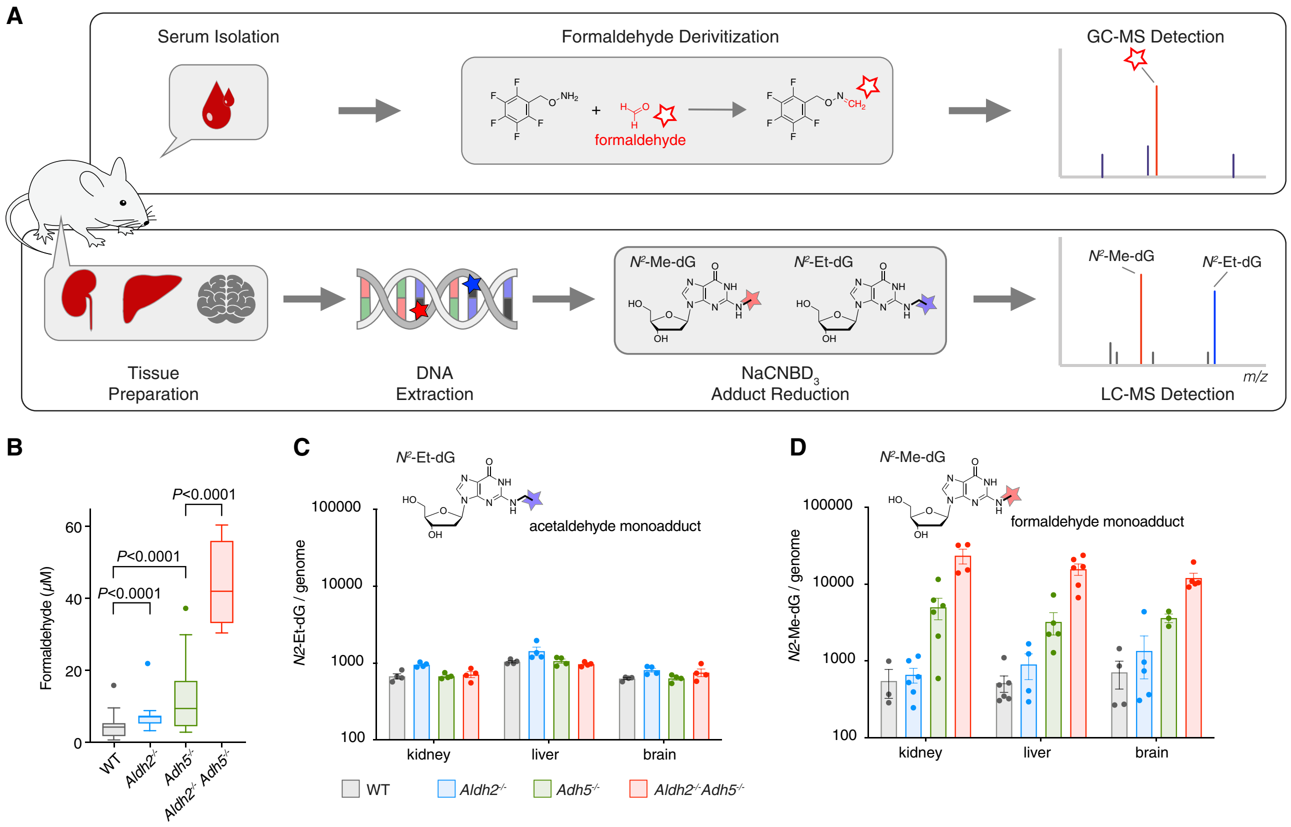

Figure 5. *Aldh2* and *Adh5* Act to Suppress Blood Formaldehyde Levels and Its DNA Adduct in Tissues

(A) Scheme of formaldehyde quantification in serum and as DNA adduct in tissues.

(B) Serum levels of formaldehyde (n = 43, 20, 51, 4, left to right). Boxes with lines indicate quartiles and median, and Tukey whiskers extend to 1.5 interquartile ranges. Two-tailed Mann-Whitney *U* test.

(C) Determination of the reduced genomic AA-deoxyguanine adduct N^2-ethyl-deoxyguanosine from kidneys, liver, and brain (mean ± SEM; n = 4 per group).

(D) Determination of the reduced genomic formaldehyde-deoxyguanine adduct N^2-methyl-deoxyguanosine from mouse kidneys, liver, and brain (mean ± SEM; n = 3–6 per group).

See also Figure S5.

WT, with no obvious skew in size distribution (Figures 6B–6D, S6A, and S6B). We then analyzed the single-nucleotide substitutions in more detail. First, the mutation profile of the WT HSPCs is very similar to that extracted from human HSPCs from a 50-year-old man (Lee-Six et al., 2018), with C-to-T being the predominant change (about 40% of which are in a CpG context; Figure S6C). However, there is a notable and consistent increase in T-to-A transversions and, to a lesser extent, T-to-C transitions in *Aldh2*$^{-/-}$*Adh5*$^{-/-}$ progenitors, which stand out (Figure 6E). By cosine similarity, the formaldehyde-induced mutational spectrum was most similar to the cancer-derived single-base substitution signatures SBS25, SBS40, SBS5, and SBS3 (Figure S6D; Alexandrov et al., 2020). Furthermore, T-to-A and T-to-G transversions showed a strong bias for an adenine base on the transcribed strand (Figure S6E), a feature shared with SBS40 that contributes to multiple cancers but is so far of unknown etiology. In summary, *Aldh2*$^{-/-}$*Adh5*$^{-/-}$ mice show formaldehyde accumulation in their serum; this correlates with an increase in formaldehyde-modified DNA in tissues and mutational signatures with similarity to patterns observed in human cancers.

Inherited ALDH2 and ADH5 Deficiency in Humans Causes Bone Marrow Failure and Myelodysplasia

The devastating phenotype in mice resulting from failure of formaldehyde clearance prompted us to ask whether there are human diseases that could arise from loss of ALDH2 and ADH5. Based on our previous work suggesting that endogenous formaldehyde might be the genotoxin that causes FA, we predicted that a human disease analogous to *Aldh2*$^{-/-}$*Adh5*$^{-/-}$ mice could present as early-onset bone marrow failure in children. We focused our search on the East Asian population, where a high proportion of people (40%–50%) already carry the *ALDH2*2* allele (Eng et al., 2007). This allele is defined by a functional SNP: rs671, the c.1510G > A mutation encoding a E504K amino acid substitution that reduces the enzymatic activity by ~90% in a dominant-negative manner (Crabb et al., 1989). Individuals carrying the *ALDH2*2* allele develop facial flushing after consumption of alcohol and have an increased risk of esophageal cancer (Brooks et al., 2009).

Through a combination of whole-exome sequencing (WES) and targeted exome sequencing of the *ADH5* gene, we

CellPress
OPEN ACCESS

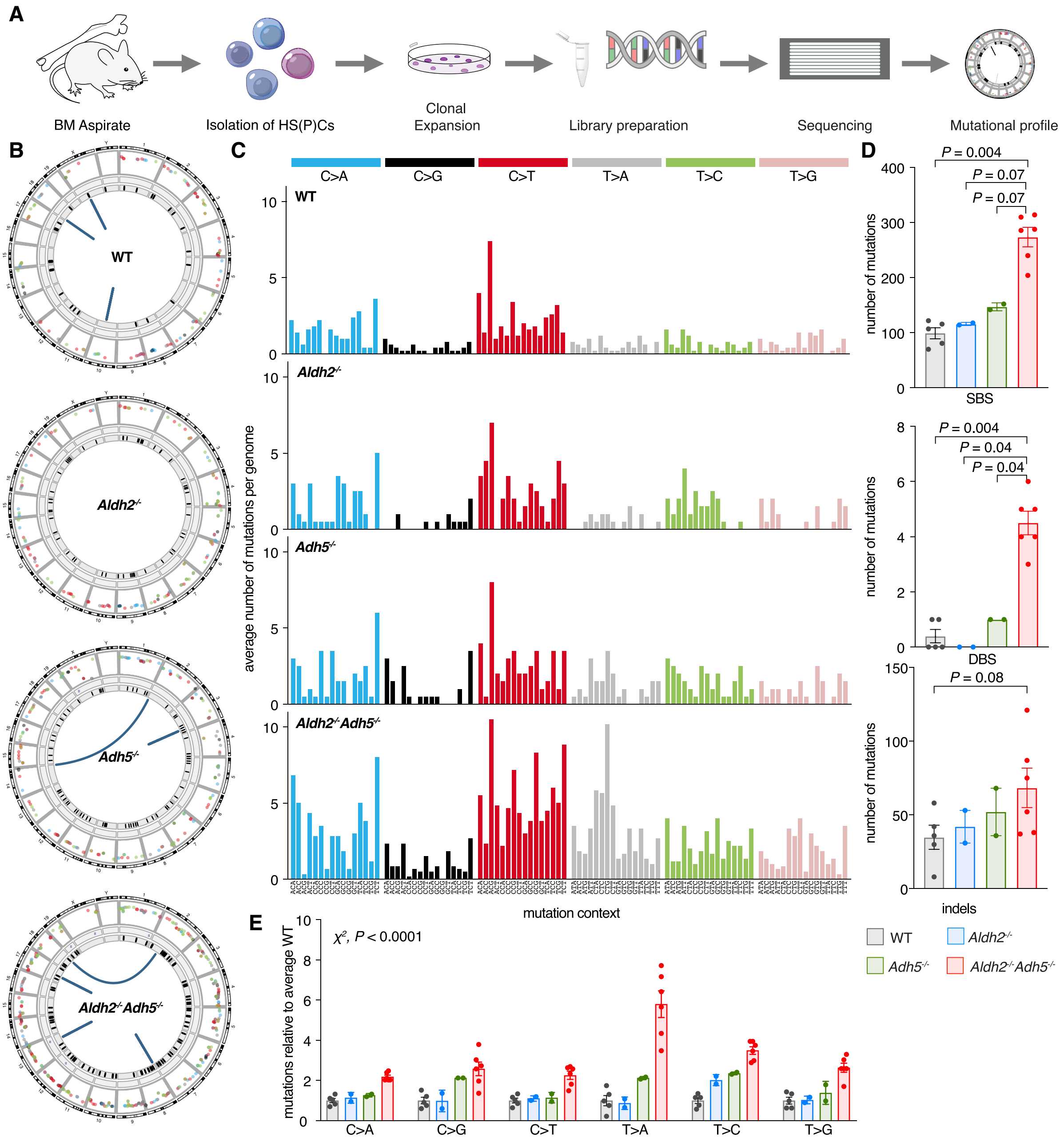

Figure 6. Formaldehyde-Accumulating *Aldh2*$^{-/-}$*Adh5*$^{-/-}$ Mice Reveal a Mutation Signature

(A) Whole-genome sequencing of HSPCs.

(B) Circos plots highlighting the different types and levels of mutations from a representative *Aldh2*$^{-/-}$*Adh5*$^{-/-}$ mouse and controls. The outermost ring represents each chromosome, followed by sequential rings highlighting single-base substitutions (SBSs) as a rainfall plot (color-coding of substitution types as in C), tandem base substitutions (DBSs), and insertions or deletions (indels). Chromosomal rearrangements are represented by lines linking the translocated chromosomes at the center.

(C) Aggregated mutational profile of SBSs in HSPC genomes. Each mutation is assigned to the pyrimidine base of the originating base pair; within each of the 6 main mutation types, the sequence context of 5′ and 3′ flanking bases is shown in alphabetical order.

(D) Frequency of SBSs, DBSs, and indels (mean ± SEM; number of HSPC genomes analyzed = 5, 2, 2, 6 from left to right; two-tailed Mann-Whitney *U* test).

(E) Relative mutation number at each base, normalized to the average HSPC clone from WT litter-matched 40-week-old animals (mean ± SEM; n = 5, 2, 2, 6 from left to right; χ^2 test comparing the aggregate number of mutations of each type between the WT and *Aldh2*$^{-/-}$*Adh5*$^{-/-}$).

See also Figure S6.

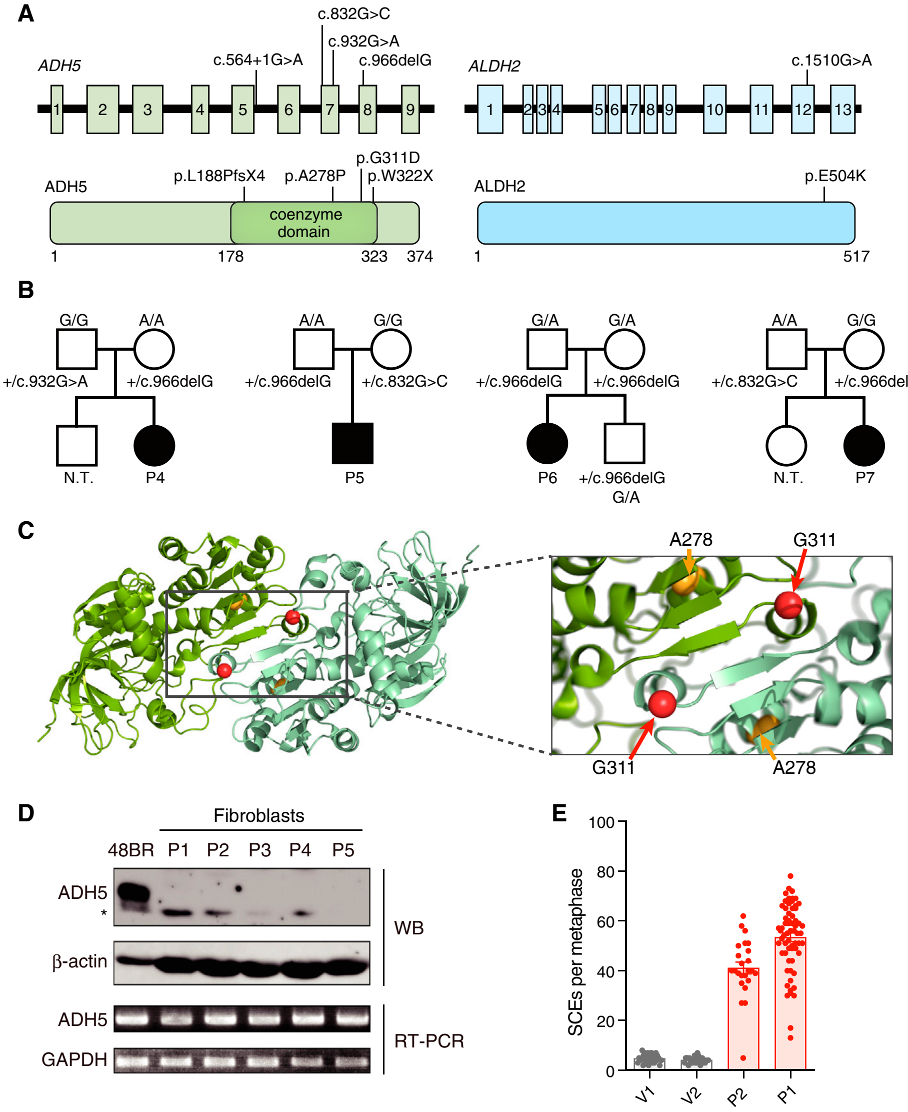

Figure 7. Human Patients with Bone Marrow Failure Syndrome Caused by Inactivating Mutations in *ALDH2* and *ADH5*

(A) Location of mutations in the *ADH5* and *ALDH2* genes (top) and proteins (bottom).

(B) Family pedigree of patients P4–P7. All parents were heterozygous for *ADH5* mutations and reported to be healthy regardless of *ALDH2* genotype. N.T., not tested.

(C) Localization of missense mutations near the ADH5 dimer interface.

(D) *ADH5* gene expression in fibroblasts from patients P1–P5 by protein and RNA. An asterisk denotes a non-specific band recognized by the antibody.

(E) SCEs per metaphase (mean ± SEM) in patient-derived, PHA-stimulated lymphoblasts (P1 and P2) and two unrelated *ALDH2*1/*2* heterozygous volunteers (V1 and V2).

See also Figure S7.

sequenced children and young adults with bone marrow failure of unknown etiology from the Japanese Cancer Research Resources Bank (JCRB; Osaka, Japan) and our local centers. Of the 14 patients analyzed, seven harbored bi-allelic *ADH5* mutations. Pathogenic alterations in genes known to be associated with other inherited bone marrow failure syndromes (IBMFS) (Bluteau et al., 2018) were not detected (data not shown). Interestingly, all seven of these IBMFS cases were also heterozygous for the *ALDH2*2* allele (the normal 1510G allele is called the *ALDH2*1* or G allele) (Figure 7A; Gross et al., 2015). Available clinical and laboratory data for these cases and the family pedigrees are summarized in Table 1 and Figure 7B. Detailed clinical information for the original three individuals was not available. However, all of them were adolescent patients with aplastic anemia (AA); moreover, the latter four developed myelodysplastic syndrome (MDS) that required HSC transplantation (HSCT), and in one patient, this progressed to acute myeloid leukemia (AML). Overall, their hematological phenotype (i.e., AA and MDS/AML), short stature, and skin pigmentation resembled FA. However, importantly, cells obtained from these patients

Table 1. Summary of Japanese Patients Carrying Mutations in *ADH5* and *ALDH2* Genes

Case No.: ID	Age (Years)/ Gender	*ADH5* Mutations	*ALDH2* Genotype	Chromosome Breakage Test	Hematological Pathology	Bone Marrow Cytogenetics	Treatment and Outcome	Birth Weight and Stature	Other Clinical Features
P1: AP39P	10/F	c.564+1G > A: p.L188PfsX4 c.832G > C: p.A278P (het)	G/A	0.21 per cell (MMC)	–	–	–		
P2: AP57P	13/M	c.966delG: p.W322X c.832G > C: p.A278P (het)	G/A	0.05 per cell (MMC)	–	–	–		
P3: FA50P	19/F	c.966delG: p.W322X (hom)	G/A	0.11 per cell (MMC)	–	–	–		
P4: TKFA-18	1/F	c.966delG: p.W322X c.932G > A: p.G311D (het)	G/A	0.00 per cell (DEB)	AA progressing to MDS (RCMD) at age 10 years	46,XX,der(22)t(1;22)(q12;q13),der(22)t(1;22)(q12;q13)[20]	HSCT at age 11 years, ongoing remission at 59 months post-HSCT	birthweight, 2,616 g; −1.48 SD; short stature, −4.9 SD at 138 months	skin pigmentation, café au lait spots, mild mental retardation
P5: TKFB-09	15/M	c.966delG: p.W322X c.832G > C: p.A278P (het)	G/A	0.01 per cell (DEB)	AA progressing to AML	46,XY,+1,der(1;15)(q10:q10),del(7)(q?),add(11)(q23)[19]/46,XY[1]	HSCT at age 16 years, died 60 months post-HSCT	birthweight, 2,784 g; −1.31 SD; short stature, −3.4 SD at 182 months	skin pigmentation, vitiligo, mild mental retardation
P6: KDFA-08	16/F	c.966delG: p.W322X (hom)	G/A	N.T.	AA with MDS (RCMD)	46,XX,der(14)t(1;14)(q12;p11.2),der(21)t(1;21)(q12;p11.2)[19/20]	first and second HSCT at age 18 and 19 years, ongoing remission at 6 months after second HSCT	birthweight, 2,730 g; −0.37 SD; short stature, −2.4 SD at 194 months	short left fourth toe, microcephaly, mild mental retardation
P7: KDFA-13	4/F	c.966delG: p.W322X c.832G > C: p.A278P (Het)	G/A	0.07 per cell (MMC)	AA with MDS (RAEB2)	46,XX,+1,der(1;7)(q10;p10)[20/20]	HSCT at age 4 years, ongoing remission	birthweight, 2,935 g; −0.34 SD; short stature, −2.0 SD at 49 months	skin pigmentation, café au lait spots, microcephaly, mild mental retardation

F, female; M, male; N.T., not tested; MMC, mitomycin C; DEB, diepoxybutane; AA, aplastic anemia; MDS, myelodysplastic syndrome; RCMD, refractory cytopenia with multi-lineage dysplasia; RAEB2, refractory anemia with excess blast 2; AML, acute myeloid leukemia; HSCT, hematopoietic stem cell transplant; SD, standard deviation from local population median. See also Table S7.

did not show elevated chromosome breakage following exposure to the DNA crosslinking agents mitomycin C (MMC) or diepoxybutane (DEB) (Table 1). This indicates that DNA crosslink repair is intact in these individuals and that the cause of their bone marrow failure could not be a mutation in a new FA gene.

Among the four identified *ADH5* variations (Figures 7A and 7B; Table S6), two were very rare and found only in a small number of East Asians in the genome aggregation database gnomAD (Karczewski et al., 2020), whereas the other two have not been described previously. No individuals homozygous or compound heterozygous for these variants were found in the databases or in our previous WES analysis in Japanese IBMFS patients (Muramatsu et al., 2017). Interestingly, the two missense variants were located close to the interface of the ADH5 dimer (Figure 7C). All variants abolish ADH5 protein expression; the corresponding fibroblast cultures had undetectable ADH5 protein by western blotting, but transcript levels were not affected severely (Figure 7D). The c.564+1G > A mutation in patient 1 (AP39P) affected splicing (Figure S7A). Exogenously expressed FLAG-tagged A278P ADH5 was barely detected in HEK293T cells, and neither the FLAG-tagged A278P ADH5 nor a missense G311D-FLAG ADH5 co-immunoprecipitated with co-expressed WT GFP-tagged ADH5 (Figure S7B). We wanted to assess whether DNA damage and repair was also elevated in these patients as in *Aldh2*$^{-/-}$*Adh5*$^{-/-}$ mice by measuring the number of SCEs in phytohemagglutinin (PHA)-stimulated, patient-derived T lymphoblasts. Strikingly, the number of SCEs in patient cells was induced vigorously by about 10-fold (Figure 7E), which is similar to the levels seen in patients with Bloom syndrome (Chaganti et al., 1974), a genome instability syndrome where HR repair is induced. We found patients harboring mutations in *ALDH2* and *ADH5*, resulting in a previously uncharacterized IBMFS, which highlights the essential requirement for metabolic clearance of formaldehyde in human hematopoiesis.

DISCUSSION

This study establishes the scale of endogenous formaldehyde production and the routes through which it is removed. We identify two detoxifying enzymes, mitochondrial ALDH2 and cytosolic ADH5, that are jointly essential for removing formaldehyde. Loss of these detoxification mechanisms leads to hematopoietic failure and leukemia in mice and humans by overwhelming DNA repair, leading to genome instability and somatic mutations.

Formaldehyde-processing enzymes are widely expressed across tissues, including high expression in HSPCs (Figure 1A), but differ in their subcellular localization, which may point toward distinct sites of production of cellular formaldehyde. Given the level of formaldehyde that we detected in blood (4–44 μM), this is clearly an abundantly produced molecule. Decomposition of the vitamin folic acid (Burgos-Barragan et al., 2017), a cofactor of 1C metabolism, could be a mitochondrial source. Other likely sources could be oxidative demethylation reactions of DNA, RNA, and histones. Such epigenetic modification may be very active in developing hematopoietic cells, leading to spikes of endogenous formaldehyde being produced in the vicinity of DNA. For many years, formaldehyde has been considered an environmental carcinogen as a by-product of industrial processes, and exposure to such potential sources has been found to be associated with leukemia (Beane Freeman et al., 2009). However, environmental sources are very unlikely in our murine studies, indicating that 4 μM must be generated from within the animal. An important future area of research is to better define where within us all of this formaldehyde comes from.

The grave and diverse phenotype of *Aldh2*$^{-/-}$*Adh5*$^{-/-}$ mice is very likely driven by accumulation of toxic formaldehyde, but it is not clear whether these features can be explained by genotoxicity alone. Perinatal lethality and growth retardation as observed in the *Aldh2*$^{-/-}$*Adh5*$^{-/-}$ mice are common features of DNA repair-deficient mice and humans (Weeda et al., 1997), and it is tempting to speculate that endogenous formaldehyde may be responsible for at least some of the lesions these repair pathways deal with. Our analysis shows that formaldehyde accumulation has widespread consequences on blood production; there is significant perturbation of the transcriptomes of HSCs and early progenitors and a profound effect on development of the acquired immune system. Although these hematopoietic features could be due to DNA damage in this compartment, in good agreement with previous work showing specific depletion of lymphoid cells in response to exogenous DNA damage (Wang et al., 2016), it is also known that formaldehyde can modify RNA and proteins as well, which might add to the broad consequences of hematopoietic instruction.

An important aspect of our study is the emergence of a mutation signature associated with formaldehyde accumulation. Two particular aspects stand out. First, there is a marked increase across all classes of single-nucleotide substitutions in a profile that is similar to the cancer mutation signatures SBS5 and SBS40. This is a ubiquitous signature without known cause that is present in virtually all cancer genomes and certain normal somatic tissues, correlates with age, and has been speculated to reflect damage to DNA caused by ubiquitous metabolic driver(s) (Alexandrov et al., 2015, 2020; Blokzijl et al., 2016; Kim et al., 2016; Lee-Six et al., 2019; Moore et al., 2020). Formaldehyde thus seems to be a likely contributor shaping this common signature; this could be directly by causing DNA damage or indirectly by driving stem cell attrition and premature aging. However, previous work using an FA-deficient mouse model with a more severe stem cell defect and anemia showed that a more modest induction of point mutations and rescue of the stem cell defect via deletion of p53 did not reduce the mutation burden, arguing against stem cell attrition as the cause of mutation (Garaycoechea et al., 2018). Second, the number of T-to-A transversions and, to a lesser extent, T-to-C transitions stand out in *Aldh2*$^{-/-}$*Adh5*$^{-/-}$ genomes. This suggests that formaldehyde may preferentially adduct adenine, possibly through attack to its exocyclic amine. A recent study implicated a novel mechanism by which formaldehyde exerts genotoxic activity. Cells were exposed to exogenous formaldehyde in excess of 100 μM, causing instability and inactivation of the key recombination and tumor suppressor protein BRCA2 (Tan et al., 2017). However, it is very unlikely that this is what occurs in *Aldh2*$^{-/-}$*Adh5*$^{-/-}$ mice or in the deficient humans we describe here. First, we detected a striking induction of BRCA2-mediated DNA repair by rise of spontaneous SCEs in mice and humans. Second, the formaldehyde mutation signature we uncover here does not

resemble that observed in BRCA2-deficient tumors. Third, our measurements of blood formaldehyde levels show that the physiological range is 4–10 μM when detoxification is intact, considerably lower than the dose range used in the published exposure studies.

Our previous work has shown that endogenous formaldehyde might be a driver for the phenotype of FA (Pontel et al., 2015; Rosado et al., 2011). We discovered seven human families carrying genetic defects in *ALDH2* and *ADH5*, presenting as a new bone marrow failure syndrome that is solely driven by formaldehyde accumulation. Although the causes of IBMFS are diverse and include FA (Kottemann and Smogorzewska, 2013; Duxin and Walter, 2015; Ceccaldi et al., 2016), telomere biology disorders (Savage and Alter, 2009), and ribosome assembly defects (Kampen et al., 2020; Kennedy and Shimamura, 2019; Narla and Ebert, 2010), we believe that this is the first example of an IBMFS arising through a purely metabolic route. Given that formaldehyde is likely the common driver in FA and the IBMFS described in our work, it is not surprising that both diseases have common clinical features. As more cases of this new IBMFS are described, it will be of interest to assess any clinical features distinct from FA, indicating whether formaldehyde-driven pathologies can arise independent of failure of DNA crosslink repair. In our analysis, an unexpected finding was the high frequency (50%; 7 of 14 patients analyzed) of children and young adults with bone marrow failure of unknown etiology who carried mutations in *ALDH2* and *ADH5*. We therefore recommend genotyping for these mutations as part of diagnostic investigations in future management of IBMFS patients of East Asian ethnicity. In addition, therapy aiming to lower endogenous formaldehyde could be a promising treatment strategy for this disease as well as for FA. Notably, ALDH2 deficiency has been associated with increased risk of esophageal, head and neck, and liver cancer in alcohol-consuming individuals (Brooks et al., 2009; Matsuo et al., 2001; Seo et al., 2019; Yokoyama et al., 2001). However, although FA patients with ALDH2 deficiency show more rapid disease progression (Hira et al., 2013), this effect is unlikely to be attributable to alcohol consumption because the patients are children. The present work raises the possibility that formaldehyde, rather than alcohol-derived acetaldehyde, might be responsible for this effect.

Finally, we establish reliable methods to track endogenous formaldehyde in blood and its adducts on DNA. These methods can now be used to probe in more detail how endogenous formaldehyde varies in humans and other mammals as well in other stressed physiological states. It is notable in this context that approximately 500 million humans are deficient in ALDH2 activity and may therefore accumulate endogenous formaldehyde (Oota et al., 2004), potentially in a manner that may interact with heritable polymorphisms in other loci or with specific environmental exposure. It is possible that this may have consequences for the long-term well-being of these individuals.

Limitations of Study

We find that strict adherence to the present protocol and rapid processing is essential for obtaining reliable formaldehyde quantification in serum. Although sequencing of HSPC-derived clones shows that DNA damage as mutations is increased in these cells, the assay does not discriminate between stem and progenitor cells, and our data suggest that both compartments are affected by formaldehyde accumulation. Furthermore, because the double-mutant mice show a defect in stem and progenitor populations, we cannot at present disentangle the direct effect of formaldehyde accumulation on the genome from its indirect effects mediated by the pathophysiological changes in $Aldh2^{-/-}Adh5^{-/-}$ animals. Although we see induction of the DNA damage response and DNA repair gene expression in $Aldh2^{-/-}Adh5^{-/-}$ stem and progenitor cells, the precise nature of formaldehyde-derived DNA damage and its sensing mechanism as well as the origin of endogenous formaldehyde remain areas of future study.

STAR★METHODS

Detailed methods are provided in the online version of this paper and include the following:

- KEY RESOURCES TABLE
- RESOURCE AVAILABILITY
  - Lead Contact
  - Materials Availability
  - Data and Code Availability
- EXPERIMENTAL MODEL AND SUBJECT DETAILS
  - Mice
  - Research subjects
- METHOD DETAILS
  - Blood counts
  - Histology and Immunohistochemistry
  - Single cell RNA-seq
  - Single cell expression analysis
  - Heatmap Visualization of Adh and Aldh Family Genes
  - Quantification of HSC numbers across Genotypes
  - Quantification of HSC state using hscScore
  - Subclustering of HSPC cluster
  - Cell cycle profiling
  - Apoptosis module score
  - DNA repair gene expression analysis
  - Flow cytometry
  - Competitive repopulation assay
  - Micronucleus assay
  - Sister chromatid exchange assay
  - Methanol treatment for hematopoietic development
  - ALDH2 biochemistry
  - Mouse serum formaldehyde quantification by GC–MS
  - Synthesis of nucleoside standards
  - Sample preparation for determination of N^2-MeG in DNA
  - Online LC-MS^2 determination of N^2-MeG in DNA digests
  - Detection of acetaldehyde mono-adduct N^2-EtdG by MS
  - Genome sequencing of HSPC colonies
  - Patient-derived cell culture and transfection

- ○ SCE assay on patient-derived cells
- ○ Immunoprecipitation and western blotting of patient-derived cells
- QUANTIFICATION AND STATISTICAL ANALYSIS

SUPPLEMENTAL INFORMATION

Supplemental Information can be found online at https://doi.org/10.1016/j.molcel.2020.10.012.

ACKNOWLEDGMENTS

We thank the LMB ARES animal research staff and genotyping service, Emeritus Prof. M.S. Sasaki for deposition of the samples and data to the JCRB, T. Hirayama and Dr. A. Kohara at the JCRB Cell Bank for help, and Prof. G.S. Vassiliou and Prof. A.J. Warren for critical reading of the manuscript. This work was supported by funding to F.A.D. and M.W. (C60150/A23919) by CRUK, to L.P. and R.Y. by the FA Research Fund (5102402), to R.Y. in part by NIEHS grants P42 ES005948 and P30 ES010126 and TCEQ (582-12-21861), and to K.J.P. and group by the MRC, the Wellcome Trust, and the Jeffrey Cheah Foundation. This work was also supported by JSPS KAKENHI grants (JP23114010, JP26550026, and JP15H01738 to M.T. and 16K15243 to A.H.) and grants from The Ministry of Health, Labour and Welfare (to M.T., M.Y., H.Y., E.I., and S.K.), the Japan Leukemia Research Fund (to A.H.), the Takeda Science Foundation (to A.H.), the Uehara Memorial Foundation (to M.T.), the Astellas Foundation for Research on Metabolic Disorders (to M.T.), and The Kyoto University Research Fund (Core Stage Back-Up) (to M.T.). The Radiation Biology Center, Graduate School of Biostudies, Kyoto University, is a joint-usage research center certified by the MEXT and is also supported by the JSPS Core-to-Core Program (JPJSCCA20200009).

AUTHOR CONTRIBUTIONS

K.J.P. conceived the project. F.A.D. and M.W. led the project with assistance from N.O., L.B.P., A.N.K.-L., F.L., C.N., and M.R.G.H. C.L.M., M.R.H.H., and R.Y. completed the adduct analysis. R.L.C. and P.S.M. measured serum formaldehyde. S.W., N.K.W., and B.G. performed scRNA-seq. The human study was planned by M.T., K.M., M.Y., H.Y., S.O., S.M., S.K., and E.I. K.Y., Y. Okuno, H.M., and Y.S. performed WES. M.Y., H.Y., M. Kobayashi, T.M., T.O., and M. Kato provided clinical samples and data. A.M. and A.H. completed the majority of the experiments on human samples with help from Y. Okamoto and M.M. K.J.P. wrote the manuscript with input from M.W., F.A.D., A.M., and M.T. All authors provided comments on the manuscript.

DECLARATION OF INTERESTS

The authors declare no competing interests.

Received: June 15, 2020
Revised: August 20, 2020
Accepted: October 8, 2020
Published: November 4, 2020

REFERENCES

Alexandrov, L.B., Jones, P.H., Wedge, D.C., Sale, J.E., Campbell, P.J., Nik-Zainal, S., and Stratton, M.R. (2015). Clock-like mutational processes in human somatic cells. Nat. Genet. *47*, 1402–1407.

Alexandrov, L.B., Kim, J., Haradhvala, N.J., Huang, M.N., Tian Ng, A.W., Wu, Y., Boot, A., Covington, K.R., Gordenin, D.A., Bergstrom, E.N., et al.; PCAWG Mutational Signatures Working Group; PCAWG Consortium (2020). The repertoire of mutational signatures in human cancer. Nature *578*, 94–101.

Beane Freeman, L.E., Blair, A., Lubin, J.H., Stewart, P.A., Hayes, R.B., Hoover, R.N., and Hauptmann, M. (2009). Mortality from lymphohematopoietic malignancies among workers in formaldehyde industries: the National Cancer Institute Cohort. J. Natl. Cancer Inst. *101*, 751–761.

Blokzijl, F., de Ligt, J., Jager, M., Sasselli, V., Roerink, S., Sasaki, N., Huch, M., Boymans, S., Kuijk, E., Prins, P., et al. (2016). Tissue-specific mutation accumulation in human adult stem cells during life. Nature *538*, 260–264.

Bluteau, O., Sebert, M., Leblanc, T., Peffault de Latour, R., Quentin, S., Lainey, E., Hernandez, L., Dalle, J.-H., Sicre de Fontbrune, F., Lengline, E., et al. (2018). A landscape of germ line mutations in a cohort of inherited bone marrow failure patients. Blood *131*, 717–732.

Brooks, P.J., Enoch, M.-A., Goldman, D., Li, T.-K., and Yokoyama, A. (2009). The alcohol flushing response: an unrecognized risk factor for esophageal cancer from alcohol consumption. PLoS Med. *6*, e50.

Bryce, S.M., Bemis, J.C., Avlasevich, S.L., and Dertinger, S.D. (2007). In vitro micronucleus assay scored by flow cytometry provides a comprehensive evaluation of cytogenetic damage and cytotoxicity. Mutat. Res. *630*, 78–91.

Burgos-Barragan, G., Wit, N., Meiser, J., Dingler, F.A., Pietzke, M., Mulderrig, L., Pontel, L.B., Rosado, I.V., Brewer, T.F., Cordell, R.L., et al. (2017). Mammals divert endogenous genotoxic formaldehyde into one-carbon metabolism. Nature *548*, 549–554.

Cancho, B., Ventura, F., and Galceran, M.T. (2002). Determination of aldehydes in drinking water using pentafluorobenzylhydroxylamine derivatization and solid-phase microextraction. J. Chromatogr. A *943*, 1–13.

Ceccaldi, R., Sarangi, P., and D'Andrea, A.D. (2016). The Fanconi anaemia pathway: new players and new functions. Nat. Rev. Mol. Cell Biol. *17*, 337–349.

Chaganti, R.S., Schonberg, S., and German, J. (1974). A manyfold increase in sister chromatid exchanges in Bloom's syndrome lymphocytes. Proc. Natl. Acad. Sci. USA *71*, 4508–4512.

Crabb, D.W., Edenberg, H.J., Bosron, W.F., and Li, T.K. (1989). Genotypes for aldehyde dehydrogenase deficiency and alcohol sensitivity. The inactive ALDH2(2) allele is dominant. J. Clin. Invest. *83*, 314–316.

Dahlin, J.S., Hamey, F.K., Pijuan-Sala, B., Shepherd, M., Lau, W.W.Y., Nestorowa, S., Weinreb, C., Wolock, S., Hannah, R., Diamanti, E., et al. (2018). A single-cell hematopoietic landscape resolves 8 lineage trajectories and defects in Kit mutant mice. Blood *131*, e1–e11.

Duxin, J.P., and Walter, J.C. (2015). What is the DNA repair defect underlying Fanconi anemia? Curr. Opin. Cell Biol. *37*, 49–60.

Eng, M.Y., Luczak, S.E., and Wall, T.L. (2007). ALDH2, ADH1B, and ADH1C genotypes in Asians: a literature review. Alcohol Res. Health *30*, 22–27.

Garaycoechea, J.I., Crossan, G.P., Langevin, F., Daly, M., Arends, M.J., and Patel, K.J. (2012). Genotoxic consequences of endogenous aldehydes on mouse haematopoietic stem cell function. Nature *489*, 571–575.

Garaycoechea, J.I., Crossan, G.P., Langevin, F., Mulderrig, L., Louzada, S., Yang, F., Guilbaud, G., Park, N., Roerink, S., Nik-Zainal, S., et al. (2018). Alcohol and endogenous aldehydes damage chromosomes and mutate stem cells. Nature *553*, 171–177.

Garcia, C.C.M., Angeli, J.P.F., Freitas, F.P., Gomes, O.F., de Oliveira, T.F., Loureiro, A.P.M., Di Mascio, P., and Medeiros, M.H.G. (2011). [13C2]-Acetaldehyde promotes unequivocal formation of 1,N2-propano-2′-deoxyguanosine in human cells. J. Am. Chem. Soc. *133*, 9140–9143.

Giri, S.D., and Chatterjee, A. (1998). Modulation of mitomycin C-induced sister chromatid exchanges and cell cycle delay by buthionine sulfoximine and reduced glutathione in mouse bone marrow cells in vivo. Mutat. Res. *413*, 227–234.

Gross, E.R., Zambelli, V.O., Small, B.A., Ferreira, J.C.B., Chen, C.-H., and Mochly-Rosen, D. (2015). A personalized medicine approach for Asian Americans with the aldehyde dehydrogenase 2*2 variant. Annu. Rev. Pharmacol. Toxicol. *55*, 107–127.

Hamajima, N., Matsuo, K., Saito, T., Hirose, K., Inoue, M., Takezaki, T., Kuroishi, T., and Tajima, K. (2001). Gene-environment Interactions and Polymorphism Studies of Cancer Risk in the Hospital-based Epidemiologic Research Program at Aichi Cancer Center II (HERPACC-II). Asian Pac. J. Cancer Prev. *2*, 99–107.

Hamey, F.K., and Göttgens, B. (2019). Machine learning predicts putative hematopoietic stem cells within large single-cell transcriptomics data sets. Exp. Hematol. *78*, 11–20.

Harada, S., Agarwal, D.P., and Goedde, H.W. (1981). Aldehyde dehydrogenase deficiency as cause of facial flushing reaction to alcohol in Japanese. Lancet *2*, 982.

Heck, H.D., Casanova-Schmitz, M., Dodd, P.B., Schachter, E.N., Witek, T.J., and Tosun, T. (1985). Formaldehyde (CH2O) concentrations in the blood of humans and Fischer-344 rats exposed to CH2O under controlled conditions. Am. Ind. Hyg. Assoc. J. *46*, 1–3.

Hira, A., Yabe, H., Yoshida, K., Okuno, Y., Shiraishi, Y., Chiba, K., Tanaka, H., Miyano, S., Nakamura, J., Kojima, S., et al. (2013). Variant ALDH2 is associated with accelerated progression of bone marrow failure in Japanese Fanconi anemia patients. Blood *122*, 3206–3209.

Hodskinson, M.R., Bolner, A., Sato, K., Kamimae-Lanning, A.N., Rooijers, K., Witte, M., Mahesh, M., Silhan, J., Petek, M., Williams, D.M., et al. (2020). Alcohol-derived DNA crosslinks are repaired by two distinct mechanisms. Nature *579*, 603–608.

Jackson, B., Brocker, C., Thompson, D.C., Black, W., Vasiliou, K., Nebert, D.W., and Vasiliou, V. (2011). Update on the aldehyde dehydrogenase gene (ALDH) superfamily. Hum. Genomics *5*, 283–303.

Jacobson, M.K., and Bernofsky, C. (1974). Mitochondrial acetaldehyde dehydrogenase from Saccharomyces cerevisiae. Biochim. Biophys. Acta *350*, 277–291.

Kampen, K.R., Sulima, S.O., Vereecke, S., and De Keersmaecker, K. (2020). Hallmarks of ribosomopathies. Nucleic Acids Res. *48*, 1013–1028.

Karczewski, K.J., Francioli, L.C., Tiao, G., Cummings, B.B., Alföldi, J., Wang, Q., Collins, R.L., Laricchia, K.M., Ganna, A., Birnbaum, D.P., et al. (2020). The mutational constraint spectrum quantified from variation in 141,456 humans. Nature *581*, 434–443.

Kennedy, A.L., and Shimamura, A. (2019). Genetic predisposition to MDS: clinical features and clonal evolution. Blood *133*, 1071–1085.

Kim, J., Mouw, K.W., Polak, P., Braunstein, L.Z., Kamburov, A., Kwiatkowski, D.J., Rosenberg, J.E., Van Allen, E.M., D'Andrea, A., and Getz, G. (2016). Somatic ERCC2 mutations are associated with a distinct genomic signature in urothelial tumors. Nat. Genet. *48*, 600–606.

Kottemann, M.C., and Smogorzewska, A. (2013). Fanconi anaemia and the repair of Watson and Crick DNA crosslinks. Nature *493*, 356–363.

Langevin, F., Crossan, G.P., Rosado, I.V., Arends, M.J., and Patel, K.J. (2011). Fancd2 counteracts the toxic effects of naturally produced aldehydes in mice. Nature *475*, 53–58.

Lee-Six, H., Øbro, N.F., Shepherd, M.S., Grossmann, S., Dawson, K., Belmonte, M., Osborne, R.J., Huntly, B.J.P., Martincorena, I., Anderson, E., et al. (2018). Population dynamics of normal human blood inferred from somatic mutations. Nature *561*, 473–478.

Lee-Six, H., Olafsson, S., Ellis, P., Osborne, R.J., Sanders, M.A., Moore, L., Georgakopoulos, N., Torrente, F., Noorani, A., Goddard, M., et al. (2019). The landscape of somatic mutation in normal colorectal epithelial cells. Nature *574*, 532–537.

Liu, L., Yan, Y., Zeng, M., Zhang, J., Hanes, M.A., Ahearn, G., McMahon, T.J., Dickfeld, T., Marshall, H.E., Que, L.G., and Stamler, J.S. (2004). Essential roles of S-nitrosothiols in vascular homeostasis and endotoxic shock. Cell *116*, 617–628.

Luo, W., Li, H., Zhang, Y., and Ang, C.Y. (2001). Determination of formaldehyde in blood plasma by high-performance liquid chromatography with fluorescence detection. J. Chromatogr. B Biomed. Sci. Appl. *753*, 253–257.

Martos, P.A., and Pawliszyn, J. (1998). Sampling and determination of formaldehyde using solid-phase microextraction with on-fiber derivatization. Anal. Chem. *70*, 2311–2320.

Matsuo, K., Hamajima, N., Shinoda, M., Hatooka, S., Inoue, M., Takezaki, T., and Tajima, K. (2001). Gene-environment interaction between an aldehyde dehydrogenase-2 (ALDH2) polymorphism and alcohol consumption for the risk of esophageal cancer. Carcinogenesis *22*, 913–916.

Moeller, B.C., Lu, K., Doyle-Eisele, M., McDonald, J., Gigliotti, A., and Swenberg, J.A. (2011). Determination of N2-hydroxymethyl-dG adducts in the nasal epithelium and bone marrow of nonhuman primates following 13CD2-formaldehyde inhalation exposure. Chem. Res. Toxicol. *24*, 162–164.

Moeller, B.C., Recio, L., Green, A., Sun, W., Wright, F.A., Bodnar, W.M., and Swenberg, J.A. (2013). Biomarkers of exposure and effect in human lymphoblastoid TK6 cells following [13C2]-acetaldehyde exposure. Toxicol. Sci. *133*, 1–12.

Moore, L., Leongamornlert, D., Coorens, T.H.H., Sanders, M.A., Ellis, P., Dentro, S.C., Dawson, K.J., Butler, T., Rahbari, R., Mitchell, T.J., et al. (2020). The mutational landscape of normal human endometrial epithelium. Nature *580*, 640–646.

Muramatsu, H., Okuno, Y., Yoshida, K., Shiraishi, Y., Doisaki, S., Narita, A., Sakaguchi, H., Kawashima, N., Wang, X., Xu, Y., et al. (2017). Clinical utility of next-generation sequencing for inherited bone marrow failure syndromes. Genet. Med. *19*, 796–802.

Narla, A., and Ebert, B.L. (2010). Ribosomopathies: human disorders of ribosome dysfunction. Blood *115*, 3196–3205.

Oota, H., Pakstis, A.J., Bonne-Tamir, B., Goldman, D., Grigorenko, E., Kajuna, S.L.B., Karoma, N.J., Kungulilo, S., Lu, R.-B., Odunsi, K., et al. (2004). The evolution and population genetics of the ALDH2 locus: random genetic drift, selection, and low levels of recombination. Ann. Hum. Genet. *68*, 93–109.

Orsburn, B., Escudero, B., Prakash, M., Gesheva, S., Liu, G., Huso, D.L., and Franco, S. (2010). Differential requirement for H2AX and 53BP1 in organismal development and genome maintenance in the absence of poly(ADP)ribosyl polymerase 1. Mol. Cell. Biol. *30*, 2341–2352.

Pontel, L.B., Rosado, I.V., Burgos-Barragan, G., Garaycoechea, J.I., Yu, R., Arends, M.J., Chandrasekaran, G., Broecker, V., Wei, W., Liu, L., et al. (2015). Endogenous Formaldehyde Is a Hematopoietic Stem Cell Genotoxin and Metabolic Carcinogen. Mol. Cell *60*, 177–188.

Rosado, I.V., Langevin, F., Crossan, G.P., Takata, M., and Patel, K.J. (2011). Formaldehyde catabolism is essential in cells deficient for the Fanconi anemia DNA-repair pathway. Nat. Struct. Mol. Biol. *18*, 1432–1434.

Sasaki, M.S., and Tonomura, A. (1973). A high susceptibility of Fanconi's anemia to chromosome breakage by DNA cross-linking agents. Cancer Res. *33*, 1829–1836.

Savage, S.A., and Alter, B.P. (2009). Dyskeratosis congenita. Hematol. Oncol. Clin. North Am. *23*, 215–231.

Seo, W., Gao, Y., He, Y., Sun, J., Xu, H., Feng, D., Park, S.H., Cho, Y.-E., Guillot, A., Ren, T., et al. (2019). ALDH2 deficiency promotes alcohol-associated liver cancer by activating oncogenic pathways via oxidized DNA-enriched extracellular vesicles. J. Hepatol. *71*, 1000–1011.

Sonoda, E., Sasaki, M.S., Morrison, C., Yamaguchi-Iwai, Y., Takata, M., and Takeda, S. (1999). Sister chromatid exchanges are mediated by homologous recombination in vertebrate cells. Mol. Cell. Biol. *19*, 5166–5169.

Tan, S.L.W., Chadha, S., Liu, Y., Gabasova, E., Perera, D., Ahmed, K., Constantinou, S., Renaudin, X., Lee, M., Aebersold, R., and Venkitaraman, A.R. (2017). A Class of Environmental and Endogenous Toxins Induces BRCA2 Haploinsufficiency and Genome Instability. Cell *169*, 1105–1118.e15.

Tirosh, I., Izar, B., Prakadan, S.M., Wadsworth, M.H., 2nd, Treacy, D., Trombetta, J.J., Rotem, A., Rodman, C., Lian, C., Murphy, G., et al. (2016). Dissecting the multicellular ecosystem of metastatic melanoma by single-cell RNA-seq. Science *352*, 189–196.

Uotila, L., and Koivusalo, M. (1974). Formaldehyde dehydrogenase from human liver. Purification, properties, and evidence for the formation of glutathione thiol esters by the enzyme. J. Biol. Chem. *249*, 7653–7663.

Van der Auwera, G.A., Carneiro, M.O., Hartl, C., Poplin, R., Del Angel, G., Levy-Moonshine, A., Jordan, T., Shakir, K., Roazen, D., Thibault, J., et al. (2013). From FastQ data to high confidence variant calls: the Genome Analysis Toolkit best practices pipeline. Curr. Protoc. Bioinformatics *43*, 11.10.1–11.10.33.

Walter, D., Lier, A., Geiselhart, A., Thalheimer, F.B., Huntscha, S., Sobotta, M.C., Moehrle, B., Brocks, D., Bayindir, I., Kaschutnig, P., et al. (2015). Exit

from dormancy provokes DNA-damage-induced attrition in haematopoietic stem cells. Nature *520*, 549–552.

Wang, J., Morita, Y., Han, B., Niemann, S., Löffler, B., and Rudolph, K.L. (2016). Per2 induction limits lymphoid-biased haematopoietic stem cells and lymphopoiesis in the context of DNA damage and ageing. Nat. Cell Biol. *18*, 480–490.

Weeda, G., Donker, I., de Wit, J., Morreau, H., Janssens, R., Vissers, C.J., Nigg, A., van Steeg, H., Bootsma, D., and Hoeijmakers, J.H. (1997). Disruption of mouse ERCC1 results in a novel repair syndrome with growth failure, nuclear abnormalities and senescence. Curr. Biol. *7*, 427–439.

Wolf, F.A., Angerer, P., and Theis, F.J. (2018). SCANPY: large-scale single-cell gene expression data analysis. Genome Biol. *19*, 15.

Yabe, M., Yabe, H., Hamanoue, S., Inoue, H., Matsumoto, M., Koike, T., Ishiguro, H., Morimoto, T., Arakawa, S., Ohshima, T., et al. (2007). In vitro effect of fludarabine, cyclophosphamide, and cytosine arabinoside on chromosome breakage in Fanconi anemia patients: relevance to stem cell transplantation. Int. J. Hematol. *85*, 354–361.

Yokoyama, A., Muramatsu, T., Omori, T., Yokoyama, T., Matsushita, S., Higuchi, S., Maruyama, K., and Ishii, H. (2001). Alcohol and aldehyde dehydrogenase gene polymorphisms and oropharyngolaryngeal, esophageal and stomach cancers in Japanese alcoholics. Carcinogenesis *22*, 433–439.

Yu, R., Lai, Y., Hartwell, H.J., Moeller, B.C., Doyle-Eisele, M., Kracko, D., Bodnar, W.M., Starr, T.B., and Swenberg, J.A. (2015). Formation, Accumulation, and Hydrolysis of Endogenous and Exogenous Formaldehyde-Induced DNA Damage. Toxicol. Sci. *146*, 170–182.

CellPress
OPEN ACCESS

STAR★METHODS

KEY RESOURCES TABLE

REAGENT or RESOURCE	SOURCE	IDENTIFIER
Antibodies		
CD45R/B220 (clone RA3-6B2)	R&D Systems	RRID:AB_357537
c-Kit::APC-Cy7 (clone 2B8)	Biolegend	RRID:AB_1626278
Sca-1::BV421 (clone D7)	Biolegend	RRID:AB_2563064
CD45::FITC (clone 30-F11)	Biolegend	RRID:AB_312973
Flt3::PE (clone A2F10)	Biolegend	RRID:AB_1877217
Il-7Rα::BV605 (clone A7R34)	Biolegend	RRID:AB_2572047
Streptavidin::BV510	Biolegend	Cat#405234
CD4::FITC (clone H129.19)	BD Pharmingen	RRID:AB_394970
CD3e::FITC (clone 145-2C11)	eBioscience	RRID:AB_464882
Ly-6G/Gr-1::FITC (clone RB6-8C5)	eBioscience	RRID:AB_465314
CD11b/Mac-1::FITC (clone M1/70)	BD PharMingen	RRID:AB_394774
CD45R/B220::FITC (clone RA3-6B2)	BD PharMingen	RRID:AB_394618
FcεR1α::FITC (clone MAR-1)	eBioscience	RRID:AB_465309
CD8a::FITC (clone 53-6.7)	BD PharMingen	RRID:AB_394569
CD11c::FITC (clone N418)	eBioscience	RRID:AB_464941
TER-119::FITC (clone Ter119)	BD PharMingen	RRID:AB_396936
c-Kit::PerCP-Cy5.5 (clone 2B8)	eBioscience	RRID:AB_2534338
Sca-1::PE-Cy7 (clone D7)	eBioscience	RRID:AB_469669
Flt3::PE (clone A2F10)	eBioscience	RRID:AB_465859
CD34::eFluor660 (clone RAM34)	eBioscience	RRID:AB_10596826
CD16/32::BV421 (clone 93)	Biolegend	RRID:AB_2650889
Il-7Rα::BV605 (clone A7R34)	Biolegend	RRID:AB_2572047
CD3e::APC (clone 145-2C11)	eBioscience	RRID:AB_469315
CD4::BV421 (clone H129.19)	BD PharMingen	RRID:AB_2739796
CD8a::PE (clone 53-6.7)	BD PharMingen	RRID:AB_394571
CD45R/B220::PerCP-Cy5.5 (clone RA3-6B2)	BD PharMingen	RRID:AB_394457
Ly-6G/Gr-1::FITC (clone RB6-8C5)	eBioscience	RRID:AB_465315
CD11b/Mac-1::BV605 (clone M1/70)	BD PharMingen	RRID:AB_2737951
TER-119::PE-Cy7 (clone Ter119)	BD PharMingen	RRID:AB_396898
B220::PE (clone RA3-6B2)	BD PharMingen	RRID:AB_394620
IgM::APC (clone II/41)	BD PharMingen	RRID:AB_398464
CD3e::PE (clone 145-2C11)	eBioscience	RRID:AB_465498
Ly-6G/Gr-1::PE (clone RB6-8C5)	eBioscience	RRID:AB_466047
CD11b/Mac-1::PE (clone M1/70)	BD PharMingen	RRID:AB_394775
CD45R/B220::PE (clone RA3-6B2)	BD PharMingen	RRID:AB_394620
TER-119::PE (clone Ter119)	BD PharMingen	RRID:AB_394986
CD8a::APC (clone 53-6.7)	BD PharMingen	RRID:AB_398527
CD44::PerCP-Cy5.5 (clone IM7)	eBioscience	RRID:AB_925746
CD25::PE-Cy7 (clone PC61.5)	eBioscience	RRID:AB_469608
CD4::FITC (clone H129.19)	Biolegend	RRID:AB_1279237
CD45R/B220::PerCP-Cy5.5 (clone RA3-6B2)	Biolegend	RRID:AB_893354
Gr-1::PE (clone 1A8)	BD PharMingen	RRID:AB_394208
Mac-1::PE (clone M1/70)	Biolegend	RRID:AB_312791

(*Continued on next page*)

Continued

REAGENT or RESOURCE	SOURCE	IDENTIFIER
CD45.1::BV421 (clone A20)	Biolegend	RRID:AB_2562563
CD45.2::APC (clone 104)	Biolegend	RRID:AB_389211
TER-119::PE-Cy7 (clone Ter119)	Biolegend	RRID:AB_2281408
CD45.1::BV605 (clone A20)	Biolegend	RRID:AB_2562565
CD71::FITC (clone R17217.1.4)	eBioscience	RRID:AB_465124
BrdU::FITC (clone B44)	BD PharMingen	RRID:AB_400327
Goat-anti-mouse::AF488	Invitrogen	RRID:AB_2534069
rabbit polyclonal anti-ADH5	Proteintech	RRID:AB_593422
rabbit polyclonal anti-ALDH2	Proteintech	RRID:AB_2224185
mouse monoclonal anti-DDDDK tag (anti-FLAG)	MBL	RRID:AB_2687989
Biological Samples		
Patient cell lines	This study	N/A
Chemicals, Peptides, and Recombinant Proteins		
O-(2,3,4,5,6-pentafluorobenzyl)hydroxylamine	Sigma-Aldrich	Cat#76735
Formaldehyde solution	Thermo Fisher Pierce	Cat#28906
2′-Deoxy-N^2-methylguanosine	Carbosynth	Cat#ND06236
^{15}N-deoxyguanosine	Cambridge Isotope Laboratories	Cat#NLM-3899-CA-PK
BrdU slow release pellets, 50 mg/21 days	Innovative Research of America	Cat#N-231
Critical Commercial Assays		
Lineage Depletion Kit	StemCell Technologies, Inc.	Cat#19816A
Methocult GF M3434	StemCell Technologies, Inc.	Cat#03434
Deposited Data		
Single-cell transcriptomes of murine HS(P)Cs	This study	GEO: GSE157832
Genome sequencing data from HSPC clones	This study	ENA: PRJEB40375
Patient exome sequencing data	This study	EGA: EGAS00001003809
Experimental Models: Organisms/Strains		
Mouse: *Aldh2*$^{tm1a(EUCOMM)Wtsi}$	EUCOMM	RRID:MGI:5467969
Mouse: *Adh5*^{tm1Stam}	Liu et al., 2004	RRID:MGI:3033876
Mouse: C57BL/6J	The Jackson Laboratory	RRID:IMSR_JAX:000664
Mouse: B6.SJL	Taconic	RRID:IMSR_TAC:b6sjl
Oligonucleotides		
Primer sequences used in this study, see Table S7	This study	N/A
Recombinant DNA		
mmAldh2-pTrcHis-TOPO	This study	N/A
mmAldh2 cDNA	I.M.A.G.E., Source Bioscience	IMAGE ID 3600875
Software and Algorithms		
Scanpy	Wolf et al., 2018	https://github.com/theislab/scanpy
GATK, version 4.1.0	Van der Auwera et al., 2013	RRID:SCR_001876; https://github.com/broadinstitute/gatk/releases
MassHunter GCMS Acquisition, version B.07.05.2479	Agilent	N/A
MassHunter Quantitative Analysis for GCMS, version B.07.01 SP1/Build 7.1.524.1	Agilent	N/A
Prism, version 8	GraphPad	N/A

RESOURCE AVAILABILITY

Lead Contact

Further information and requests for resources and reagents should be directed to KJ Patel, kjp@mrc-lmb.cam.ac.uk.

CellPress
OPEN ACCESS

Materials Availability

This study did not generate new unique reagents; patient-derived cell lines have been deposited at JCRB cell bank and can be obtained from there.

Data and Code Availability

Single-cell RNA sequencing data have been deposited in Gene Expression Omnibus (accession GEO: GSE157832); genome sequencing data from HSPC clones have been deposited at the European Nucleotide Archive (accession ENA: PRJEB40375). Patient exome sequencing data have been deposited at the European Genome-Phenome Archive (accession EGA: EGAS00001003809). All other data and code are available upon reasonable request from the authors.

EXPERIMENTAL MODEL AND SUBJECT DETAILS

Mice

All animals were maintained in specific pathogen-free conditions. In individual experiments mice were matched for gender and age. All animal experiments undertaken in this study were done so with the approval of the Animal Welfare Ethical Review Body and under project license authority granted by the UK Home Office. *Aldh2*$^{-/-}$*Adh5*$^{-/-}$ mice were generated and bred into a C57BL/6J background. To this end, the previously reported *Aldh2* allele (*Aldh2*$^{tm1a(EUCOMM)Wtsi}$; MGI ID: 4431566, EUCOMM) was intercrossed with the previously reported *Adh5* allele (*Adh5*^{tm1Stam}; MGI ID: 3033711, a gift from Dr. Linmin Liu (Liu et al., 2004)). Some littermate *Adh5*$^{+/-}$ *Aldh2*$^{+/-}$ animals are included with the wild-type controls; they showed no noticeable difference from independently derived *Adh5*$^{+/+}$ *Aldh2*$^{+/+}$ animals.

For competitive repopulation experiments, C57BL/6Ola mice were intercrossed with B6.SJL (CD45.1) mice (Taconic) to generate CD45.1/CD45.2 recipients.

Research subjects

The overall research plan was approved by the Ethical Committee of Kyoto University and other participating institutions. Written informed consent was obtained from all subjects examined. Subjects' age and sex are indicated in Table 1. The patient-derived cell cultures and information including SCE levels were originally deposited by Dr. Masao S. Sasaki (formerly at the Radiation Biology Center, Kyoto University) to the JCRB Cell Bank, and were provided to us with the consent of Dr. Sasaki. Genomic DNA was isolated from primary fibroblast cultures (P1-P5) or a buccal swab (P6) or peripheral blood mononuclear cells (family members and healthy PHA-blast donors) using Gentra Puregene kits. Chromosome breakage tests were carried out with MMC 0.02 μg per ml (50-72h) or with DEB 0.1 μg per ml (48h) as previously described (Sasaki and Tonomura, 1973; Yabe et al., 2007). Whole exome sequencing (WES) of genomic DNA and subsequent processing were done as described previously (Muramatsu et al., 2017). *ALDH2* genotyping was done with Taqman PCR as described (Hira et al., 2013). Genome PCR and Sanger sequencing were done according to the standard procedure with primer sequences described in Table S7. Subjects in the HERPACC project were recruited between January 2001 and December 2005 from the Hospital-based Epidemiologic Research Program at Aichi Cancer Center (HERPACC)-2. The framework of HERPACC-2 has been described elsewhere (Hamajima et al., 2001). Non-cancer controls (n = 4206) were randomly selected from the HERPACC-2 database. DNA of each subject was extracted from the buffy coat fraction with a QIAamp DNA Blood Mini Kit (QIAGEN). Genotyping of *ALDH2* (rs671) and three *ADH5* variants (c.966delG, c.G832C, and c.564+1G > A) was conducted using TaqMan Assays with a 7500 Real-Time PCR System (Applied Biosystems).

METHOD DETAILS

Blood counts

Total blood was collected in K_3EDTA MiniCollect tubes (Greiner bio-one) and analyzed on a scil VetABC Plus+ blood counter (Horiba).

Histology and Immunohistochemistry

Dorsal skin was embedded in OCT medium and frozen on dry ice in a 2-methylbutane bath. Skin was cryosectioned at 20 μM at −30°C, and fixed in 10% formalin, then stained with oil red O and hematoxylin. Spleens and femurs were fixed in 10% neutral-buffered formalin for a minimum of 24 hours. Femurs were decalcified. Tissues were embedded in paraffin. After sectioning at 4 μm, tissues were deparaffinized and rehydrated using standard histological methods. Bone marrow was stained with anti-B220 antibody (R&D Systems, MAB1217, 1:500) for IHC. Spleens were stained with anti-B220 antibody and anti-CD3.

Single cell RNA-seq

The femurs, tibiae, iliac crest, humeri, and vertebrae of 6-16 weeks old mice were crushed, washed with 10 mL of PBS supplemented with 2% heat-inactivated FBS, and strained through 70-μm meshes. Cell suspension was depleted of red blood cells by ammonium chloride lysis (STEMCELL Technologies), and stained with the lineage depletion kit (19816A, STEMCELL Technologies) following the manufacturer's instructions and passed through magnetic columns. Lineage-depleted cells were resuspended in 100 μl of PBS supplemented with 2% FCS containing the following antibodies against: c-Kit (APC-Cy7, clone 2B8, 105826, Biolegend), Sca-1 (BV421,

clone D7, 108128, Biolegend), CD45 (FITC, clone 30-F11, 103108, Biolegend), Flt3 (PE, clone A2F10, 135306, Biolegend) and Il-7Rα (BV605, clone A7R34, 135041, Biolegend). Cells were incubated at 4°C for 30 minutes in the dark, washed, and resuspended in 100 μl of PBS supplemented with 2% FCS containing streptavidin (BV510, 405234, Biolegend). Cells were further incubated at 4°C for 15 minutes, washed and resuspended in 500 μl of PBS supplemented with 2% FCS containing 0.5 μl 7AAD (A1310, Life Technologies). Cells (lineage$^-$ c-Kit$^+$ population, and lineage$^-$ c-Kit^{lo} Sca-1$^+$ population) were bulk sorted using a Becton Dickinson Influx sorter.

Single cell expression analysis

Sorted cells were processed using 10x Chromium (10x Genomics, Pleasanton, CA) according to the manufacturer's protocol. Sample demultiplexing, barcodes processing, and gene counting was performed using the count commands from the Cell Ranger v1.3 pipeline. After Cell Ranger processing, each sample was filtered for potential doublets by simulating synthetic doublets from pairs of scRNaseq profiles and assigning scores based on a k-nearest-neighbor classifier on PCA transformed data. The 4.5% of cells with the highest doublets scores from each sample were removed from further analysis, respectively. Cells with > 10% of unique molecular identifier (UMI) counts mapping to mitochondrial genes, expressing fewer than 1200 genes, or with total number of UMI counts further than 3 standard deviations from the mean were excluded. After quality control, 8204 cells from WT, 7709 cells from *Aldh2*$^{-/-}$, 8401 cells from *Adh5*$^{-/-}$, and 9014 cells from *Aldh2*$^{-/-}$*Adh5*$^{-/-}$ were retained for downstream analysis. These cells were then normalized to the same total count. All scRNaseq data was analyzed using the Scanpy Python Module (Wolf et al., 2018). Unsupervised UMAP clustering (Uniform Manifold Approximation and Projection) was carried out using the Louvain algorithm, and cell identity was manually annotated based on the following marker genes: Procr (HSC cluster), Dntt/Flt3 (Lymphoid cluster), Irf8/Ms4a6c (Monocyte cluster), Mpo/Elane/Ctsg (Neutrophil cluster), Itga2b/Pf4/Vwf (Megakaryocyte cluster), Gzmb/Cma3/Mcpt8 (Mast cell cluster), Klf1/Gata1 (Erythroid cluster).

Heatmap Visualization of Adh and Aldh Family Genes

WT cells from an independent experiment were clustered using the Louvain method and annotated based on their gene expression similarity to annotated clusters from previously published Lineage$^-$ c-Kit$^+$ hematopoietic landscapes (Dahlin et al., 2018). The expression of *Adh* and *Aldh* family genes were then plotted on a heatmap grouped by their Louvain clustering. Each column was scaled independently between 0 and 1.

Quantification of HSC numbers across Genotypes

UMAP visualizations were calculated in Scanpy using default parameters. Each cell from the second experiment was mapped to the previously computed Louvain clusters using a KNN classifier in PCA space. Using these assigned clusters, the most immature cluster annotated through HSC-related gene expression markers as 'HSPC' was isolated and the proportion of cells in this cluster belonging to each genotype calculated.

Quantification of HSC state using hscScore

Each single cell transcriptome was scored using the recently published hscScore method (Hamey and Göttgens, 2019). Briefly, the transcriptome of each cell is compared to the transcriptomes of known HSCs using a MLP deep learning model to assign a score representing how likely the cell is to be a true HSC, with a score of 1 representing the most HSC-like cell in the dataset. Violin plots of the hscScore results for the 'HSPC' cluster were created using the Seaborn Python module.

Subclustering of HSPC cluster

Cells identified as belonging to the HSPC cluster were re-clustered and a new UMAP visualization was calculated. Seven clusters were found and a list of genes upregulated in each cluster compared to the union of all other clusters was calculated. The clusters were then annotated based on their differential expression of known HSC-, lineage- or cell cycle-related marker genes such as *Procr, Mllt3, Mettl7a1* (HSC), *Flt3, Dntt* (Lymphoid) and *Mpo, Ctsg, Cdk6* (Myeloid/Cycling).

Cell cycle profiling

Cell cycle assignment of erythroid progenitors was performed following the method established in Tirosh et al. (2016) for scoring cycling cells and implemented with Scanpy. Lists of 43 genes associated with S-phase and 55 genes associated with G2/M phases from Tirosh et al. (2016) were used to quantify the relative expression of these cell-cycle stages compared with a randomly chosen set of reference genes. Cells with high relative expression levels of either program were assigned to be in S-phase or G2/M phase respectively, while cells with no clear expression of either program were assigned to the G1 phase. No cells expressed relatively high levels of both S and G2/M phase programs.

Apoptosis module score

The apoptosis module score for the erythroid progenitors was calculated using the expression values of a set of 298 genes belonging to the 'Intrinsic Apoptotic Signaling Pathway' gene ontology term downloaded from http://www.informatics.jax.org/ (GO:0097193). For each cell, the score was given by

$$\sum_{g} \frac{\ln(x_g + 1)}{n}$$

Where x_g is the normalized expression of a gene g, and n is the size of the geneset.

DNA repair gene expression analysis

For each genotype, the list of DEGs in each cluster was intersected with the list of DNA repair genes belonging to the ‘DNA Repair’ gene ontology term (GO:006281). The resulting number of DNA repair DEGs and their median fold-change in each cluster was calculated and plotted as the size and color of circles respectively using the python module Matplotlib.

Flow cytometry

HSC and progenitor quantification

Bone marrow cells were isolated from femurs, tibiae and iliac crests with PBS supplemented with 2% FCS and strained through 70 μm meshes. Red cells were lysed by resuspending the cells in 10 mL red cell lysis buffer (130-094-183, MACS Miltenyi Biotec) for 10 min at room temperature. After centrifugation, the cell pellet was resuspended in PBS supplemented with 2% FCS and nucleated cells were counted with 3% acetic acid on a Vi-Cell XR cell viability counter (Beckman Coulter). 10×10^6 bone marrow cells were resuspended in 200 μL of PBS supplemented with 2% FCS containing the following antibody solution: FITC-conjugated lineage cocktail with antibodies against CD4 (clone H129.19, BD PharMingen), CD3e (clone 145-2C11, eBioscience), Ly-6G/Gr-1 (clone RB6-8C5, eBioscience), CD11b/Mac-1 (clone M1/70, BD PharMingen), CD45R/ B220 (clone RA3-6B2, BD PharMingen), FcεR1α (clone MAR-1, eBioscience), CD8a (clone 53-6.7, BD PharMingen), CD11c (clone N418, eBioscience), TER-119 (clone Ter119, BD PharMingen); c-Kit (PerCP-Cy5.5, clone 2B8, eBioscience), Sca-1 (PE-Cy7, clone D7, eBioscience), Flt3 (PE, clone A2F10, eBioscience), CD34 (eFluor660, clone RAM34, eBioscience), CD16/32 (BV421, clone 93, BioLegend) and Il-7Rα (BV605, clone A7R34, BioLegend).

Myeloid, erythroid, B and T lymphoid populations

Bone marrow cells (1×10^6) as prepared above were resuspended in 200 μL of PBS supplemented with 2% FCS containing a mature lineage cocktail that consist of antibodies against: CD3e (APC, clone 145-2C11, eBioscience), CD4 (BV421, clone H129.19, BD PharMingen), CD8a (PE, clone 53-6.7, BD PharMingen), CD45R/ B220 (PerCP-Cy5.5, clone RA3-6B2, BD PharMingen), Ly-6G/Gr-1 (FITC, clone RB6-8C5, eBioscience), CD11b/Mac-1 (BV605, clone M1/70, BD PharMingen), TER-119 (PE-Cy7, clone Ter119, BD PharMingen). Spleen cell suspensions in PBS supplemented with 2% FCS were prepared by gently washing and straining whole spleen through a 70 μm mesh. Red cell lysis, cell counting and staining with the mature lineage cocktail were as described above to quantify the myeloid, erythroid, B and T lymphoid populations. Myeloid, B and T lymphoid populations in the peripheral blood were quantified by red cell lysing 100 μl of whole blood with addition of 1 mL of ammonium chloride lysis buffer (155 mM NH_4Cl, 10 mM $KHCO_3$, 0.1 mM Na_2EDTA, pH 7.2), incubated for 10 min at room temperature and washed with 3 mL of PBS supplemented with 2% FCS. Following centrifugation, cells were resuspended in 100 μl PBS supplemented with 2% FCS containing the mature lineage cocktail. Ter-119 was used to exclude unlysed red cells and red cell debris.

B cell maturation in the bone marrow

Bone marrow cells (1×10^6) as prepared above were stained with antibodies against CD45R/B220 (PE, clone RA3-6B2, BD PharMingen) and IgM (APC, clone II/41, BD PharMingen).

Thymic development

A whole thymus was gently washed and strained through a 70 μm mesh to prepare single cell suspensions. 10×10^6 thymic cells were stained in 200 μl PBS supplemented with 2% FCS containing the following antibody solution: PE-conjugated lineage cocktail with antibodies against CD3e (clone 145-2C11, eBioscience), Ly-6G/Gr-1 (clone RB6-8C5, eBioscience), CD11b/Mac-1 (clone M1/70, BD PharMingen), CD45R/ B220 (clone RA3-6B2, BD PharMingen) and TER-119 (clone Ter119, BD PharMingen); CD4 (BV421, clone H129.19, BD PharMingen), CD8a (APC, clone 53-6.7, BD PharMingen), CD44 (PerCP-Cy5.5, clone IM7, eBioscience), CD25 (PE-Cy7, clone PC61.5, eBioscience)

Competitive repopulation assay

This was performed essentially as described previously (Garaycoechea et al., 2018). Briefly, CD45.1$^+$ CD45.2$^+$ recipients were subjected to two doses of 5 Gy whole-body irradiation, three hours apart, before intravenous injection of a cell suspension containing 200 000 nucleated bone marrow cells from donor and 200 000 sex-matched nucleated bone marrow cells from a B6.SJL competitor in 200 μl IMDM. Blood samples were collected at 4-week intervals, red cells lysed and cells stained in 100 μl 2% FBS/PBS containing the following fluorochrome-conjugated antibodies: CD4 (FITC, clone H129.19, Biolegend), CD8 (FITC, clone 53-6.7, BD), B220 (PerCP-Cy5.5, clone RA3-6B2, Biolegend), Gr-1 (PE, clone 1A8, BD), Mac-1 (PE, clone M1/70, Biolegend), CD45.1 (BV421, clone A20, Biolegend), CD45.2 (APC, clone 104, Biolegend), TER-119 (PE-Cy7, clone TER-119, Biolegend). After four months, to determine long-term reconstitution, thymus was stained determine chimerism using the following antibodies: CD3e (PE, clone 145-2C11, eBioscience), B220 (PE, clone RA3-6B2, BD), Gr-1 (PE, clone 1A8, BD), Mac-1 (PE, clone M1/70, Biolegend), TER-119 (PE, clone TER-119, Biolegend), CD4 (BV421, clone RM4-5, Biolegend), CD8a (FITC, clone 53-6.7, BD), CD25 (PE-Cy7, clone PC61.5,

eBioscience), CD44 (PerCP-Cy5.5, clone IM7, Biolegend), CD45.1 (BV605, clone A20, Biolegend), CD45.2 (APC, clone 104, Biolegend). Chimerism in the bone marrow was determined after red cell lysis using a cocktail containing antibodies against lineage antigens (FITC-conjugated, as above), c-Kit (PerCP-Cy5.5, clone 2B8, eBioscience), Sca-1 (PE-Cy7, clone D7, eBioscience), Flt3 (PE, clone A2F10, eBioscience). For enumeration of LT-HSCs, additional antibodies were used against CD34 (eFluor660, clone RAM34, eBioscience), CD45.1 (BV605, clone A20, Biolegend) and CD45.2 (BV421, clone 104, Biolegend). For enumeration of common lymphoid progenitors, Il-7Rα (BV605, clone A7R34, BioLegend), CD45.1 (BV421, clone A20, Biolegend) and CD45.2 (APC, clone 104, Biolegend) were added. Donor-derived chimerism was calculated as the fraction of $CD45.2^{+}CD45.1^{-}$ cells among the sum of $CD45.1^{+}CD45.2^{-}$ and $CD45.2^{+}CD45.1^{-}$ cells in a population.

Micronucleus assay

Micronucleus assay was performed as previously described (Garaycoechea et al., 2018), with blood from mice 2-42 weeks of age (mean 8.7 weeks). 20 µl blood was added to 110 µl solution of heparin in PBS (1000 U ml^{-1}). 120 µl of the blood suspension were added to 1.2 mL methanol at −80°C and stored for at least 12 hours at −80°C. Fixed blood was washed and resuspended in bicarbonate buffer (0.9%(w/v) NaCl, 5.3 mM $NaHCO_3$). A volume corresponding to 2 µl blood in bicarbonate buffer was incubated with 1 µl anti-CD71 (FITC, clone R17217.1.4, eBioscience), 7 µl RNase A (Sigma) in a total volume of 100 µl for 45 min, washed with 1 mL bicarbonate buffer, and resuspended in 500 µl of a 5 µg ml^{-1} solution of propidium iodide in bicarbonate buffer and analyzed without delay.

Sister chromatid exchange assay

This was performed essentially as described previously (Garaycoechea et al., 2018). Mice were implanted with a 50 mg slow-release pellet of BrdU (Innovative Research of America, N-231) Where animals were treated with methanol, they received two doses of 1.5 g kg^{-1} via intraperitoneal injection of a 15% (w/v) solution in saline 16 and 12 hours before analysis. 30 min before femora were collected into ice-cold PBS, mice received an intraperitoneal injection of 100 µl colchicine 0.5% (w/v) in saline (Sigma). Bones were flushed with 10 mL of pre-warmed 75 mM KCl solution and incubated at 37°C for 15 min. Cells were spun down, resuspended in 3 mL Carnoy's fixative (3:1 mixture of methanol:glacial acetic acid) drop-wise by gentle agitation and then topped up to 10 mL fixative. After 30 min at room temperature, cells were spun down, resuspended in 500 µl fixative and stored at −20°C until use. Cells were dropped onto chilled, humidified slides then dried for 1 hour at 60°C. Slides were washed in 2x SSC for 5 min, then stained for 15 min at room temperature with 1 µg ml^{-1} Hoechst 33258 (Thermo Fisher Scientific, H3569) in 2x SSC. Slides were subsequently transferred immersed in 2x SSC and crosslinked for 30 minutes in a Stratalinker crosslinker (Stratagene). Slides were dehydrated by passing through a 70%/96%/100% ethanol, placed in PBS at room temperature for 5 min, DNA was denatured by exposure to 70 mM NaOH for 2 min, then washed 3x 5 min in PBS. The slides were then blocked in blocking buffer (1% BSA, 0.5% Tween-20 in PBS) for 1 h at room temperature, then stained overnight with a FITC-conjugated mouse-anti-BrdU antibody (clone B44, BD PharMingen) diluted 1:1 in 3% BSA, 0.5% Tween-20/PBS. The slides were subsequently washed 3x 5 min in blocking buffer, then stained with AF488-conjugated goat-anti-mouse antibody (Invitrogen, A-11001) diluted 1:500 in blocking buffer for 6 h at room temperature. Slides were washed 3x 15 min in blocking buffer, then stained for 15 min in 5 µg ml^{-1} Hoechst 33342 in (H3570, Thermo Fisher Scientific) in PBS. The slides were then washed 3x 10 min in PBS, once in water for 5 min, and mounted with ProLong Gold Antifade mountant (P36930, Thermo Fisher Scientific).

Methanol treatment for hematopoietic development

Adh5$^{-/-}$ and WT mice aged between 7-12-week-old received intraperitoneal injections with 0.85 g kg^{-1} methanol (99.8% purity, HPLC grade, Fisher scientific) dose on days 1 and 8. Methanol was diluted to 8.5% v/v in saline, and injected at 12.6 mL kg^{-1}. Weight was monitored daily, and on day 10, mice were culled by exposure to CO_2 in rising concentration, followed by cardiac puncture. Blood, spleen, thymus and bones were harvested for analysis of hematopoiesis and lymphocyte development as described above.

ALDH2 biochemistry

Cloning and Expression of Aldh2

Mouse Aldh2 was cloned into a pTrcHis-TOPO expression vector (provided by D. Mochly-Rosen, Stanford University), containing a N-terminal uncleavable 6XHis tag. Mouse *Aldh2* was amplified from full length cDNA (IRAV14-F04, IMAGE I.D. 3600875) using the following primers: 5′-TTATATGCTAGCTCAGCCGCCGCCACCAGCGCGGTG-3′ and 5′-GATGGCGGATCCAAGCTTGCATGATTCTTACGAGTTCTTCTGTGGCACTT-3′ and was cloned into the expression vector using NheI-HindIII sites. This removed the 19 amino acid N-terminal mitochondrial targeting signal peptide of ALDH2. The vector was transformed into *E. coli* BL21 *E. coli* and protein expression induced with 0.5 mM IPTG at 30°C for 5 hours. Cell pellets were harvested, resuspended in Buffer A (containing 25 mM Tris-HCl pH 8.0, 2 mM TCEP, 10% glycerol, 500 mM NaCl). Cells were lysed by the addition of 0.1% sodium deoxycholate, 200 µg ml^{-1} lysozyme with protease inhibitor cocktail (Roche) (50 mL Lysis Buffer per 10 g pellet). The extract was centrifuged at 43,000 × g, for 40 min at 4°C to obtain a soluble fraction for purification.

Purification of ALDH2

Mouse recombinant ALDH2 was purified using a 3-step purification strategy. The soluble fraction was first passed over a 1 mL HisTrap HP column, washed with 40 mM imidazole, and protein eluted with 250 mM imidazole. The pooled fractions were

concentrated before being applied to a Superdex 200), eluted in buffer A (containing 50 mM NaCl). Fractions containing Aldh2 were then diluted three times with a 0 mM NaCl buffer and further purified using an Anion Exchange column (Q15, 3 ml) to yield pure mouse Aldh2 protein. Protein was stored in 50% glycerol at −20°C, or in 10% glycerol at −80°C, and used for enzymatic assays.

Liver mitochondria preparation and ALDH Assay

Mouse liver (0.5 g) was finely minced with scissors and homogenized with 500 μL of homogenization buffer (210 mM mannitol, 70 mM sucrose, 1 mM EDTA, 5 mM MOPS, pH = 7.4 in H_2O) using a Dounce homogenizer. The homogenate was centrifuged at 700 × g for 10 min, the supernatant removed, and centrifuged again at 700 × g for 10 minutes. The supernatant was removed and centrifuged at 7000 × g for 20 min. The pellet was washed in homogenization buffer (centrifuged at 7000 × g for 10 min), then re-suspended in 300 μL of enzyme assay buffer (10 mM DTT, 20% glycerol, 0.1% Triton X-100 and 0.1 M Tris-HCl, pH = 8.0) and centrifuged at 100,000 × g for 30 minutes at 4°C to obtain a clear supernatant. Protein concentration was measured using a NanoDrop.

To perform the ALDH enzymatic activity assay, a 2 mL reaction was set up in a cuvette containing 50 mM NaPPi buffer (pH = 9.0), 2.5 mM NAD^+, 10 mM acetaldehyde and 0.5 mg protein preparation in H_2O. The absorbance at 340 nm was recorded using a Cary 5000 UV-Vis-NIR spectrophotometer, at RT for 300 s without the addition of the acetaldehyde substrate, in order to quench the reaction of endogenous aldehydes. After 3 minutes, acetaldehyde was added, and the absorbance recorded for a further 350 s. To calculate the NADH production in mol/min/mg total protein, we used: Absorbance = ε × c × L, where ε = 6220 M^{-1}, L = path length (1 cm) and c = [NADH] in mol l^{-1}. The assay was adapted from a protocol by D. Mochly-Rosen, Stanford University.

In vitro ALDH Activity assay

To perform ALDH enzymatic activity assays, a 2 mL reaction was set-up in a cuvette containing 50 mM NaPPi buffer (pH = 9.0), 2.5 mM NAD^+, 10 μg recombinant protein and 1 mM subtstrate in H_2O. As soon as the substrate was added, the absorbance at 340 nm was recorded using a Cary 5000 UV-Vis-NIR spectrophotometer, at RT for 300 s. To calculate the NADH production in mol/min/mg total protein, we used: Absorbance = ε × c × L, where ε = 6220 M^{-1}, L = path length (1 cm) and c = [NADH] in mol l^{-1}.

Mouse serum formaldehyde quantification by GC–MS

Mice were euthanized by exposure to CO_2 in rising concentration, followed by cardiac puncture to collect 500 μl - 700 μl whole blood into Microvette 500 Z-gel tubes containing clotting activator (20.1344, Sarstedt). After centrifugation at 10,000 × g for 5 min at room temperature, 100 μl of the serum was transferred to glass crimp top vials (5182-0543, Agilent), followed by addition of internal standards: cyclohexanone (29140, Sigma) and n-Propanol (34871, Sigma) at a final concentration of 1 mg l^{-1} each respectively, and derivatization reagent O-(2,3,4,5,6-pentafluorobenzyl)hydroxylamine (PFBHA, 76735, Sigma) at a final concentration of 60 μg ml^{-1}. The tube was sealed with magnetic crimp caps (5188-5386, Agilent), incubated overnight in the dark at room temperature, and stored at −80°C until analysis by GC–MS. A serum-formaldehyde calibration standard was prepared in parallel with each batch of serum sample collection. Following cardiac blood draw and transfer of the blood into the tube, dilutions of formaldehyde 16% (w/v, 28906, Thermo Fisher Pierce) in PBS were added to the blood sample at final concentrations ranging from 0 μM – 213 μM. Subsequent serum isolation and formaldehyde derivatization was identical to sample preparation as described above.

The mass spectrometer was operated in single ion monitoring mode for the ions m/z 181, 195 and 225 for formaldehyde-PFBHA oxime (retention time 11.47 min) and m/z 181, 195 and 293 for cyclohexanone-PFBHA oxime internal standard (retention time 16.73 min) with m/z 181 used for quantification for both compounds. A dwell time of 200 ms was used for each ion. The transfer line to the mass spectrometer was heated to 220°C, the source temperature was maintained at 230°C and the quadrupole at 150°C. The GC–MS data were acquired using MassHunter GCMS Acquisition B.07.05.2479. For quantification, all analyte integrated peak areas were ratioed to internal standard areas using MassHunter Quantitative Analysis Version B.07.01 SP1/Build 7.1.524.1 for GCMS. The method was calibrated across the range of 0.1 to 5 mg l−1 of formaldehyde: each calibration point was run in triplicate and a demonstrated precision of ≤ 15%.

Synthesis of nucleoside standards

Isotopically labeled nucleosides where purchased from Cambridge Isotope Laboratories, non-labeled from Sigma.

Synthesis of ^{15}N-N^2-MeG and N^2-MeG

In a small glass vial with an air tight screw cap ^{15}N-dG **(1)** or dG (5 mg) **(3)** was dissolved in formaldehyde (1 ml, MeOH free, 5.328 M (16%), Thermo Scientific). After 24 h the reaction was transferred to a round-bottom flask and evaporated twice with water (2 ml). The residue was then dissolved in a solution of NaOAc (pH = 4, 100 mM) with $NaCNBH_3$ or $NaCNBD_3$ respectively (1 ml, 100 mM) and left for another 24 h. The reaction was neutralized with PBS (20 ml) and purified by preparative HPLC on a Varian PrepStar using the following conditions: Buffer A: H_2O Buffer B: MeCN, Gradient: 5%–12.5% Buffer B over 30 min. Column: Waters Atlantis Prep T3, 10 μM, 19 × 250 mm.

The product was obtained at retention time 12.5 min and yield < 1%, and its chemical identity confirmed by ESI mass spectrometry: *^{15}N-N^2-MeG* **(2)** ESI+, 286.097 $[M+H]^+$. Mw = 285.090 $C_{11}H_{14}{}^{15}N_5O_4$. *$N^2$-MeG* **(4)** 283.126 $[M+H]^+$. Mw = 282.119 $C_{11}H_{14}DN_5O_4$.

Determination of the extinction coefficient for N^2-MeG

2′-Deoxy-N^2-methylguanosine (5 mg, Carbosynth) was re-purified on a Thermo Scientific Accela HPLC using the following conditions: Buffer A: H_2O Buffer B: MeCN, Gradient: 5%–20% Buffer B over 30 min. Column: Agilent AdvanceBio Oligonucleotides, 4.6 × 150 mm. The product was evaporated to dryness. The resulting powder was then weighed out using an accurate balance (1-2 mg, Mettler Toledo, XS205). A 0.5 mg ml^{-1} solution was prepared in H_2O. This solution was diluted 1:2 in PBS in quintuplicate,

and the absorbance measured at 260 nm (Nanodrop). Using the Beer-Lambert law the extinction coefficient for N^2-MeG was calculated to be 9277 l mol^{-1} cm^{-1}. The same value was used for ^{15}N-N^2-MeG and N^2-MeG.

Sample preparation for determination of N^2-MeG in DNA

Organs were snap frozen and stored at −80°C until analysis. 10-30 mg of tissue was cut and lysed in a 2 mL Eppendorf, using 733 μL of Puregene cell lysis solution (QIAGEN), 4 μL of proteinase K (Fisher BioReagents) and a 7 mm stainless steel metal ball (QIAGEN). Samples where homogenized in a tissue lyser (QIAGEN/Retsch) for 3 min at 30 Hz, then incubated at 37°C for 30 min, 600 rpm. Then 4 μL of RNase A solution (QIAGEN) were added, vortexed and incubated at 37°C for 1 h at 600 rpm.

The supernatant was transferred to a new tube (1.5 ml) and cooled on ice for 1 min. Then 266 μL protein precipitation solution (QIAGEN, Puregene) and vortexed briefly, cooled on ice for 5 min, spun 21,300 × g, 3 min. The supernatant was transferred into a fresh tube containing 600 μL isopropanol, mixed by inversion 10 × and left at RT for 5 min for the DNA to precipitate. DNA was pelleted by spinning at 21,300 × g for 2 min. The supernatant was discarded and the DNA pellet washed with 600 μL of 70% ethanol, spun at 21,300 × g, 2 min. Again the supernatant was discarded and the pellet left to air-dry for 5 mins before dissolving the pellet by addition of 500 μL of 50 mM, $NaCNBD_3$ in 200 mM NaOAc (pH = 5.2), and dissolved and reacted for 24 h at RT at 1000 RPM in an Eppendorf Thermomixer.

DNA was precipitated out of the $NaCNBD_3$ solution by addition of 900 μl isopropanol, spun at 21,300 × g, 5 min and the supernatant discarded. This step was performed twice and the pellet left to air dry. The DNA was dissolved in 150 μl of ultra-pure water (Romil) and quantified by nanodrop.

DNA was digested in a total volume of 100 μl in reactions containing 5000 ng DNA, 2 U shrimp alkaline phosphatase, (New England Biolabs), 0.004 U snake venom phosphodiesterase I (Sigma, P3243) and 10 U DNase I (Roche) in 1 × DNase I digestion buffer.

Also added to the digest were the internal standards ^{15}N-N^2-MeG and ^{15}N-dA. For standard curve generation a non-reduced sample of genomic liver DNA from a WT mouse was used and the standards 2′-deoxyadenosine (dA) and N^2-MeG added at various concentrations. The range of the standard curves was as follows: 0.24 to 100 fmol for N^2-MeG, 8.5 to 272 nmol for dA. The curves contained 6 points plus a zero control. The response ratio (non-labeled to labeled spike) was plotted versus the amount of non-labeled spike injected onto to the column.

After an overnight digest (> 16 h) samples were filtered with a 2000 MWCO Vivacon® 500 (Sartorius), 40 min, 16000 × g. Samples where then transferred to a MS vial and analyzed.

Online LC-MS^2 determination of N^2-MeG in DNA digests

Samples were analyzed on TSQ Altis Triple Quadrupole Mass Spectrometer in selected reaction monitoring mode (SRM) interfaced to an UltiMate 3000 uHPLC and. The uHPLC was fitted with a nanoEase M/Z Symmetry C18 Trap Column, 100Å, 5 μm, 180 μm × 20 mm (Waters) at RT and a reversed phase EASY-Spray HPLC analytical column (2 μm particle size, 75 μm × 250 mm, Thermo Fisher Scientific) connected to an EASY-Spray source at 35°C.

10 μl of sample (500 ng of digested DNA) was injected per run using a 10 μl sample loop and the full loop inject mode. Buffers used were from Romil and of Ultra LC standard. Buffer A: H_2O (0.1% acetic acid), buffer B MeCN (0.1% acetic acid). The gradient was 0-2.5 min – 1% B, 22 min – 45% B, 23.5 min – 99% B. This was followed by 2 wash pulses (1%–99% B) and equilibration to 1% B (45 min total run time). The trap column was held at a constant 1% B and switching from the trap to the main column occurred at 1 min 24 s and back at 40 min.

Mass spectrometry conditions were as follows: source voltage of 2300V in positive ionisation mode; ion transfer tube temperature 250°C, CID gas pressure 2 mbar, scan widths for Q1 and Q3 at 0.7 *m/z.* Dwell times were 100 ms for N^2-MeG and ^{15}N-N^2-MeG, 10 ms for dA. Collision energy voltage and RF voltage were optimized with authentic standards using the vendor-provided tune software for each fragment in the SRM, however the dA parameters were reduced to 10% of the optimal value due to their high abundance and consequently high ion current.

Detection of acetaldehyde mono-adduct N^2-EtdG by MS

The determination was performed as described previously (Moeller et al., 2013) and performed in the Swenberg laboratory. DNA was isolated using a NucleoBond DNA isolation kit, with small modifications. DNA was then reduced and digested as described previously (Yu et al., 2015). Following digestion, hydrolysed DNA was filtered and injected onto an Agilent 1200 HPLC fraction collection system equipped with a diode-array detector. dG and N^2-EtdG were separated and eluted. The amounts of dG were quantified according to the UV peak area with a calibration curve. The amounts of N^2-EtdG were detected and quantified with a calibration curve on an AB SCIEX Triple Quad 6500 mass spectrometer interfaced with an Eksigent nanoLC Ultra 2D system. The internal standard ^{15}N-N^2-EtdG was synthesized by the Swenberg lab. Chemicals were from Sigma.

Genome sequencing of HSPC colonies

Total bone marrow was diluted in X-Vivo 20 (Lonza) supplemented with 5% (v/v) BIT9500 (Stem Cell Technologies), 10 ng ml^{-1} IL-3, 10 ng ml^{-1} IL-6, 50 ng ml^{-1} SCF and 50 ng ml^{-1} TPO (all Peprotech), and 1 volume of cells added to 10 volumes of Methocult GF M3434 (Stem Cell Technologies) semisolid medium. After 2 weeks, colonies were transferred to liquid culture in supplemented X-Vivo 20 for a further week. DNA from liquid cultures and mouse brain cortex as germline reference was extracted using Zymo Quick-gDNA

microprep kit as per the manufacturer's instructions. DNA was quantified fluorimetrically using AccuBlue High Sensitivity dsDNA Quantitation Kit (Biotium) and libraries prepared using NEBNext Ultra II FS DNA Library Preparation kit with unique dual indices (New England Biolabs) according to the manufacturer's recommendation. Libraries were size-selected to peak around 500 bp, quality-controlled on Agilent Bioanalyzer 2100 and quantified by qPCR using Kapa library quantification kit for Illumina (Roche). Sequencing was performed as 150 bp paired end on NovaSeq S2 (Illumina). Raw sequence data was converted to unsorted BAM format and then fed into the Genome Analysis Toolkit v. 4.1.0 using the best practice pipelines for data preprocessing and somatic variant discovery (Van der Auwera et al., 2013). Alignment was performed against the Illumina mm10 reference genome using a set of single-nucleotide polymorphisms and indels from the Sanger Mouse Genome project as input for base quality score recalibration.

Mutect2 was invoked using matched brain sample as normal reference genome. Final passing variants were filtered to exclude multiallelic sites and sites identified by Mutect2 as normal_artifact, and further restricted to sites with coverage depth > = 20 and variant allele frequency > = 0.3 to limit the analysis to clonal mutations with good confidence. As a quality control step, using the same settings we asked Mutect2 to call mutations in the brain against matched HSPC genomes, but found none passing filters indicating non-clonal origin of the brain tissue.

Patient-derived cell culture and transfection

The patient-derived primary fibroblasts (JCRB Cell Bank, Osaka, Japan) and the 48BR cell lines were cultured in RPMI1640 supplemented with 20% FBS (GIBCO).

SCE assay on patient-derived cells

Peripheral blood mononuclear cells were separated by density gradient centrifugation and stimulated with 5 μg/ml phytohemagglutinin (PHA) (Sigma) in RPMI 1640 (Nacalai Tesque) supplemented with 10% FBS (GIBCO). The staining of metaphase spreads for the quantification of SCEs was performed according to a published protocol (Sonoda et al., 1999). For BrdU labeling, cells were cultured in the presence of 5 μM BrdU for 16 to 18 h (two cell cycle periods) and pulsed with 0.1 μg/ml Colcemid for the last 2 h. Harvested cells were treated with 75 mM KCl for 30 min and subsequently fixed with methanol: acetic acid (3:1) for 40 min. Cells were dropped onto wet (50% ethanol) glass slides and dried on a 42°C plate. Dried slides were incubated with 10 μg/ml Hoechst 33258 in phosphate buffer (pH 6.8) for 20 min, followed by rinsing with MacIlvaine solution (164 mM Na_2HPO_4, 16 mM citric acid, pH 7.0). Slides were irradiated with a black light (352 nm) for 1 h and incubated in 2 × SSC (0.3 M NaCl plus 0.3 M sodium citrate) solution at 62°C for 1 h before staining with 3% Giemsa solution (pH 6.8) and subsequent microscopy.

Immunoprecipitation and western blotting of patient-derived cells

Cells were washed once with PBS and lysed in NETN buffer (150 mM NaCl, 0.5 mM EDTA, 20 mM Tris-HCl pH = 8.0, 0.5% NP-40) supplemented with protease inhibitor cocktail (Roche) and 25 unit/mL Benzonase (Millipore) on ice for 30 min. Lysates were then briefly sonicated and centrifuged at 17,800 g for 10 min at 4°C. GFP-tagged proteins were captured using anti-GFP magnetic beads (Sigma) at 4°C, washed five times with NETN buffer, and eluted by adding 1 × Laemmli sample buffer and boiling. Samples were separated by SDS-PAGE, transferred to a PVDF membrane, and analyzed by western blotting. The antibodies used were rabbit polyclonal anti-ADH5 (Proteintech); rabbit polyclonal anti-ALDH2 (Proteintech), mouse monoclonal anti-DDDDK tag (anti-FLAG) (MBL).

QUANTIFICATION AND STATISTICAL ANALYSIS

Sample number (n) indicates the number of independent biological samples in each experiment and are indicated in figure legends or methods. Unless otherwise stated in the figure legends, data are shown as the mean ± SEM. Unless otherwise stated, statistical significance was assessed using two-tailed Mann-Whitney *U* test. Analysis was performed using GraphPad Prism (Version 8).

Molecular Cell, Volume *80*

Supplemental Information

Two Aldehyde Clearance Systems Are Essential to Prevent Lethal Formaldehyde Accumulation in Mice and Humans

Felix A. Dingler, Meng Wang, Anfeng Mu, Christopher L. Millington, Nina Oberbeck, Sam Watcham, Lucas B. Pontel, Ashley N. Kamimae-Lanning, Frederic Langevin, Camille Nadler, Rebecca L. Cordell, Paul S. Monks, Rui Yu, Nicola K. Wilson, Asuka Hira, Kenichi Yoshida, Minako Mori, Yusuke Okamoto, Yusuke Okuno, Hideki Muramatsu, Yuichi Shiraishi, Masayuki Kobayashi, Toshinori Moriguchi, Tomoo Osumi, Motohiro Kato, Satoru Miyano, Etsuro Ito, Seiji Kojima, Hiromasa Yabe, Miharu Yabe, Keitaro Matsuo, Seishi Ogawa, Berthold Göttgens, Michael R.G. Hodskinson, Minoru Takata, and Ketan J. Patel

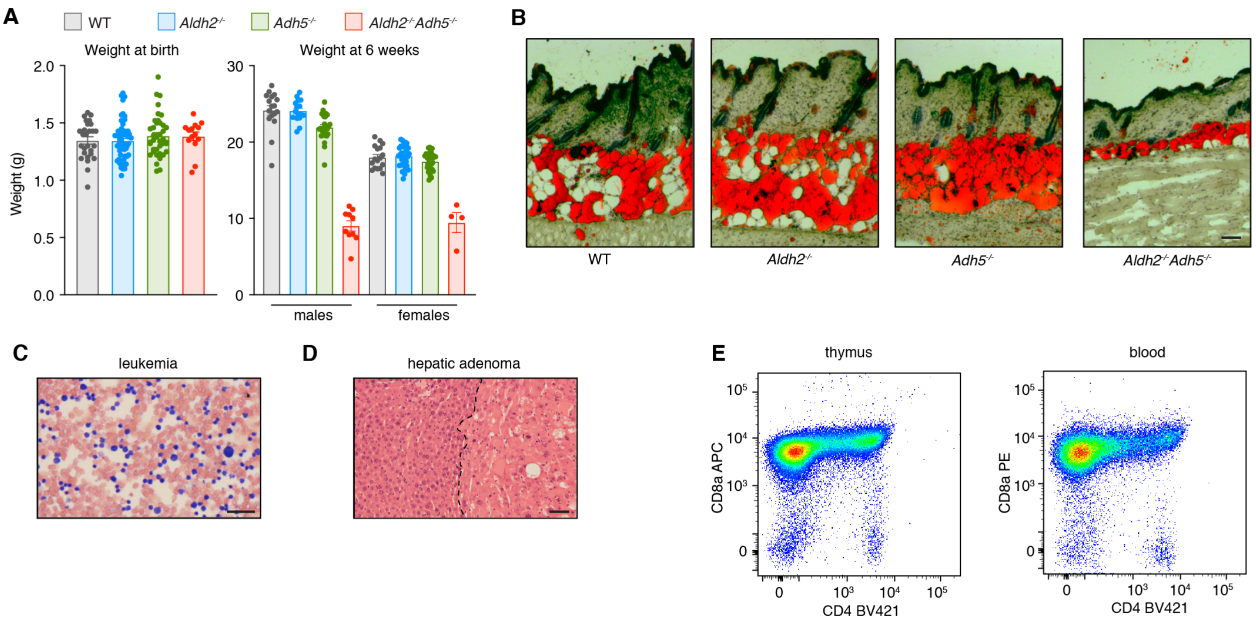

Figure S1, relating to Figure 1. Postnatal lethality of *Aldh2$^{-/-}$Adh5$^{-/-}$* mice

(A) Mouse weight at birth (*n* = 27, 57, 40, 14; left to right) and at 6 weeks (*n* = 17, 16, 26, 10, 17, 33, 28, 4; left to right). **(B)** Oil red O staining of subcutaneous fat in dorsal skin. Scale bar: 100 μm. **(C)** Blood film of *Aldh2$^{-/-}$Adh5$^{-/-}$* mouse which died at 38 weeks showing leukemia. Scale bar: 50 μm. **(D)** Hepatic adenoma in same mouse. Scale bar: 100 μm. **(E)** $CD3^-$ $CD8^+$ T cell leukemia seen in a 42-week-old mouse.

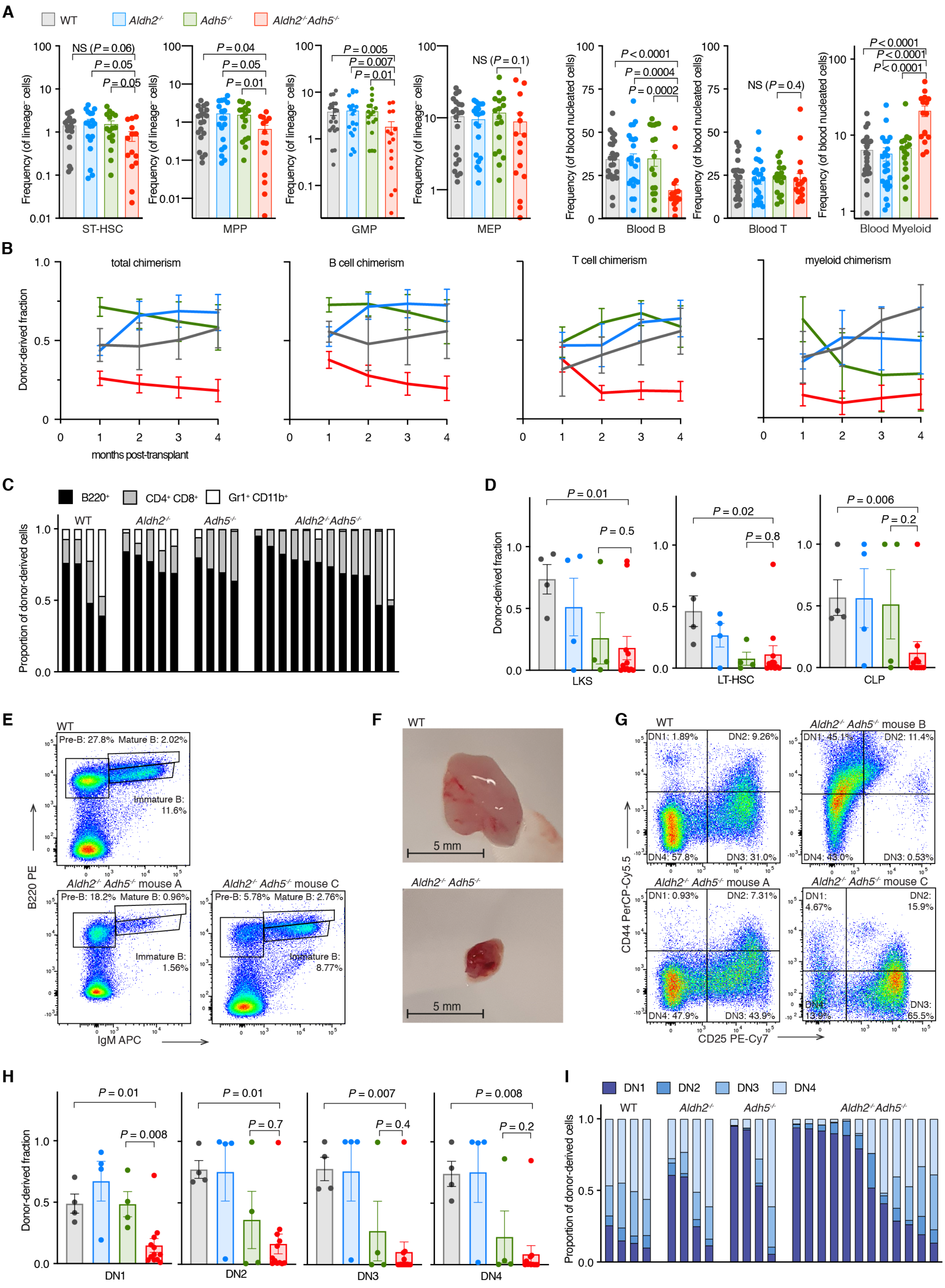

Figure S2, relating to Figure 2 and 3. Disrupted hematopoiesis and lymphoid development in *Aldh2-/-Adh5-/-* mice.

(A) Quantification of bone marrow ST-HSC, MPP, GMP and MEP populations, and blood B, T lymphocyte, and myeloid (CD11b+ Gr-1+) populations in *Aldh2-/-Adh5-/-* mice with age matched controls (data shown as mean ± SEM, *n* = 24, 20, 17 and 17 mice,

left to right). **(B)** Long-term competitive reconstitution experiment showing serial blood samples of transplanted mice analyzed for contribution to B220+ (B cell), CD4+/CD8+ (T cell) and Gr1+/Mac-1+ (myeloid) compartment. Fraction of donor-derived cells, donor/(donor+competitor), shown as mean ± SEM, *n* = 4, 5, 4, 12 recipients for *WT, Aldh2*$^{-/-}$*, Adh5*$^{-/-}$*, Aldh2*$^{-/-}$ *Adh5*$^{-/-}$ donors respectively. **(C)** Output bias of donor bone marrow assessed at 4 months. **(D)** Contribution to LKS, long-term HSC, and common lymphoid progenitor population at 4 months (*n* = 4, 4, 4, 12). **(E)** Flow cytometry plots of bone marrow B cell populations showing the variation observed in *Aldh2*$^{-/-}$*Adh5*$^{-/-}$ mice. Mouse A shows preferential loss of immature and mature B cells, compared to loss of pre-B cells in mouse C. **(F)** Macroscopic picture of thymus in a WT and an *Aldh2*$^{-/-}$*Adh5*$^{-/-}$ mouse. **(G)** Flow cytometry plots of DN populations defined by CD44 and CD25 expression from three *Aldh2*$^{-/-}$*Adh5*$^{-/-}$ mice aged 2-4 weeks. Mouse A shows DN population pattern comparable to WT thymus, mouse B thymus shows significant depletion of DN2 and 3 populations, whereas mouse C thymus shows expansion of DN2 and 3 populations. **(H)** Fraction of donor-derived cells amongst donor- or competitor-derived cells in the double-negative fraction of the thymus. Data shown as mean ± SEM, *n* = 4, 4, 4 and 12 recipients. **(I)** Distribution of donor-derived cells amongst DN fractions in thymus of competitively repopulated recipients (*n* = 4, 4, 4, 12).

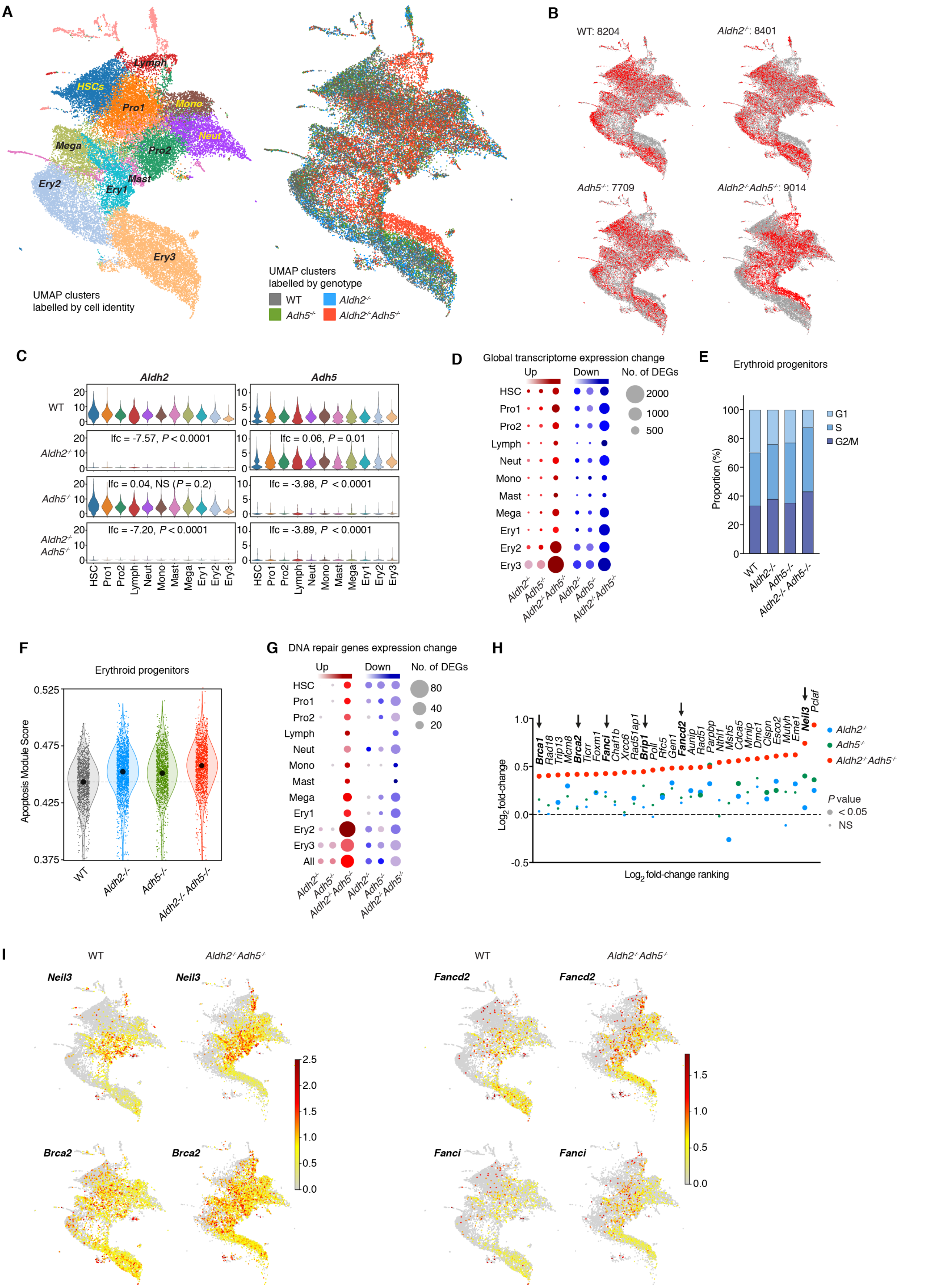

Figure S3, relating to Figures 2, 3 and 4. Single cell RNAseq analysis of hematopoietic stem and progenitor cells in *Aldh2$^{-/-}$Adh5$^{-/-}$* mice.

(A) Two-dimensional visualization by UMAP of bone marrow (Lin$^-$ c-Kit$^+$ and Lin$^-$ Sca-1$^+$ c-Kit^{lo}) transcriptomes combined from all 4 genotypes. Each dot represents a

transcriptome from a single cell. On the left the clusters are colored by HSC or lineage-primed progenitor identity assigned by expression of lineage-specific marker genes. On the right transcriptomes are colored by genotype to highlight variation in distribution between the *Aldh2*$^{-/-}$*Adh5*$^{-/-}$ and controls across the UMAP clusters. **(B)** Transcriptomes of individual genotypes highlighted in red over the background of all genotypes in grey. **(C)** Ensemble of violin plots showing normalized gene expression score for *Aldh2* and *Adh5* (number of UMIs per 10000) for each cluster and genotype. **(D)** Analysis of differentially expressed genes, circle size denoting number of genes passing filter and color intensity reflecting median fold change. **(E)** Cell cycle analysis of erythroid progenitors based on transcriptome profile. **(F)** Apoptosis module score for erythroid progenitors. **(G)** Analysis of differentially expressed genes annotated as DNA repair gene in gene ontology, circle size denoting number of genes passing filter and color intensity reflecting median fold change. **(H)** Top differentially expressed DNA repair genes by genotype. Genes implicated in crosslink repair are highlighted with arrows. **(I)** Select examples of gene expression plotted on the UMAP landscape of WT and *Aldh2*$^{-/-}$*Adh5*$^{-/-}$ hematopoietic stem and progenitor cells. See also Tables S3, S4, and S6.

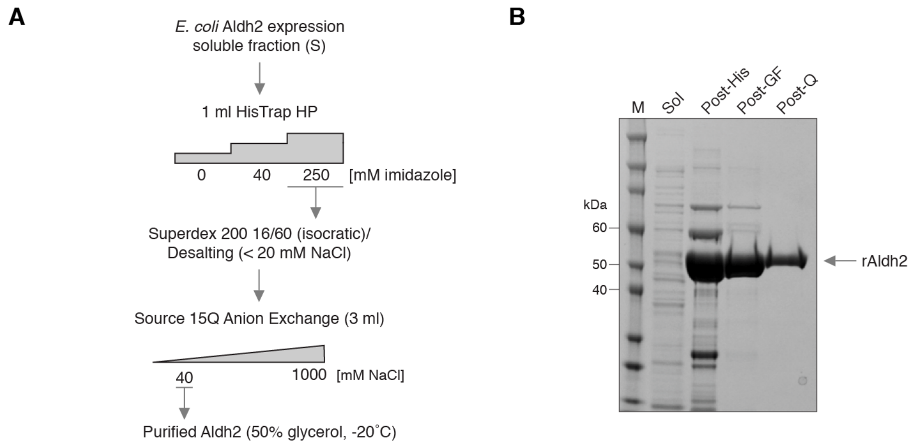

Figure S4, relating to Figure 4. Purification of recombinant mouse ALDH2

(A) Scheme of purification strategy. **(B)** Coomassie stained SDS-PAGE of fractions: M, size marker; Sol, soluble fraction; Post-His, pooled chelate eluate; Post-GF, pooled gel filtration eluate; post-Q, Q anion exchange eluate.

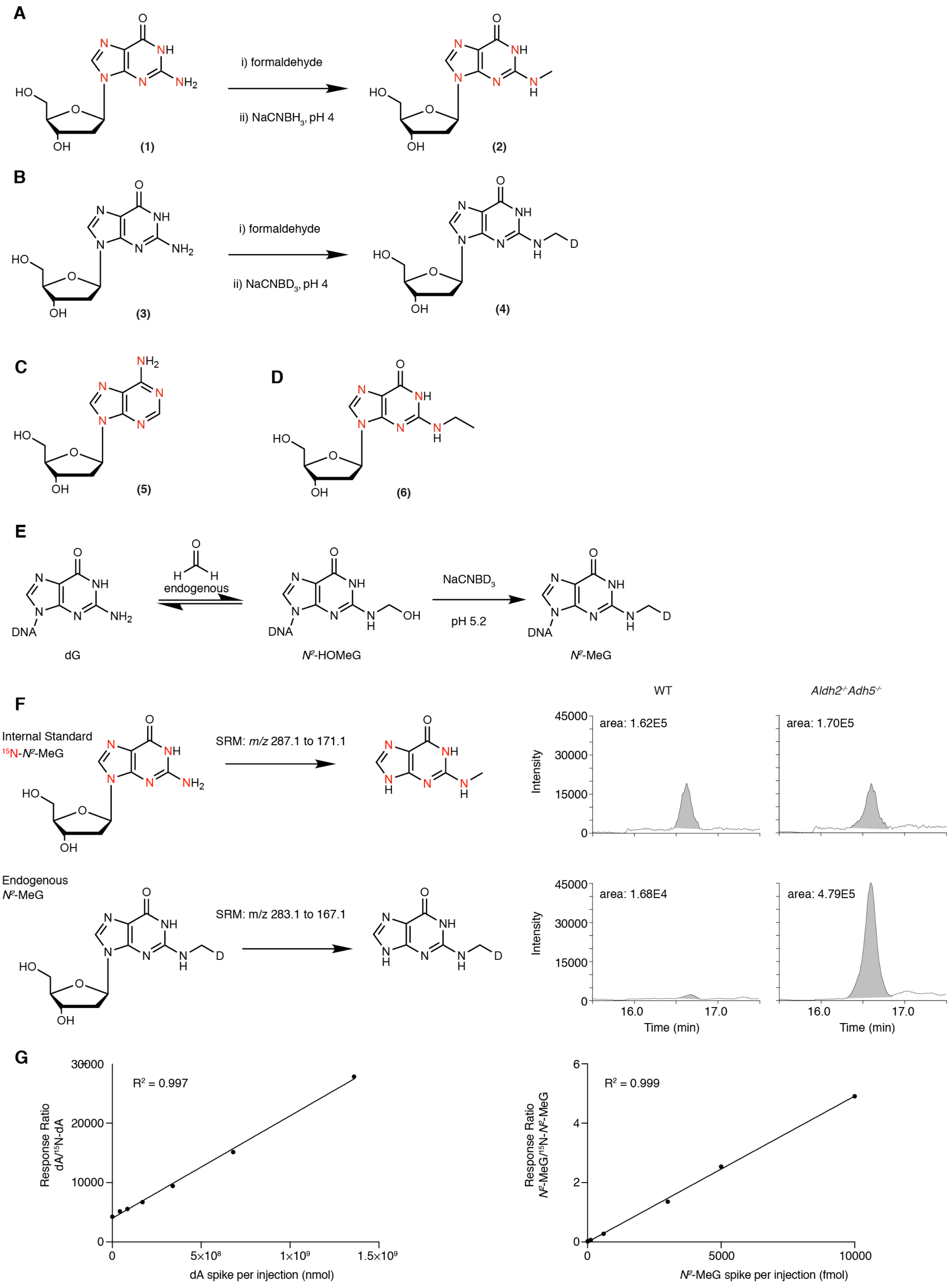

Figure S5, relating to Figure 5. Chemical synthesis of formaldehyde-DNA adduct standards and quantification of *N*²-MeG via mass spectrometry.

(A) Reaction to make ^{15}N-*N*²-MeG (red letter = ^{15}N): i) formaldehyde, ii) $NaCNBH_3$, pH4. **(B)** Reaction to make *N*²-MeG, i) formaldehyde, ii) $NaCNBD_3$, pH4. **(C)** Structure of the

^{15}N-dA standard used for *in situ* quantification of total injected digested DNA per MS run (red letter = ^{15}N). **(D)** Structure of the ^{15}N-*N*^{2}-EtG standard used in this study (red letter = ^{15}N). **(E)** Reaction scheme for the stabilization of formaldehyde mono-adducts on genomic DNA. Endogenous formaldehyde can react at the *N*^{2}-position of guanine to form the hydroxymethyl mono-adduct, however this reaction is reversable. We used $NaCNBD_3$ to reduce the *N*^{2}-HOMeG adduct to the stable *N*^{2}-MeG (D omitted for clarity). **(F)** Left: MS/MS transitions for the internal standard and endogenous *N*^{2}-MeG used in the SRM method. Right: representative and corresponding MS traces for WT and *Aldh2*$^{-/-}$*Adh5*$^{-/-}$ mouse tissue (red letter = ^{15}N). **(G)** Standard curve examples for the MS/MS data presented in this paper. Fresh standard curves were prepared with every batch of digested genomic DNA samples.

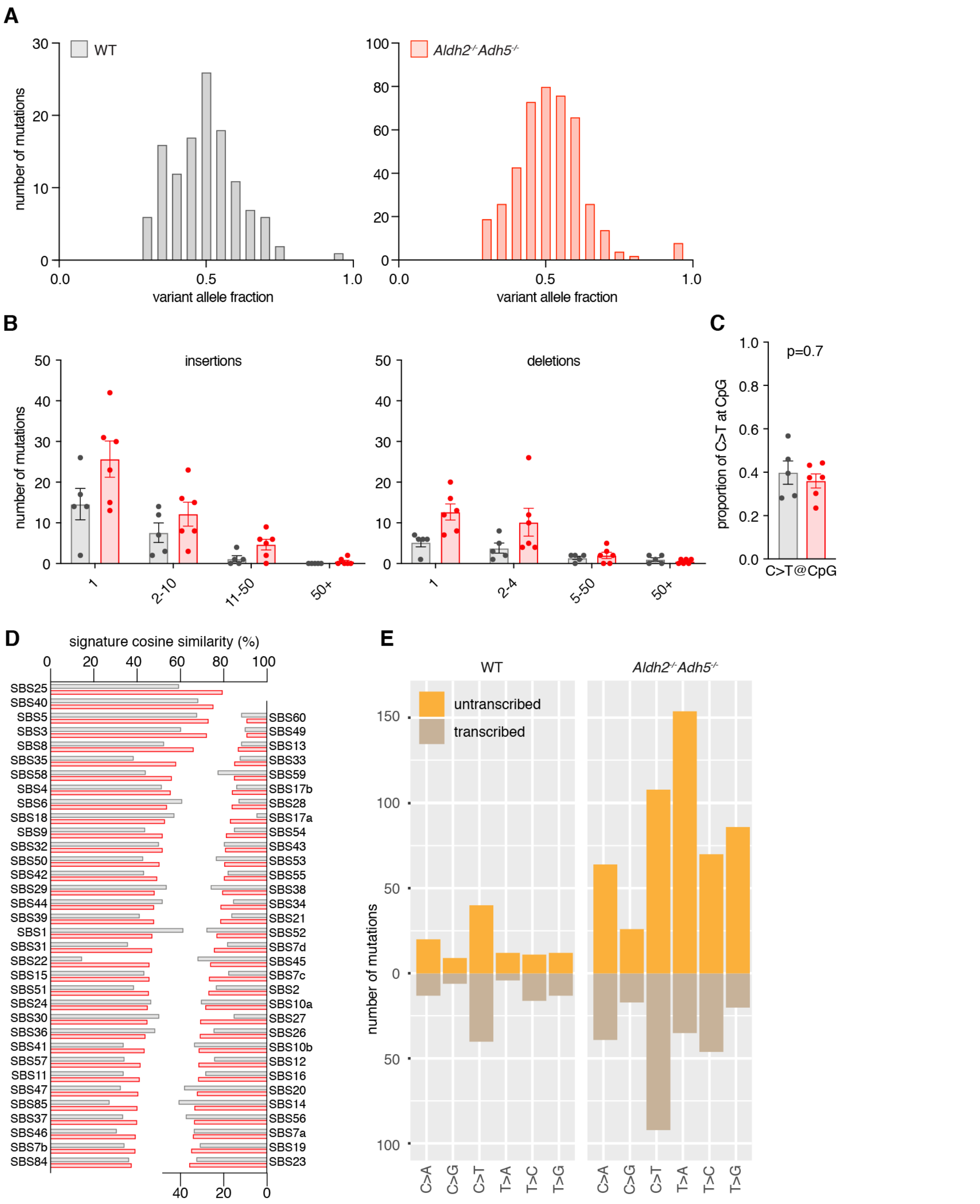

Figure S6, relating to Figure 6. Mutational signature of HSPC clones

(A) Representative histograms showing variant allele frequencies for final filtered (analyzed) variants of WT and *Aldh2^{-/-}Adh5^{-/-}* HSPC clones. **(B)** Size distribution of insertions (left) and deletions (right) in analyzed HSPC clones. **(C)** Proportion of C to T mutations occurring in CpG sequence context. **(D)** Cosine similarity between mutational profile of WT and *Aldh2^{-/-}Adh5^{-/-}*HSPCs and published COSMIC mutation signatures. **(E)** Histograms of single base substitutions falling into genes indicating whether the pyrimidine base of the pair is on the coding or noncoding strand.

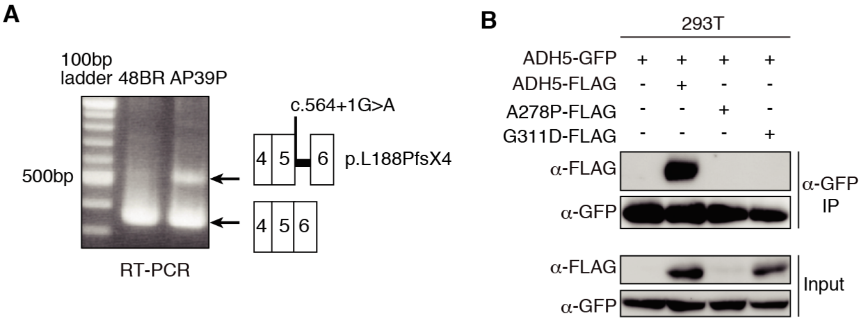

Figure S7, relating to Figure 7. Characterization of *ADH5* patient mutations. **(A)** RT-PCR analysis of *ADH5* transcripts in fibroblasts from patient P1. cDNA from patient or control 48BR cells was amplified using primers that cover exon 4 to 6 of *ADH5*. The c.564+1G>A mutation resulted in retention of intron 5 and p.L188PfsX4. **(B)** Effects of *ADH5* missense variants on expression and dimer formation. 293T cells transfected with indicated plasmids were lysed, subjected to coimmunoprecipitation by anti-GFP antibody, and precipitated proteins were detected by western blotting with anti-FLAG antibody. Note that the FLAG-tagged A278P ADH5 variant was barely detected in the lysate.

Table S1, related to Figure 1B.
Incidence of cancer-related deaths in *Aldh2*$^{-/-}$*Adh5*$^{-/-}$ mice.

**Number**	**Age (weeks)**	**Cause of death**
1	29	CD4^{+} T cell leukemia
2	36	Liver tumor
3	38	Liver tumor and leukemia
4	42	CD8^{+} leukemia and thymoma
5	43	Thymoma

Table S2, related to Figure 2.
Enriched GO terms of top 100 differentially expressed genes in the *Aldh2$^{-/-}$Adh5$^{-/-}$* erythroid progenitors (Ery3)

GO term_name	term_id	adjusted_p_value
intrinsic apoptotic signaling pathway in response to DNA damage	GO:0008630	0.0156173
organonitrogen compound biosynthetic process	GO:1901566	0.02766473
intrinsic apoptotic signaling pathway by p53 class mediator	GO:0072332	0.03454527
signal transduction by p53 class mediator	GO:0072331	0.04324361
sequestering of actin monomers	GO:0042989	0.04836535

Table S3, related to Figure 2.

Enriched GO terms of top 100 differentially expressed genes in the *Aldh2*$^{-/-}$*Adh5*$^{-/-}$ HSC cluster

GO term_name	term_id	adjusted_p_value
response to oxygen-containing compound	GO:1901700	1.05E-08
cellular response to oxygen-containing compound	GO:1901701	1.36E-07
response to organic substance	GO:0010033	1.51E-07
cellular response to chemical stimulus	GO:0070887	2.31E-07
cellular response to organic substance	GO:0071310	1.01992E-06
regulation of immune system process	GO:0002682	1.99803E-06
regulation of multicellular organismal process	GO:0051239	4.44607E-06
immune system process	GO:0002376	6.12521E-06
response to endogenous stimulus	GO:0009719	6.60435E-06
myeloid cell differentiation	GO:0030099	1.28529E-05
positive regulation of multicellular organismal process	GO:0051240	3.40627E-05
regulation of myeloid cell differentiation	GO:0045637	4.49602E-05
regulation of localization	GO:0032879	6.35689E-05
response to nitrogen compound	GO:1901698	8.19104E-05
response to organonitrogen compound	GO:0010243	0.000100321
cell differentiation	GO:0030154	0.000161578
regulation of hemopoiesis	GO:1903706	0.000183388
cellular developmental process	GO:0048869	0.000273058
regulation of developmental process	GO:0050793	0.000371603
regulation of multicellular organismal development	GO:2000026	0.000416546
cellular response to endogenous stimulus	GO:0071495	0.000615769
hemopoiesis	GO:0030097	0.00080195
positive regulation of cellular process	GO:0048522	0.000967844
negative regulation of cellular process	GO:0048523	0.001286093
regulation of cell migration	GO:0030334	0.001366403
regulation of biological quality	GO:0065008	0.001558247
hematopoietic or lymphoid organ development	GO:0048534	0.001599819
cell migration	GO:0016477	0.001861214
response to chemical	GO:0042221	0.002013742
localization of cell	GO:0051674	0.002034977
cell motility	GO:0048870	0.002034977
regulation of cell population proliferation	GO:0042127	0.002081353
negative regulation of multicellular organismal process	GO:0051241	0.002183892
heterophilic cell-cell adhesion via plasma membrane cell adhesion molecules	GO:0007157	0.002214536
cell population proliferation	GO:0008283	0.002418803
cellular response to calcium ion	GO:0071277	0.002628424
response to stress	GO:0006950	0.002668339
regulation of cell motility	GO:2000145	0.002785447
locomotion	GO:0040011	0.002986195
immune system development	GO:0002520	0.003542013
positive regulation of gene expression	GO:0010628	0.003877516
response to lipid	GO:0033993	0.004091222
positive regulation of developmental process	GO:0051094	0.004684961
positive regulation of macromolecule metabolic process	GO:0010604	0.00485466
regulation of locomotion	GO:0040012	0.005157507
response to lipopolysaccharide	GO:0032496	0.005387423
positive regulation of nitrogen compound metabolic process	GO:0051173	0.005442722
myeloid leukocyte differentiation	GO:0002573	0.005552054
positive regulation of cellular metabolic process	GO:0031325	0.005951117
cell surface receptor signaling pathway	GO:0007166	0.005964847
negative regulation of biological process	GO:0048519	0.006029472

GO term_name	term_id	adjusted_p_value
movement of cell or subcellular component	GO:0006928	0.006827294
regulation of cell activation	GO:0050865	0.007000214
response to molecule of bacterial origin	GO:0002237	0.007649681
regulation of cellular component movement	GO:0051270	0.00944446
positive regulation of metabolic process	GO:0009893	0.009520947
cellular response to glucose stimulus	GO:0071333	0.009643389
cellular response to hexose stimulus	GO:0071331	0.010038369
positive regulation of biological process	GO:0048518	0.010537982
cellular response to monosaccharide stimulus	GO:0071326	0.010868856
cell activation	GO:0001775	0.011690641
inflammatory response	GO:0006954	0.013768332
cellular response to carbohydrate stimulus	GO:0071322	0.015929948
positive regulation of immune system process	GO:0002684	0.016756211
positive regulation of cell-cell adhesion	GO:0022409	0.017292424
cell death	GO:0008219	0.017759915
response to external stimulus	GO:0009605	0.019372058
regulation of leukocyte activation	GO:0002694	0.019972363
cellular glucose homeostasis	GO:0001678	0.022029729
amyloid-beta clearance	GO:0097242	0.022835694
enzyme linked receptor protein signaling pathway	GO:0007167	0.024718074
leukocyte differentiation	GO:0002521	0.026421044
response to abiotic stimulus	GO:0009628	0.027722184
cellular response to metal ion	GO:0071248	0.030962982
MAPK cascade	GO:0000165	0.032933037
homeostatic process	GO:0042592	0.037793755
regulation of cell death	GO:0010941	0.038337117
regulation of transcription by RNA polymerase II	GO:0006357	0.039925822
signal transduction by protein phosphorylation	GO:0023014	0.042851358
defense response	GO:0006952	0.044757433
response to hormone	GO:0009725	0.047719264

Table S4, related to Figure 2.

Top 100 upregulated genes in HSC subclusters (relative to union of other subclusters). Genes used to assign lineage bias highlighted in bold.

	cluster 0	cluster 1	cluster 2	cluster 3	cluster 4	cluster 5	cluster 6
1	Ifitm1	Wfdc17	Cdk6	Fut8	Hist1h2bc	Ifit1	C1qa
2	Pdzk1ip1	**Ighm**	Plac8	Cdk6	Vim	Isg15	C1qb
3	Gng11	**Dntt**	**Mpo**	Nkg7	Ccnb2	Oasl2	C1qc
4	Mllt3	H2afy	Sell	Sdsl	Rps2	Rsad2	Apoe
5	Hacd4	Flt3	Ccl9	Muc13	Ptms	Ifi44	Ctsb
6	Ifitm3	Satb1	Nkg7	Eif5a	Hist1h1c	Ifitm3	Fcer1g
7	Malat1	Emb	Cd48	Nme1	Lgals1	Rtp4	Tmsb4x
8	**Procr**	Mef2c	Serpinb1a	Dctpp1	Cenpe	Trim30a	Psap
9	**Mpl**	Cd52	Tespa1	Ldha	H2afv	Iigp1	Tyrobp
10	Rbp1	Il12a	H2afy	Atp5g1	Txn1	Usp18	Marcks
11	Hlf	Ramp1	Prtn3	Cdca7	Dap	Ifit3	Selenop
12	Ltb	Ddx4	Adgrg3	Hmgb3	Hist1h4i	Slfn5	Axl
13	**Mecom**	Tmsb10	Bex6	Apoe	Rps17	Gm4951	Ctss
14	Socs2	Sdc1	Gm20342	Mif	Srgn	Irf7	Lgmn
15	Apoe	Wfdc18	BC035044	Gclm	Knstrn	Zbp1	Hpgd
16	Cdkn1c	Gpr171	Calr	Fabp5	Rpl41	Xaf1	Vcam1
17	Ly6a	Smad7	Cmtm7	Txn1	Cenpf	Bst2	Mrc1
18	Tbxas1	Ncf1	Cd34	Srsf3	Rpl14	Igtp	Fth1
19	Cd63	Rps24	Ctsg	Cd48	Cox6b2	Mndal	Sirpa
20	Gimap1	H2-Ob	Slco3a1	Srgn	Tmsb4x	Parp9	Aif1
21	Itsn1	Cd34	Tyrobp	Atpif1	Plac8	Gbp7	Fcna
22	Cavin3	Rasa4	Lat2	Vamp5	Cd9	Ifi203	Sdc3
23	Bex1	Notch1	Fut8	Tgfb1	Cdca8	Ly6a	Mafb
24	Tcf15	Cd37	Ndufa4	Sell	Mki67	Parp14	Csf1r
25	Esam	Sema3d	Bin1	Hnrnpd	Ddx39	Panx2	Trf
26	Car2	St8sia4	Phf14	Rps17	Rpl7a	Ly6e	Mpeg1
27	Mycn	Mn1	Zeb2	Ncl	S100a10	Samd9l	Cfp
28	Wfdc2	Ccl3	Atp8b4	Cd63	Atp5b	Eif2ak2	Ms4a7
29	Selenom	Il1r1	Fam117a	Srm	Hmgb1	Stat1	Cyba
30	Upp1	Cd69	Slc16a11	Nop58	Cenpa	Oas3	Cd68
31	Trim47	Cd33	Vim	Npm1	Rpl3	Ms4a6b	Hmox1
32	Grb10	Slc35d3	Itga4	Rpl41	Hsp90ab1	Serpina3g	Igf1
33	Krt18	Egfl7	S100a10	Ranbp1	Ube2c	Herc6	Gngt2
34	Nkx2-3	Cd53	Plek	Hsp90ab1	Myl12b	Isg20	Grn
35	Rpl21	Sox4	Spns3	Rps2	**Mpo**	Ifit3b	Maf
36	Angpt1	BC035044	Myb	Clec4e	Hmgb2	Ifih1	Cybb
37	Gimap5	Myl10	Tgfb1	Slc25a5	Cd48	Trim12c	Adgre4
38	Shisa5	Arpp21	Cd52	Ppp1r14b	Lockd	Irgm1	Cd5l
39	Samd12	Gm32554	Muc13	Snrpd1	Hist1h2ae	Ifit1bl1	Sat1
40	H2-K1	Camk1d	Ramp1	Ran	Lgals9	Sp100	Ccr3
41	Serpina3g	Gm5111	Ffar2	Ctla2a	Selenoh	Ifi47	Itm2b
42	Ptgs1	Btg2	Adgrl4	Vim	Spi1	Shisa5	Clec12a
43	Pf4	Hoxa9	Rab44	Runx3	Psrc1	Mx1	Cd300c2
44	Ndn	Samsn1	Ptma	Mcm3	H1f0	Ifi204	Clec4b1
45	Vamp5	Pou2f2	Bcl2	Srsf7	Rpl28	Dhx58	Lrp1
46	Plxdc2	Fbxw4	Mdga1	Calr	H1fx	Rnf213	Ctsc
47	Txnip	Dhrs3	Pdgfrb	Igfbp4	Csrp2	Phf11b	Lpl
48	Arhgef12	Tespa1	Myc	Anp32b	Ifi27l2a	H2-T22	Fyb
49	Nceh1	Lck	Tm6sf1	4930519L02Rik	Hmgb3	Slfn8	Ctsz
50	Gimap6	Lsp1	Irf2bp2	Hspd1	Gpx1	Ms4a4b	Clec4a1
51	Col4a2	Sdc4	Rps2	Rpl14	Hist3h2a	Trim30d	Cst3
52	Csgalnact1	Malat1	Slc4a8	Dach1	Stmn1	Trafd1	Hexa
53	Cish	Pgr	Sh2d5	Ptma	Gapdh	Ifi206	Pilra
54	Gm4951	Sstr2	Ndrg1	Itga2b	Tpx2	Ddx60	Adgre1
55	Uba7	AA467197	Igfbp4	Dut	Dtnbp1	Ms4a6c	Ctsh
56	Ccnd2	Maml3	Dock10	Ybx3	Cdca3	Tor3a	Clec4a3
57	Jam3	Plac8	F630028O10Rik	Ppia	Hist1h2ac	Gbp3	Pld3
58	Tie1	Shisa8	Taok3	Slc18a2	Prtn3	Ube2l6	Ly86
59	Clec1a	Tcf4	Elf1	C1qbp	H2afz	Zufsp	Cd163
60	Myl10	Cd27	Arl11	Bin1	Sell	Ddx58	Creg1

	cluster 0	cluster 1	cluster 2	cluster 3	cluster 4	cluster 5	cluster 6
61	Npdc1	Jakmip1	Gm15657	Set	Cmtm7	Parp12	Plbd1
62	Sult1a1	Nav1	Atf7ip	Hsp90aa1	Sec61b	Epsti1	Mertk
63	Plxnc1	Evl	Tap2	Fgf3	Gmfg	Dtx3l	Ckb
64	Rras	Rabgap1l	Sox4	Cmtm7	S100a6	Ms4a4c	Cd74
65	Nt5c3	Il17re	Cd244	Slc22a3	Polr2a	Samhd1	Frmd4b
66	Col4a1	Lax1	Mcm7	Hspe1	Coro1a	Gbp2	Fcgr3
67	Gucy1a1	Tbxa2r	Erp29	Nop10	Rpl24	Cxcl10	Pilrb2
68	Gata2	Mgat1	Asap1	Siva1	Ssr2	Trim25	Unc93b1
69	Slfn2	Ctss	Tnfaip8l2	Rpl28	Ccdc34	Oas2	Fgr
70	Gstm1	Tnip3	Tspo	Ssr4	Gpr146	Stat2	Timp2
71	Cers4	Ccl4	Dapp1	Rps27l	Calm1	Tspo	Pid1
72	Sgms1	Lztfl1	Cebpa	Myc	Rplp0	Trim12a	Fcgr1
73	Obscn	Ikzf1	Gmfg	Actb	Fth1	Parp11	Clec4a2
74	Bex4	Prr5	Dnmt1	Tacstd2	Rnf130	Gbp2b	Zeb2
75	Kcnk5	Phf14	Git2	mt-Nd1	Nucks1	Slfn9	Filip1l
76	Nrk	Basp1	Gpx1	Ybx1	Tspo	BC147527	Actb
77	Ankrd33b	Emp1	Hsp90ab1	Plac8	Tnfaip8l2	Mb21d1	Hebp1
78	Myct1	Scn1b	Adgrg1	Fkbp4	Cox5b	Ifi208	Itgad
79	Pbx1	Cdk19	Clec4e	Dtymk	Capg	Irgm2	Ccl6
80	Bdh2	Gem	Gm2a	Nolc1	Gm20342	Tuba1b	Fabp4
81	Gm973	Cmah	Rgs10	Ydjc	Cks2	Psme2b	Lgals3
82	Slc18a2	Stxbp4	Kcnq1ot1	Olfr417	Ccl9	Serpina3f	Cd300a
83	Ccdc112	Bmyc	Snx14	Lgals9	Hscb	Tgtp2	Lyz2
84	Ifitm2	Shisa5	Fam133b	Tkt	Hist1h1d	H2-T23	Serpinb6a
85	Fgd5	Foxp1	Gm26917	Gapdh	Calr	Ifitm1	Plxnb2
86	Hoxb2	Thbs1	Plppr3	Gata2	Nasp	Gbp6	Fcgrt
87	Unc45b	Pan3	Slc22a3	Rpl4	Tmem14c	Helz2	Lcp2
88	Cavin1	Lims1	Chdh	Pa2g4	Ccnd3	Fam241a	AB124611
89	Prex2	Bcl11a	Kit	Cox6b2	Mgea5	Ifi27l2a	Pld4
90	Serpinf1	Xist	Sgk3	Cpa3	Rnaseh2c	H2-Q4	Laptm5
91	Slc24a5	Adgrg3	Npm1	Zfpm1	Bex6	Letm2	Ear2
92	Aplp2	Clec2i	Sh3bgrl3	Cct8	Acsl5	Trim30c	Irf8
93	Lst1	Rcsd1	Ncl	Rfc2	Hsp90b1	Socs1	Aplp2
94	Hdgfl3	Sh3bgrl3	Tkt	Tipin	Rpl4	Sp110	Blvrb
95	Ctnnal1	Rhob	Parp8	Ifitm2	Tgfb1	Tnfsf10	Col14a1
96	Scarf1	Rgs2	Dach1	Gnl3	1500009L16Rik	Tgtp1	Cd81
97	Fam110c	Arhgdib	Macf1	S100a10	Rps27l	Camk2d	Slc11a1
98	Vwf	Rhoh	Wsb1	Serpina3g	Slc9a3r1	Ifi207	Pilrb1
99	Slamf1	Slc18a1	Fabp5	Rack1	Prdx4	Phf11d	Itm2c
100	Ecscr	Tmem108	Xist	H1fx	Rpl6	Uba7	Vsir

Table S5, related to Figure 4. Overexpressed DNA repair genes in *Aldh2$^{-/-}$Adh5$^{-/-}$* progenitors following compensation for cell cycle phase.

Cell cycle phase was determined (as per Figure S3E) for each single cell transcriptome. Comparison of cells at equivalent cell cycle phase between WT and *Aldh2$^{-/-}$Adh5$^{-/-}$* was performed to generate significantly overexpressed genes in GO term GO:0006281 (DNA repair) (fold change > 1.2 and adjusted *p* value *p*<0.1).

Gene	adjusted p value	log2 fold change
Pclaf	1.7921E-105	0.765312
Dntt	3.37977E-67	1.801023
Rfc2	1.33044E-41	0.286725
Xrn2	1.2133E-32	0.269335
Ier3	5.55277E-27	0.748824
Xpc	6.33631E-25	0.481973
Pold4	7.64881E-24	0.360293
Mcm3	8.23846E-23	0.300839
Rfc5	1.44011E-15	0.346084
4930447C04Rik	4.64894E-14	1.266671
Clspn	8.56797E-14	0.450605
Chaf1b	9.3004E-14	0.397027
Neil3	2.52424E-12	0.633323
Nsd2	6.02353E-10	0.327927
Hmga2	1.15329E-09	0.513935
Xrcc6	6.55307E-08	0.519498
Paxx	3.31423E-07	0.358693
Xpa	1.36276E-06	0.355939
Msh5	4.61548E-06	0.821602
Gins2	6.94867E-06	0.263547
Poll	9.72739E-06	0.558407
Rmi2	1.89807E-05	0.400336
Eya1	0.000121298	0.445072
Nudt1	0.00034174	0.274727
Parpbp	0.00074051	0.283223
Fancd2	0.001577611	0.339103
Spo11	0.001733193	1.360291
Pnkp	0.001894512	0.319521
Nthl1	0.002547337	0.576525
Esco2	0.002569652	0.284777
Rad51	0.002666466	0.34912
Rbbp8	0.003405985	0.292151
Mrnip	0.006031151	0.504284
Rnf169	0.01175469	0.391834
Polk	0.01650161	0.322304
Parp3	0.02550269	0.386784
Brca2	0.02641574	0.270288
Rad18	0.05740908	0.291865
Zfp365	0.06057999	1.358286
Mcm8	0.07663427	0.349946
Neil1	0.07741061	0.404398
Eme1	0.09406375	0.485893
Rad51c	0.09528128	0.555988

Table S6, related to Table 1. Allele frequency of ADH5 variants detected in seven cases with FA-like BMF syndrome

Genomic location (hg19)	Reference allele /Alternative allele	cDNA	Protein	Allele frequency (allele count/allele number) in HERPACC-2	Allele frequency (allele count/allele number) in gnomAD
chr4: 99997854	C/T	c.564+1G>A	p.L188PfsX4	not detected	not detected
chr4: 99996194	C/G	c.832G>C	p.A278P	0.0008 (7/8412)	0.0002 (4/17696 in East Asia)
chr4: 99996094	C/T	c.932G>A	p.G311D	not tested	not detected
chr4: 99993857	TC/T	c.966delG	p.W322X	0.0005 (4/8412)	0.0005 (9/17604 in East Asia)

*gnomAD database is available at http://gnomad.broadinstitute.org.
HERPACC-2, Hospital-based Epidemiologic Research Program at Aichi Cancer Center; gnomAD, Genome Aggregation Database

Table S7, related to STAR methods. Primer sequences in this study.

Cloning Primers for *mmAldh2*

cDNA	mmAldh2_cl_fwd	TTATATGCTAGCTCAGCCGCCGCCACCAGCGCGGTG
	mmAldh2_cl_rev	GATGGCGGATCCAAGCTTGCATGATTCTTACGAGTTCTTCTG TGGCACTT

Sequencing Primers for *ADH5* gene

Exon2 (containing initiating codon)	KD16-219	ATTAATCTGTATCTAAATTGGCTATTAAGT
	KD15-304	TCTACTCATCTATCCAGAGACCTCATTGTG
Exon3	KD16-220	CTTGTATCTGTACCTCTGAATTGCATGCAC
	KD16-221	TTAGATGATACCTATTCATAAATAGTGGGT
Exon4	KD16-222	ATCCGTTGACAGGAATTTGCAAGGGCTGAT
	KD16-223	GGTTAATGACCTAAATTATTAAATAATTC
Exon5 (c.564+1G>A)	KD15-315	AGTTGTGGCTGATATCTCTGTTGCTAAAAT
	KD15-316	ACTCATTCTACCAGAGTCAAGAGAATCACT
Exon6	KD16-224	AATATTTACTGGTCATTATTTTTAAAACAT
	KD16-225	TTTAATCTAAAACTGCACTTC
Exon7 (c.G832>C; c.G932>A)	KD15-357	AACTATGCCATCTCTAGAAGTTTCGCCAGC
	KD16-226	TAGGTGGCTGGGATTAAACATCTGCCAATG
Exon8 (c.966delG)	KD15-319	ATTTACTTCTTCACTGAAAGGTGTTGGTCA
	KD15-320	TTTGGACATATATTCAGACACCAACTTTGG
Exon9	KD15-323	AGAATGTAATAATGATGTTGAGTTTGAGGG
	KD15-324	AAGCTCTACGAGGCTGTGAGGTTGGAGGCG

Sequencing Primers for *ALDH2*2*

c.G1510>A	KD18-12	CAGGGATCCTGGCACATACTTGTTATCTTA
	KD18-13	ACGGATCCTGGTGAAAATCTGAAAAGATTC

Primers for RT-PCR

ADH5 (full-length)	KD16-155	CACCATGGCGAACGAGGTTATCAAGTGCA
	KD16-156	TTAAATCTTTACAACAGTTCGAATG
ADH5 (Exon4-Exon6)	KD16-199	CACTGTCATCCCACTTTACATCCCA
	KD16-200	TGCCAATCCGACTCCTCCCAGACA
GAPDH	KD12-215	GTCTCCTCTGACTTCAACAGCG
	KD12-216	ACCACCCTGTTGCTGTAGCCAA

***ADH5* site-directed mutagenesis**

c.G932>A	KD16-574	TGGTAACAGATCGCACATGGAAAGGCACTG
	KD16-565	CCATGTGCGATCTGTTACCAGCTGGAATGG
c.G832>C	KD16-239	GAGAGCACCACTTGAGGCATGTCACAAGGGCTGGGGCGTC
	KD16-240	TCAAGTGGTGCTCTCATGACCTTCACATTACCAATACATT
